# Clinical and cost-effectiveness of pharmacogenomic testing for anthracycline-induced cardiotoxicity in childhood cancer: a systematic review and meta-analysis

**DOI:** 10.3389/fphar.2025.1568320

**Published:** 2025-07-16

**Authors:** Ling Yin Fritz Wong, Alastair G. Sutcliffe, Carmen Lok Tung Ho, Yan Lu, Carrie L. Williams, Faiza Afzal, Mitana Purkayastha

**Affiliations:** ^1^ Department of Paediatrics and Child Health, University College London Medical School, London, United Kingdom; ^2^ UCL Great Ormond Street Institute of Child Health, University College London, London, United Kingdom; ^3^ Department of Paediatrics, University College London Hospital, London, United Kingdom; ^4^ Targeted Children’s Services, Waltham Forest, North East London NHS Foundation Trust, London, United Kingdom

**Keywords:** pharmacogenomics, polymorphism, anthracycline, chemotherapy, cardiotoxicity, childhood cancer, health economics, meta-analysis

## Abstract

**Background:**

Anthracyclines are widely used paediatric chemotherapy drugs, but anthracycline-induced cardiotoxicity (ACT) can cause heart failure in 16% of children. Previous studies have linked genetic variants to ACT and proposed pharmacogenomic testing for anthracycline-treated children; however, this approach remains unrealised. Therefore, this systematic review and meta-analysis evaluates the effectiveness of pharmacogenomic testing for ACT in childhood cancer.

**Methods:**

Nine bibliographic databases, three trial registers, reference lists and conference abstracts were searched from inception until October 2024. Two reviewers independently performed study selection, data extraction and quality assessment. Clinical effectiveness was defined as: 1) genetic associations, assessed using random-effects meta-analyses of odds ratios (OR) and mean differences (MD) with 95% confidence intervals (CI) for variants examined in ≥2 studies; and 2) prediction accuracy, measured using area under the receiver operating characteristic curve (AUC) of pharmacogenomic models. Cost-effectiveness was assessed using incremental cost-effectiveness ratio (ICER).

**Results:**

Among 1,215 de-duplicated records, we included 37 clinical effectiveness studies (26,446 patients). Five cardiotoxic (*ABCC2* rs8187710, *ETFB* rs79338777, *GPR35* rs12468485, *HNMT* rs17583889 and *UGT1A6* rs17863783; pooled OR range 1.84–6.12; CI range 1.04–18.56) and two cardioprotective (*GSTA2* rs2180314 and *HFE* rs1799945; pooled OR range 0.62–0.63; CI range 0.46–0.84) variants were significantly associated with ACT. Another cardioprotective variant, *ABCC5* rs7627754, increased left ventricular ejection fraction (MD 7.39%; CI 4.63%–10.14%) and fractional shortening (MD 5.04%; CI 2.00%–8.08%). Pharmacogenomic models using clinical and genetic variables (AUC range 0.67–0.87) showed higher accuracy in predicting ACT than those using clinical variables (AUC range 0.57–0.81) across five studies. We identified only one cost-effectiveness study (100 patients), showing one of these models reduced costs (−5.7%) and mortality (−17%) compared to standard care (ICER-negative). Overall, the evidence was graded as very-low-certainty across all outcomes due to imprecision, inconsistency and publication bias.

**Conclusion:**

Despite promising results, this review highlights the lack of robust evidence to support pharmacogenomic testing for ACT in children. Further cost-effectiveness studies and ethnically diverse prediction models are needed to demonstrate the impact of pharmacogenomic testing on ACT prognosis and clinical decision-making prior to adoption.

**Systematic Review Registration:**

PROSPERO identifier CRD42024557946.

## 1 Introduction

Anthracyclines, including doxorubicin, daunorubicin, epirubicin and idarubicin, are essential in chemotherapy regimens for nearly 60% of childhood cancers (van Dalen et al., 2014), contributing to improvements in 5-year survival rates from 58% in the 1970s to 83% in the 2010s (Lipshultz et al., 2014). However, these benefits are often offset by the increased risk of anthracycline-induced cardiotoxicity (ACT), a dose-dependent adverse effect resulting from disrupted anthracycline metabolism and transport, oxidative stress capacity, contractility and iron homeostasis (Ding et al., 2022; Cejas et al., 2024).

ACT can present acutely as reversible myocardial injury, or chronically as irreversible left ventricular dysfunction and cardiomyopathy within 1 year (early-onset) or more (late-onset) post-treatment (Cardinale et al., 2020). Diagnosis typically relies on clinical symptoms and reductions in left ventricular ejection fraction (LVEF) and fractional shortening (FS) (Bloom et al., 2016). In severe cases, the 5-year survival rate can decrease to <50%, with nearly 100% mortality being observed at 10 years in the absence of a heart transplant (Cejas et al., 2024). This poses a significant cardio-oncological challenge (Cardinale et al., 2016) as current management options such as dexrazoxane have limitations particularly in children (Reichardt et al., 2018). Up to 16% of anthracycline-treated children may develop heart failure (Kremer et al., 2002), complicated by risk factors including female sex, young age, high cumulative doses and chest radiation (Lipshultz et al., 2007; Tripaydonis et al., 2019). However, these clinical factors alone do not sufficiently explain the inter-patient variability in ACT, necessitating the investigation of the role of genetic factors (Al-Otaibi et al., 2022).

Previous studies have linked genetic variants to ACT through various pathophysiological mechanisms, particularly those involving anthracycline metabolism and transport. Examples include *UGT1A6* variants, which impair glucuronidation of anthracycline metabolites, increasing oxidative stress and triggering cardiomyocyte apoptosis (Aminkeng et al., 2017; Huang et al., 2022). *ABCC1* typically mediates efflux of toxic anthracycline metabolites, but its variants can reduce this function, resulting in intracellular drug accumulation in cardiac tissue (Jungsuwadee et al., 2009). In contrast, *SLC28A3* regulates anthracycline influx; its variants reduce cardiomyocyte uptake of anthracycline and provide cardioprotection (Li et al., 2022). Beyond anthracycline metabolism and transport, other pathways have also been implicated. *RARG* variants increase topoisomerase-IIβ levels, thereby increasing susceptibility to anthracycline-induced DNA damage (Aminkeng et al., 2015), while *ETFB* variants impair mitochondrial function, rendering cells more vulnerable to oxidative stress (Ruiz-Pinto et al., 2017a), both of which contribute to ACT. Genetic variants involved in sarcomere disruption, iron homeostasis and other pathways have also been implicated in ACT (Linschoten et al., 2018).

Pharmacogenomics is the study of how genes influence drug responses. It can be used to identify associated genetic variants (e.g., the single nucleotide polymorphisms [SNP] discussed above, discovered through candidate gene studies [CGS] and genome-wide association studies [GWAS]) (Alghamdi and Padmanabhan, 2014) and incorporate them into models to predict at-risk patients. This has been successful for chemotoxicities, notably *TPMT* and *DPYD* polymorphisms, reducing toxicity without compromising efficacy for patients receiving 6-mercaptopurine (Sing et al., 2015) and fluoropyrimidine (Henricks et al., 2018), respectively. *TPMT* genotyping demonstrated 90% sensitivity and 99% specificity in predicting enzyme activity (Schaeffeler et al., 2004), and cost-effectiveness analyses estimated 2,100 Euros (Great British Pounds [GBP] 2,776 in 2024) per life-year gained in leukaemia patients (van den Akker-van Marle et al., 2006). In contrast, despite the identification of numerous ACT-associated variants, pharmacogenomic testing for ACT remains relatively underutilised. Consensus on its clinical application is lacking, and commercially available tests have limited coverage of variants (Al-Otaibi et al., 2022).

Comparing the success of pharmacogenomic testing for chemotoxicities with that for ACT highlights differences in clinical and cost-effectiveness. The former can be assessed through two proxy measures: association and accuracy (Health Information and Quality Authority, 2018), with strong associations between variants and ACT enabling accurate predictions of at-risk patients. The latter is achieved when the incremental cost-effectiveness ratio (ICER) is below GBP 20,000 per quality-adjusted life year gained (National Institute for Health and Care Excellence, 2012) according to the National Institute for Health and Care Excellence (NICE) guidelines. With a threefold increase in ACT publications since 2002 (Wang et al., 2022), this review synthesises the latest evidence on the clinical and cost-effectiveness of pharmacogenomic testing for ACT in childhood cancer.

## 2 Methods

The review protocol was registered on PROSPERO (CRD42024557946) and the Preferred Reporting Items for Systematic reviews and Meta-Analyses (PRISMA 2020 statement) (Page et al., 2021; Rethlefsen et al., 2021) were followed (Supplementary Appendix S1).

### 2.1 Search strategy and study selection

Nine bibliographic databases (MEDLINE, Embase, CENTRAL, Scopus, CINAHL Plus, ProQuest Dissertations and Theses Global, PharmGKB, NHS Economic Evaluation Database and Cost-Effectiveness Analysis Registry) and three trial registers (ClinicalTrials.gov, WHO International Clinical Trials Registry Platform and International Standard Randomised Controlled Trial Number Registry) were searched from inception to 1 October 2024. Additional studies were identified *via* pharmacogenomics-specific organisations, conference abstracts and reference lists of relevant systematic reviews (Aminkeng et al., 2016; Conyers et al., 2017; Leong et al., 2017; Linschoten et al., 2018; Hurkmans et al., 2022; Ehrhardt et al., 2023). No search restrictions were imposed. Supplementary Appendix S2 presents the search strategies used.

Articles that met the following pre-determined eligibility criteria were included: i) children aged 0–18 years at the time of cancer diagnosis with any type of cancer, anthracycline use and ACT (“cases”); ii) exposure to any ACT-associated genetic variants, assessed by DNA-based techniques; iii) comparison with children with cancer and anthracycline use but without ACT (“controls”); and iv) primary studies including grey literature (conference abstracts and theses). Studies that examined epigenetic changes or used RNA-based or protein-based techniques were excluded as genomics focuses on changes in the DNA sequence. Studies defining ACT solely through cardiac biomarkers were excluded as biomarkers provide only supplementary diagnostic value compared to echocardiography (Bryant et al., 2007; Leerink et al., 2019; Michel et al., 2020). Case reports and case series were also excluded.

The primary outcome was the clinical effectiveness of ACT testing, defined as follows: i) association between genetic variants and occurrence of ACT, measured using odds ratio (OR); ii) association between genetic variants and cardiac function, measured using mean difference (MD) in LVEF and FS; and iii) accuracy of predicting ACT from genotypes using prediction models, measured using area under the receiver operating characteristic curve (AUC). The secondary outcome was the cost-effectiveness of ACT testing, measured using incremental costs and effects. Studies were classified as “clinical effectiveness” or “cost-effectiveness” based on the outcomes measured.

Duplicates were removed using EndNote 21, and all remaining records were screened using Rayyan (Ouzzani et al., 2016) with blinding. All titles and abstracts were assessed by two independent reviewers (LYFW, CLTH) and the full texts of potentially eligible records were evaluated for fulfilment of the eligibility criteria. Cohen’s kappa (κ) was used to quantify inter-rater agreement at each stage (McHugh, 2012). Disagreements at study selection were resolved through discussion meetings and consensus between both reviewers. The rationale for each inclusion or exclusion decision was documented to facilitate this discussion. A third-party adjudicator would have been consulted if consensus could not be reached; however, this was not required in our review.

### 2.2 Data extraction and analysis

Two reviewers (LYFW, CLTH) independently extracted data on cohort size; cohort name; demographics and chemotherapy characteristics; pharmacogenomic techniques for and effect measures of variants; co-exposures to chest radiation and cardioprotectants; ACT definition; and study design from included studies. Both reviewers compared the data extracted individually and resolved any disagreements through direct discussion to reach consensus, without the need for third-party adjudication. Missing data were requested from authors where necessary.

For clinical effectiveness studies, we conducted random-effects meta-analyses with inverse-variance weights for variants assessed in at least two studies. ORs and MDs in LVEF and FS were synthesised, producing pooled effect estimates with 95% confidence intervals (CI). Between-study variation was quantified using the Tau^2^ estimated by Restricted Maximum Likelihood. Heterogeneity was assessed using Cochran’s Q-test and *I* ^2^ statistic, although no fixed thresholds were used as these tests can be underpowered in small meta-analyses (von Hippel, 2015; Deeks et al., 2023). AUCs were not pooled to acknowledge the different variables used in prediction models. Meta-analyses were performed on Stata 18.0 and reported following the Meta-analysis of Observational Studies in Epidemiology (MOOSE) guidelines (Stroup et al., 2000).

Subgroup analysis by variant was conducted to investigate any heterogeneity due to variations in pathophysiological mechanisms between variants. Due to the limited methodological transparency of conference abstracts and theses, the primary meta-analyses included only peer-reviewed studies to minimise potential bias. Sensitivity analyses were performed by (1) expanding the analyses to all studies, including conference abstracts and theses (Scherer and Saldanha, 2019), and (2) restricting to overall low-risk-of-bias studies (Katikireddi et al., 2015), to evaluate the impact of study type and quality on the findings, respectively.

For cost-effectiveness studies, the costs were standardised by converting to Great British Pounds (GBP in 2024) using the CCEMG–EPPI Centre Cost Converter v.1.7 (Shemilt et al., 2010), and the incremental cost-effectiveness ratios (ICER) were then calculated.

### 2.3 Quality assessment

For clinical effectiveness studies, two reviewers (LYFW, CLTH) independently used the Risk Of Bias In Non-randomized Studies - of Exposures (ROBINS-E) tool (Higgins et al., 2024) to categorise the risk of bias into *Low*, *Some concerns*, *High*, or *Very high* across seven domains (e.g., confounding, participant selection and missing data). Conference abstracts providing insufficient information for risk-of-bias assessment were graded as *No information*. Risk-of-bias distributions were visualised using the R package “*robvis*” (McGuinness and Higgins, 2020). For cost-effectiveness studies, both reviewers independently used the Drummond checklist (Drummond et al., 2015) to grade study quality as *Yes/No* across 10 domains (e.g., measurement, valuation, time adjustment and incremental analysis for costs and consequences). Disagreements during quality assessment were resolved *via* discussion and consensus between both reviewers. The rationale for each domain’s grading was documented to facilitate transparency. No third-party adjudication was needed.

Both reviewers independently rated the certainty of evidence at outcome level using the Grading of Recommendations, Assessment, Development, and Evaluations (GRADE) system (Balshem et al., 2011). Certainty was downgraded or upgraded using a four-level scale (*High*, *Moderate*, *Low* or *Very low*) based on risk of bias, imprecision, inconsistency, indirectness and publication bias. Evidence from randomised trials starts at *High* certainty, while observational studies, including any associated economic data, starts at *Low* certainty due to residual confounding (Brunetti et al., 2013). Small-study effects were assessed using funnel plots, and asymmetry in funnel plots involving at least 10 studies were statistically confirmed using Egger’s test (p < 0.05) (Page et al., 2023).

## 3 Results

### 3.1 Study characteristics

**FIGURE 1:**
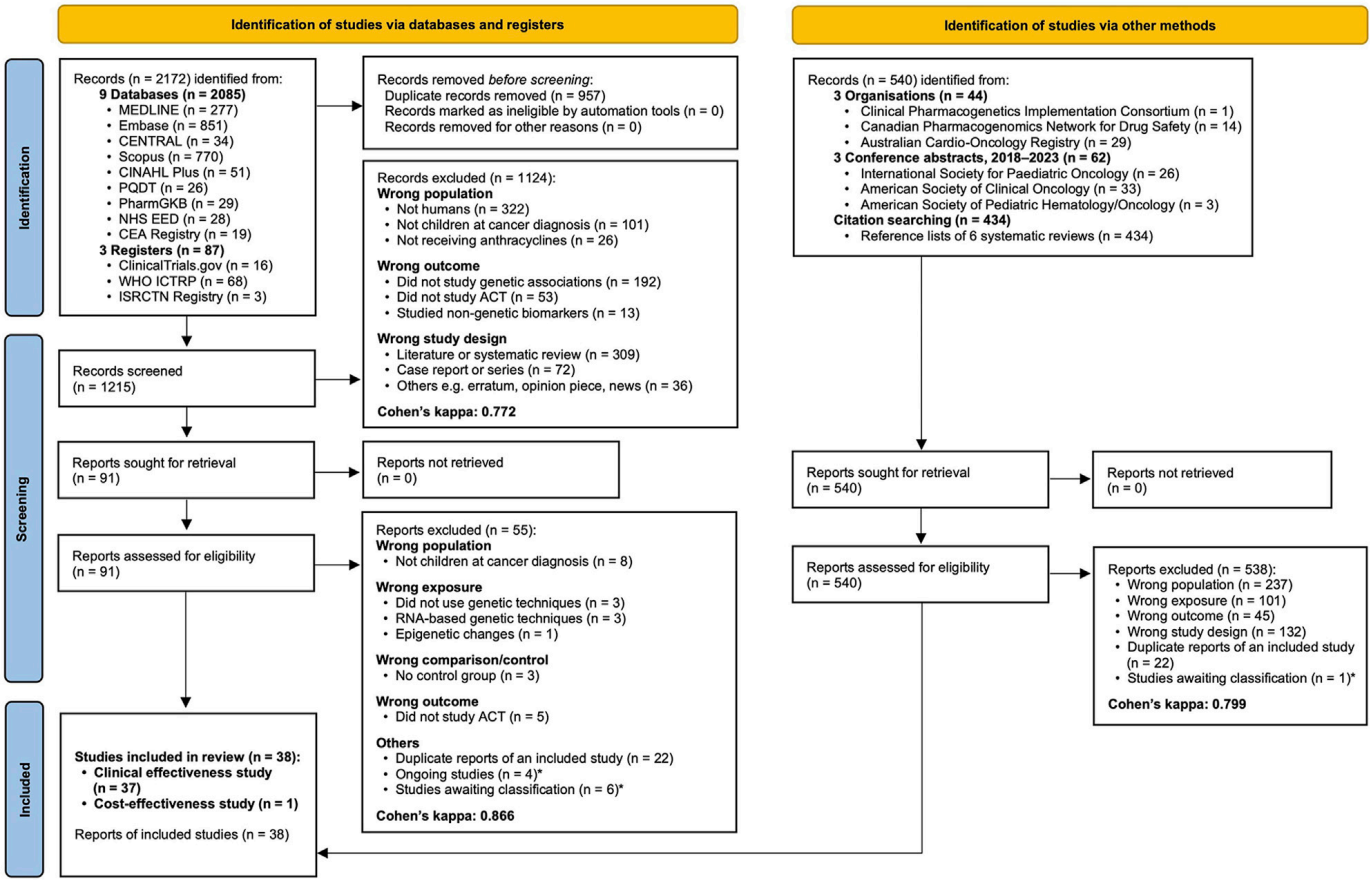
PRISMA 2020 flow diagram showing the study selection process. Cohen’s kappa additionally reported for inter-rater agreement. * Described in Supplementary Appendix S3. Abbreviations: CEA, Cost-Effectiveness Analysis; PQDT, ProQuest Dissertations and Theses Global.

The initial searches yielded 1,215 records after de-duplication (Figure 1). The title/abstract screening (κ = 0.772) and full-text screening (κ = 0.866) resulted in exclusion of 1,124 and 55 records, respectively. Supplementary searches yielded two conference abstracts for inclusion. Therefore, the final review included 38 studies (37 clinical effectiveness and one cost-effectiveness), totalling 26,546 childhood cancer patients or survivors. Supplementary Appendix S3 summarises the characteristics of the included studies. No authors provided sufficient information for inclusion upon data request.

#### 3.1.1 Characteristics of clinical effectiveness studies

This review included 25 case-control studies, six prospective cohort studies, five retrospective cohort studies and one cross-sectional study. Of these, four were conference abstracts (Giljeviae et al., 2010; Tonorezos et al., 2017; Wang et al., 2019; Scott E. et al., 2021) and two were theses (Liu, 2011; Boies, 2021). Almost equal numbers of studies were published in the 2010s (n = 18) and 2020s (n = 17), and the majority were conducted in North America (n = 27) and Europe (n = 6). In terms of genetic approaches, 23 were CGS, 12 were GWAS and two combined both approaches. Fourteen studies used replication cohorts to validate their genetic analyses (Visscher et al., 2012; Visscher et al., 2013; Wang et al., 2014; Aminkeng et al., 2015; Krajinovic et al., 2015; Visscher et al., 2015; Wang et al., 2016; Wang et al., 2019; Chaix et al., 2020; Petrykey et al., 2021; Sapkota et al., 2021; Sapkota et al., 2022; Sharafeldin et al., 2023; Wang et al., 2023).

Most studies recruited participants from pre-established multi-institutional cohorts, including the Canadian Pharmacogenomics Network for Drug Safety (CPNDS; n = 5) (Visscher et al., 2012; Visscher et al., 2013; Aminkeng et al., 2015; Visscher et al., 2015; Siemens et al., 2023); Children’s Oncology Group (COG-ALTE03N1; n = 6) (Liu, 2011; Blanco et al., 2012; Wang et al., 2014; Wang et al., 2016; Singh et al., 2020; Singh et al., 2023); Childhood Cancer Survivor Study (CCSS; n = 2) (Blanco et al., 2008; Sapkota et al., 2022); and both COG-ALTE03N1 and CCSS (n = 3) (Wang et al., 2019; Sharafeldin et al., 2023; Wang et al., 2023). These cohorts were predominantly made up of White or Caucasian participants. Reporting of participant age varied, with seven studies reporting age at the time of study participation (Giljeviae et al., 2010; Visscher et al., 2012; Visscher et al., 2013; Visscher et al., 2015; Gándara-Mireles et al., 2021; Vargas-Neri et al., 2022; Yunis et al., 2022), two reporting age at the start of treatment (Aminkeng et al., 2015; Dionne et al., 2017; Chaix et al., 2020), and the remaining reporting age at diagnosis. Median length of follow-up ranged from 20.5 days to 31 years.

Leukaemia was the most common cancer observed in 28 studies, while bone cancer (Giljeviae et al., 2010; Singh et al., 2020), sarcoma (Wang et al., 2016; Hildebrandt et al., 2017) and Hodgkin’s lymphoma (Tonorezos et al., 2017; Gündüz et al., 2024) each predominated in two studies. Doxorubicin (n = 24) was the most commonly examined anthracycline, followed by daunorubicin (n = 19). Of the 17 studies where participants had been treated with different anthracyclines, all except one (Semsei et al., 2012) expressed cumulative doses in doxorubicin isotoxic equivalent doses. The range of median cumulative doses was wider in cases (7.1–407.5 mg/m^2^) than controls (10.7–300 mg/m^2^).

Pharmacogenomic methods varied across studies. Most studies conducted DNA extraction using single biospecimens, including blood (n = 14), saliva (n = 2) or bone marrow (n = 1). Eleven studies collected two of these specimen types (Supplementary Appendix S3). Most studies used PCR-based methods (n = 17), microarrays (n = 13) and next-generation sequencing (n = 5) (Supplementary Appendix S3). In terms of quality control, 22 studies implemented at least one sample- or variant-level quality control measures, with the average sample call rate across 14 of these studies being 95.54%. All of them selected variants which were in Hardy-Weinberg equilibrium (Supplementary Appendix S3). Concomitant exposure to chest radiation was reported in 23 studies, while fewer studies reported cardioprotectant (e.g., dexrazoxane) use (n = 7).

Most studies diagnosed ACT based on echocardiograms (n = 12), cardiac signs/symptoms (n = 6) or both (n = 15). Echocardiographic parameters mainly included LVEF or FS, but their cut-off values varied. For LVEF, seven studies considered ≤40% as an ACT case (Blanco et al., 2012; Wang et al., 2014; Wang et al., 2016; Sapkota et al., 2022; Sharafeldin et al., 2023; Singh et al., 2023; Wang et al., 2023), while the highest cut-off value used was <57% (Gündüz et al., 2024). Cut-off values for FS were less varied, ranging from 24% to 30%. Eight studies reported excluding echocardiograms obtained less than 17 days (McOwan et al., 2020), 21 days (Visscher et al., 2012; Visscher et al., 2013; Visscher et al., 2015; Vargas-Neri et al., 2022; Siemens et al., 2023) or 30 days (Ruiz-Pinto et al., 2017a; Ruiz-Pinto et al., 2017b) post-treatment to rule out confounding by acute reversible ACT.

#### 3.1.2 Characteristics of the cost-effectiveness study

The economic component of this review included one study (Dionne et al., 2017) that conducted a decision model cost-effectiveness analysis comparing the costs and effects of pharmacogenomics-based risk classification and usual care. It was conducted in a hypothetical Canadian cohort of 100 childhood cancer patients with mostly leukaemia. Dexrazoxane use and a symptom-based ACT definition were reported.

### 3.2 Study quality

**FIGURE 2:**
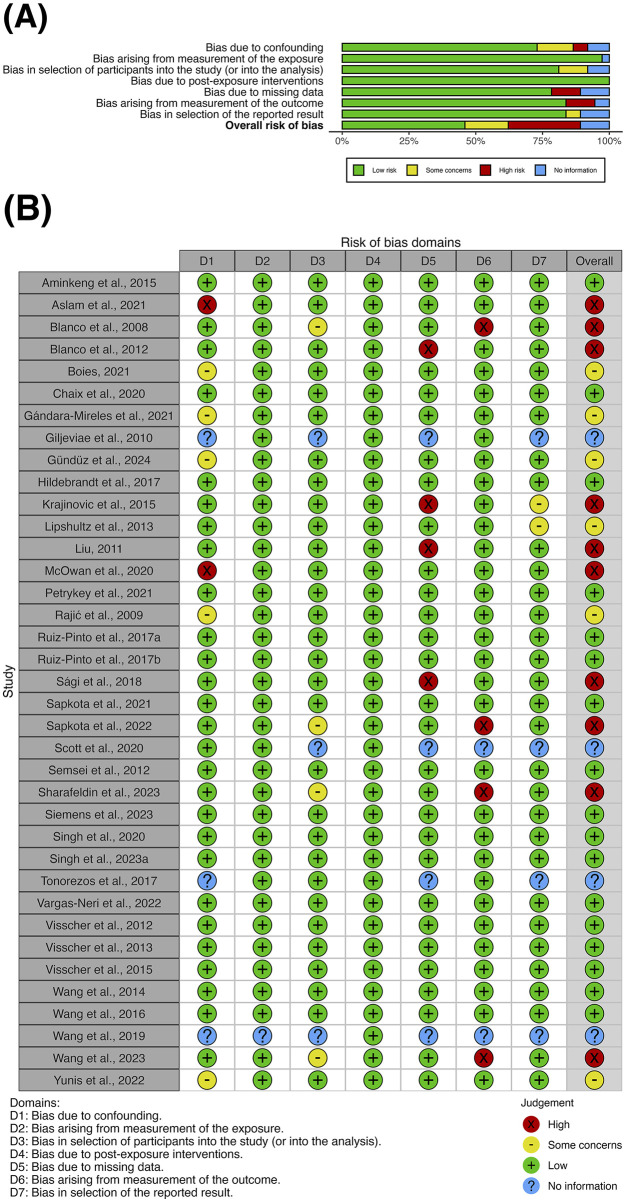
**(A)** Summary plot and **(B)** traffic-light plot for the risk-of-bias assessment of clinical effectiveness studies using the ROBINS-E tool at study level.

Evaluation of the clinical effectiveness studies using the ROBINS-E tool identified 17 studies with *low* overall risk of bias (Figure 2A). Six studies were assessed as having *some concerns* about bias primarily due to confounding, while 10 studies carried *high* risk of bias mainly due to missing data and biased outcome measurement (Figure 2B). The overall risk of bias could not be assessed in four conference abstracts due to insufficient detail on confounding, missing data and selective reporting. No studies were evaluated as having *very high* risk of bias. The cost-effectiveness study was considered high quality, fulfilling nine out of 10 domains in the Drummond checklist, with the unmet domain due to insufficient consideration of uncertainty in the cost-effectiveness estimates. Supplementary Appendix S4 explains the criteria used for the quality assessment above.

**FIGURE 3:**
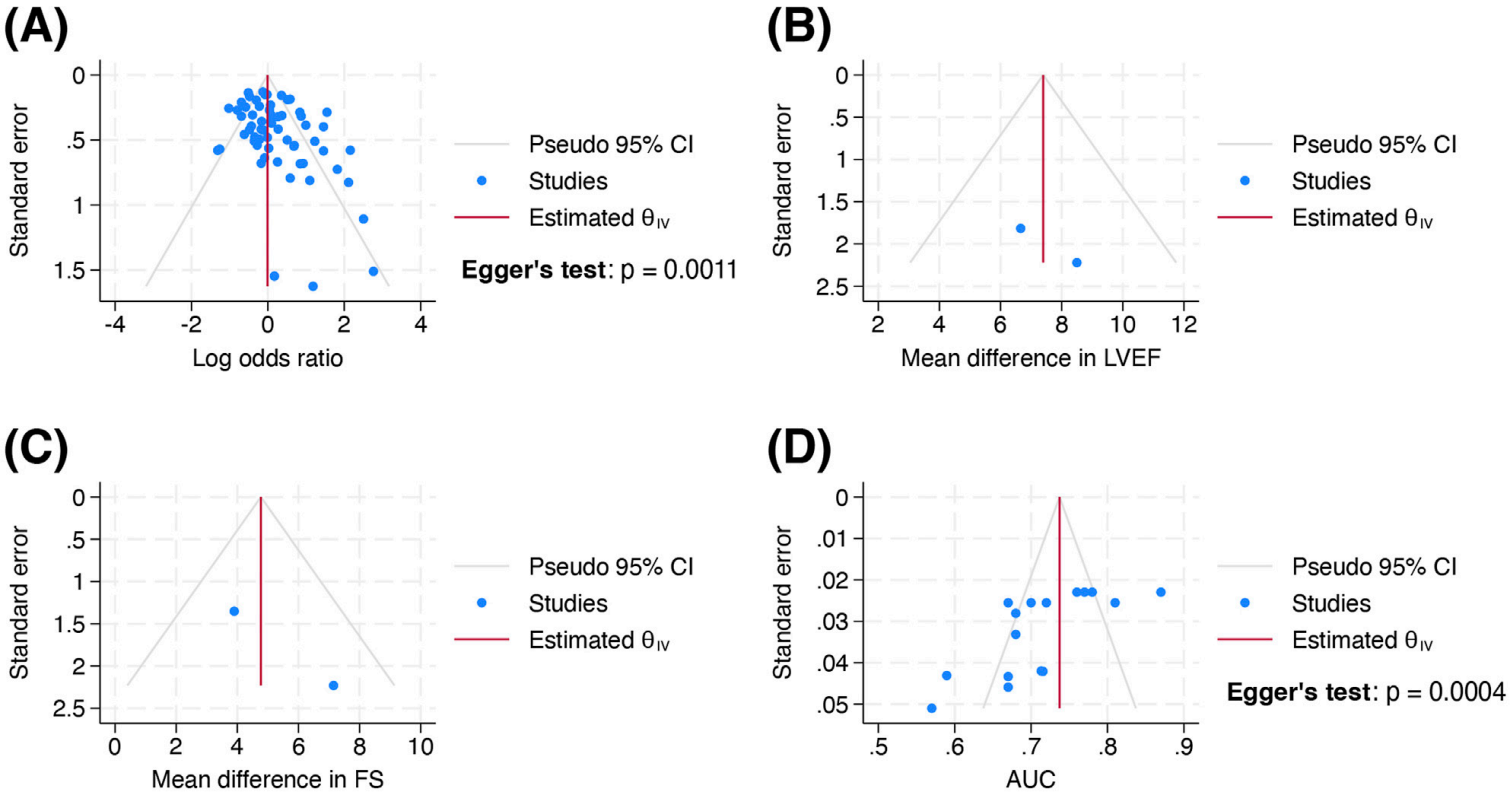
Funnel plots of standard error by **(A)** log odds ratio, **(B)** mean difference in left ventricular ejection fraction, **(C)** mean difference in fractional shortening and **(D)** area under the receiver operating characteristic curve in the body of evidence at outcome level. Wang et al., 2019 was excluded from Figure 3D as it did not report 95% confidence interval or standard error for AUC. Abbreviations: AUC, area under the receiver operating characteristic curve; FS, fractional shortening; LVEF, left ventricular ejection fraction.

Visual inspection of funnel plots revealed asymmetry in Figures 3A,D, but not in Figures 3B,C. Egger’s test was performed for outcomes involving at least 10 studies: OR (p = 0.0011) and AUC (p = 0.0004). For the analysis involving OR, including conference abstracts and theses did not reduce the small-study effects on visual inspection and Egger’s test (Supplementary Figure S1A). However, subgroup analysis by variant performed with or without inclusion of these sources (Supplementary Figures S2 and S3), showed no notable small-study effects on visual inspection. Egger’s test was not conducted as each subgroup comprised less than 10 studies. Therefore, potential publication bias may exist in the overall evidence for ACT variants, but not in the evidence for each individual variant. For the analysis involving AUC, both visual inspection of Figure 3D and statistical confirmation provided evidence of publication bias due to small-study effects.

### 3.3 Clinical effectiveness of pharmacogenomic testing for ACT

The included studies examined a total of 193 unique variants involving 147 genes for their association with ACT occurrence and cardiac function. All were SNPs except *GSTM1* deletion. Of these variants, 22 were investigated in at least two studies and were meta-analysed as individual subgroups.

#### 3.3.1 Association of genetic variants with ACT occurrence

**FIGURE 4:**
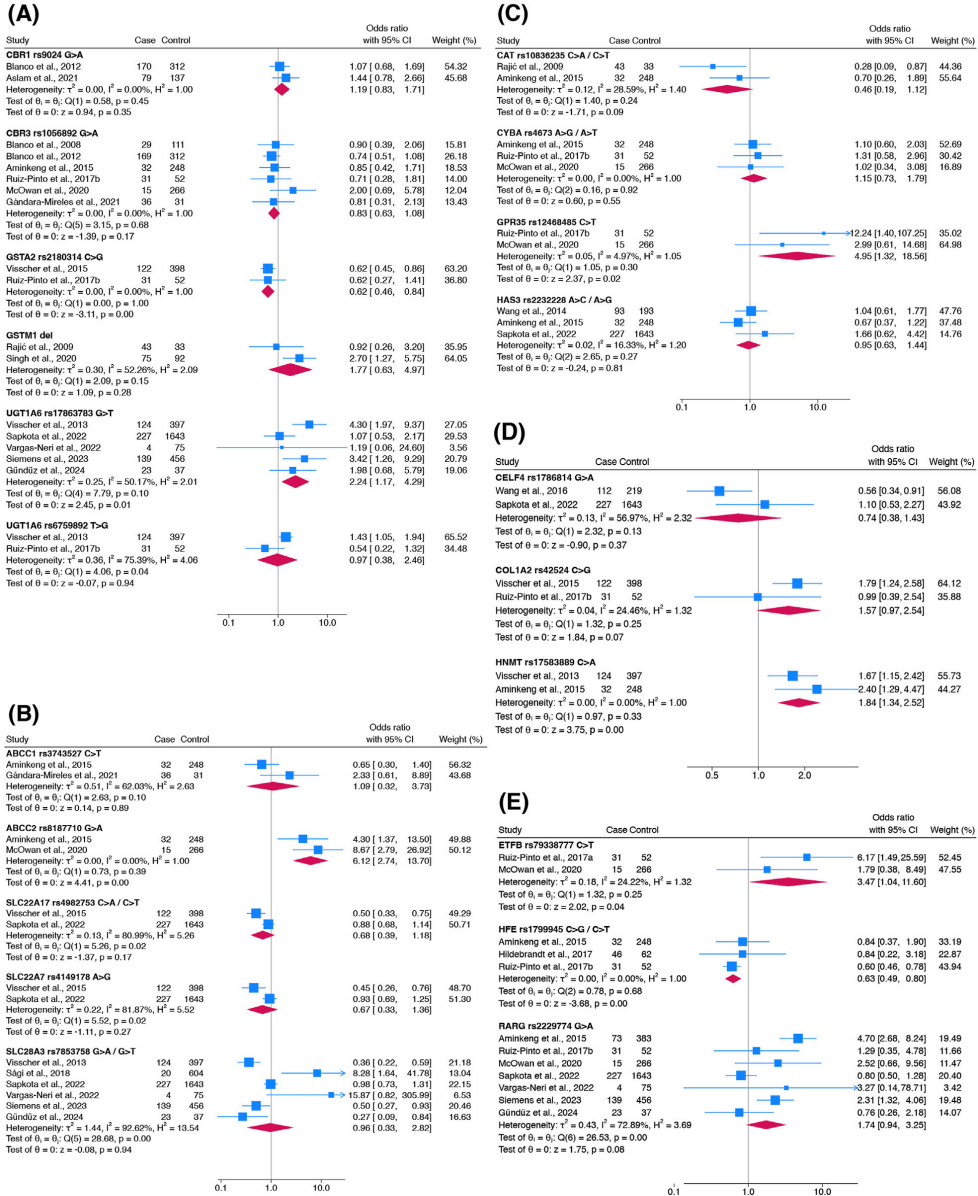
Forest plots showing the association of genetic variants responsible for **(A)** anthracycline metabolism, **(B)** anthracycline transport, **(C)** oxidative stress capacity, **(D)** contractility and **(E)** other functions with ACT occurrence. Other functions include mitochondrial function (*ETFB*), iron homeostasis (*HFE*) and DNA damage (*RARG*). Haldane-Anscombe correction was applied to zero-cell counts in Vargas-Neri et al., 2022.

Twenty-one variants were meta-analysed for their association with ACT occurrence and classified based on their gene functions (Figures 4A–E). Variants were categorised as cardiotoxic (pooled OR > 1) or cardioprotective (pooled OR < 1).

Five cardiotoxic variants were identified: *ABCC2* rs8187710 (pooled OR 6.12; 95% CI 2.74–13.70), *ETFB* rs79338777 (3.47; 1.04–11.60), *GPR35* rs12468485 (4.95; 1.32–18.56), *HNMT* rs17583889 (1.84; 1.34–2.52) and *UGT1A6* rs17863783 (2.24; 1.17–4.29). These variants exhibited a range of heterogeneity (*I* ^2^ = 0–50%) and had distinct gene functions. Two cardioprotective variants were identified: *GSTA2* rs2180314 (0.62; 0.46–0.84) and *HFE* rs1799945 (0.63; 0.49–0.80). Both variants had similar pooled ORs with no heterogeneity detected (*I* ^2^ = 0%). Apart from *UGT1A6* rs17863783 and *HFE* rs1799945, each variant was investigated in only two studies.

No significant association was found in the other 14 variants (Figure 4). These included the most studied variant, *RARG* rs2229774 (7 studies; pooled OR 1.74, 95% CI 0.94–3.25). However, sensitivity analyses including only studies with overall low risk of bias showed that this effect estimate changed from non-significant to significant (4 studies; pooled OR 2.83, 95% CI 1.53–5.24). Results of the sensitivity analysis with inclusion of conference abstracts and theses were consistent among the variants (Supplementary Figure S4). *SLC28A3* rs7853758 (pooled OR 0.96, 95% CI 0.33–2.82) was examined in six studies.

Association estimates tended to be higher in magnitude and significance in earlier discovery studies compared to subsequent replication studies. For example, the earliest study examining *RARG* rs2229774 (Aminkeng et al., 2015) reported a large and significant effect size (OR 4.70, 95% CI 2.68–8.24) which was not replicated in subsequent studies (Figure 4E). This temporal pattern was also observed in the most commonly studied variants including *UGT1A6* rs17863783 (Figure 4A) and *SLC28A3* rs7853758 (Figure 4B), where the first effect estimates reported by Visscher et al. (2013) (*UGT1A6* rs17863783: OR 4.30, 95% CI 1.97–9.37; *SLC28A3* rs7853758: OR 0.36, 95% CI 0.22–0.59) were not replicated in subsequent studies.

We downgraded the certainty of evidence for the genetic association with ACT to *very low* due to imprecision and inconsistency in some variants (−1 level as some variants had wide confidence intervals and considerable heterogeneity *I* ^2^ > 75%).

#### 3.3.2 Association of genetic variants with cardiac function

ACT was alternatively defined as a decrease in LVEF or FS in four studies (Semsei et al., 2012; Lipshultz et al., 2013; Krajinovic et al., 2015; Petrykey et al., 2021). Only one variant, *ABCC5* rs7627754, was examined in at least two studies for its association with these echocardiographic outcomes.

**FIGURE 5:**
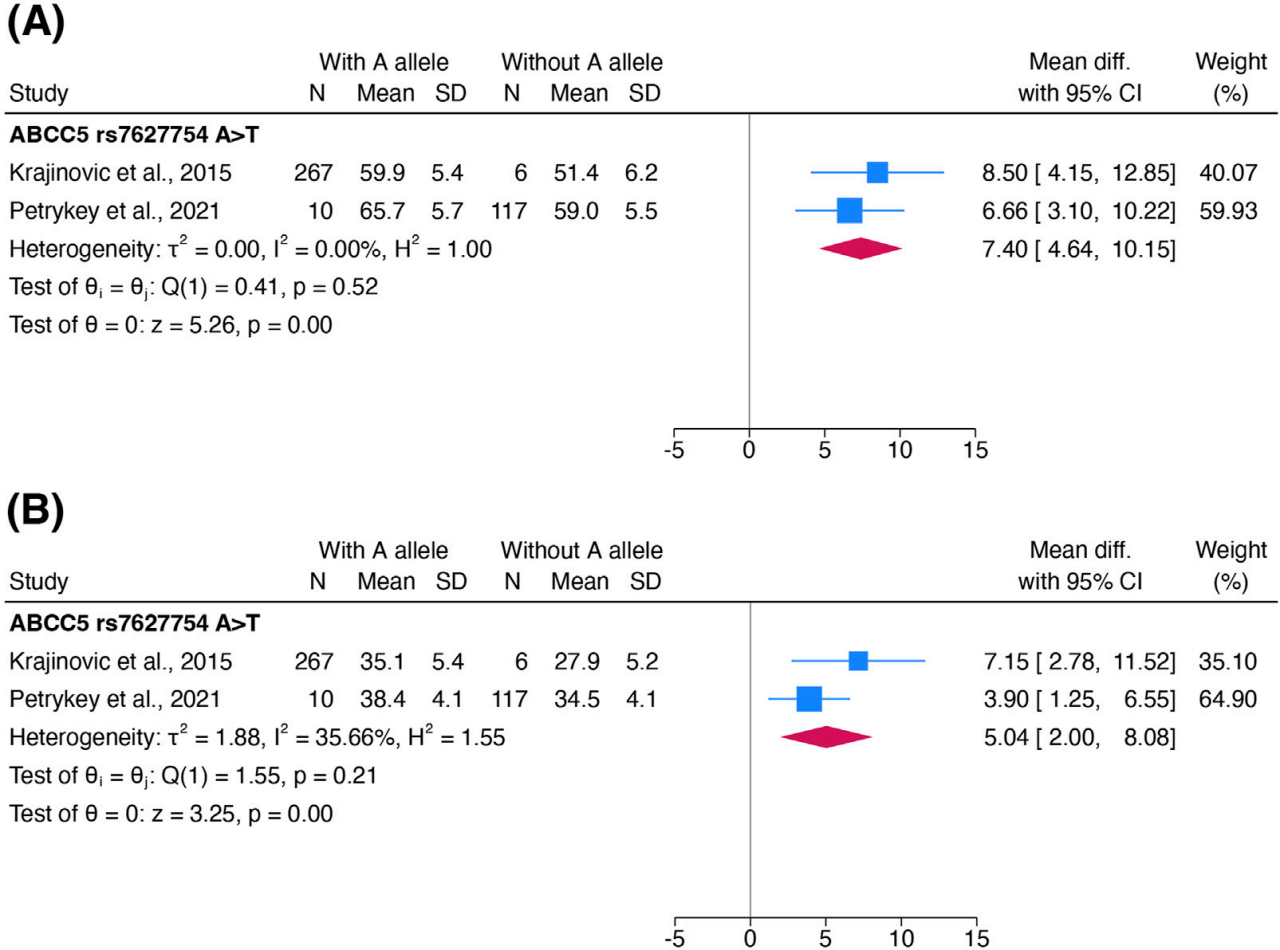
Forest plots showing the association of the A allele in *ABCC5* rs7627754 with **(A)** left ventricular ejection fraction and **(B)** fractional shortening. Standard deviations of LVEF and FS in both cohorts in Krajinovic et al., 2015 were estimated and combined into one cohort to avoid unit-of-analysis issues. Abbreviations: CI, confidence interval; FS, fractional shortening; IV, inverse-variance weights; LVEF, left ventricular ejection fraction; SD, standard deviation.

Patients with an A allele on *ABCC5* rs7627754 had a higher mean LVEF (pooled MD 7.39%; 95% CI 4.63%–10.14%) and higher mean FS (pooled MD 5.04%; 95% CI 2.00%–8.08%) than those without (Figures 5A,B). Both meta-analyses showed that the AT/AA genotype in *ABCC5* rs7627754 was cardioprotective. Sensitivity analyses did not change the significance or direction of these effect estimates.

We downgraded the certainty of evidence for cardiac function to *very low* due to risk of bias (−1 level as Krajinovic et al., 2015 was considered at high risk of bias).

#### 3.3.3 Accuracy of pharmacogenomics-based prediction models for ACT

Of the 22 meta-analysed variants, four cardiotoxic (*CYBA* rs4673, *HAS3* rs2232228, *HNMT* rs17583889 and *UGT1A6* rs17863783) and five cardioprotective (*CELF4* rs1786814, *SLC22A17* rs4982753, *SLC22A7* rs4149178, *SLC28A3* rs7853758 and *UGT1A6* rs6759892) variants were included into polygenic prediction models for ACT across five studies (Visscher et al., 2012; Visscher et al., 2013; Visscher et al., 2015; Wang et al., 2019; Chaix et al., 2020). All studies predicted ACT using a clinical model (with clinical variables only) and a combined model (clinical and genetic variables). Clinical variables were similar across studies, while genetic variables differed (Table 1). All models were evaluated in discovery and replication cohorts. All studies, except for the conference abstract by Wang et al. (2019), had overall low risk of bias and used an initial genetic association investigation to select their genetic variables.

**TABLE 1:** Clinical variables, genetic variables and area under the receiver operating characteristic curve (AUC) of the prediction models in five studies.

Study	Cohort size (n)	Clinical variables	Genetic variables				Clinical model AUC (95% CI)	Genetic model AUC (95% CI)	Combined model AUC (95% CI)
Chaix et al. (2020)	*Testing set:* 1000 patient replicates generated from bootstrapping *Training set:* Excluded^a^	• **Ethnicity** • **Sex** • **Age at start of treatment** • **Cumulative anthracycline dose** • **Chest radiation** • Dexrazoxane use • Length of follow-up	31 SNVs: *ATG4C* *ATP2B2* *CABYR* *CBLB* *CDIP1* *CELF4* *CHAD* *DYNAP*	*ELAC2* *FAM83C* *GJB7* *JAGN1* *KDM4B* *LRRFIP2* *MCU* *NOXO1*	*NUGGC* *ODF3* *OR1D2* *PIK3R2* *PMS1* *RGS3* *SAC3D1* *SEC62*	*SIM2* *SIMC1* *TGM3* *UACA* *USP42* *ZNF71* *ZNF827*	0.59 (0.51–0.67)	0.71 (0.63–0.80)	0.72 (0.63–0.80)
Visscher et al. (2012)	Cases, 77 Controls, 254	• **Ethnicity** • **Sex** • **Age at start of treatment** • **Cumulative anthracycline dose** • **Chest radiation**	9 SNPs: *ABCB4* rs1149222 *ABCC1* rs4148350 *FMO2* rs2020870 *HNMT* rs17583889 *SLC10A2* rs9514091		*SLC28A3* rs4877847 ***SLC28A3* rs7853758** *SPG7* rs2019604 *UGT1A6* rs6759892		0.68 (0.61–0.74)	0.81 (0.76–0.86)	0.87 (0.82–0.91)
Visscher et al. (2013)	Cases, 46 Controls, 131						0.67 (0.58–0.75)	0.57 (0.47–0.67)	0.67 (0.58–0.76)
Visscher et al. (2015)	Cases, 122 Controls, 398		5 SNPs: ***ABCB4* rs4148808** ***SLC28A1* rs2305364** ***SLC28A3* rs7853758**		***SULT2B* *1* rs10426377** ***UGT1A6* rs17863783**		0.68 (0.62–0.73)	0.67 (0.62–0.72)	0.76 (0.71–0.80)
Visscher et al. (2015)	Cases, 122 Controls, 398		6 SNPs: ***ABCB4* rs4148808** *SLC22A17* rs4982753 ***SLC28A1* rs2305364**		***SLC28A3* rs7853758** ***SULT2B* *1* rs10426377** ***UGT1A6* rs17863783**		0.68 (0.62–0.73)	0.70 (0.65–0.75)	0.77 (0.72–0.81)
Visscher et al. (2015)	Cases, 122 Controls, 398		7 SNPs: ***ABCB4* rs4148808** *SLC22A17* rs4982753 *SLC22A7* rs4149178 ***SLC28A1* rs2305364**		***SLC28A3* rs7853758** ***SULT2B* *1* rs10426377** ***UGT1A6* rs17863783**		0.68 (0.62–0.73)	0.72 (0.67–0.77)	0.78 (0.74–0.83)
Wang et al. (2019) [Conf Abs]	Cases, 155 Controls, 256	• **Ethnicity** • **Sex** • **Age at cancer diagnosis** • **Cumulative anthracycline dose** • **Chest radiation** • Diabetes, hypertension, dyslipidaemia	5 SNPs: *ABCC1* rs11864374 *CELF4* rs1786814 *CYBA* rs4673		*HAS3* rs2232228 *NQO1* rs1800566		0.7677 (95% CI NR)	NR	0.8138 (95% CI NR)

Bold text indicates the common clinical or genetic variables identified in at least three studies.

Abbreviations: AUC, area under the receiver operating characteristic curve; CI, confidence interval; Conf Abs, conference abstract; NR, not reported; SNP, single nucleotide polymorphism; SNV, single nucleotide variant.

^a^ Training set in Chaix et al., 2020 was excluded due to its main purpose of training the performance of the prediction model, thus we considered it as irrelevant.

Studies reported AUC as their primary measure of accuracy. Overall, the combined model produced a higher AUC (AUC range 0.67–0.87) than their respective clinical (AUC range 0.59–0.77) or genetic (AUC range 0.57–0.81) counterparts (Table 1). Interestingly, the most accurate (AUC 0.81, 95% CI 0.76–0.86) and least accurate (AUC 0.57, 95% CI 0.47–0.67) genetic models used the same variables. Similarly, the most accurate (AUC 0.87, 95% CI 0.82–0.91) and least accurate (AUC 0.67, 95% CI 0.58–0.76) combined models shared the same variables.

We downgraded the certainty of accuracy evidence to *very low* due to inconsistency and publication bias (−1 level due to extreme values in AUC between models despite using the same variables, and suspicion of publication bias in Figure 3).

### 3.4 Cost-effectiveness of pharmacogenomic testing for ACT

Being the most accurate, the combined model reported by Visscher et al. (2012) (AUC 0.87, 95% CI 0.82–0.91) was subsequently studied by Dionne et al. (2017) for its cost-effectiveness. Using the model’s prediction, patients were stratified into three risk categories and treated according to category-specific guidelines.

Costs in cardiology consultation, electrocardiogram, echocardiogram, drugs and heart transplantation were collected. The cost of pharmacogenomic testing was estimated at CA$100 per patient (GBP 71.76 in 2024). Effects were measured in terms of mortality rate and potential years of life lost (PYLL). The study adopted a healthcare provider perspective and modelled costs and effects over a lifetime horizon with discounting at 3.5%.

For patients across all categories, authors estimated a reduction in total costs (−5.7%; MD CA$ −495, GBP −355.19 in 2024) and deaths (−17%; MD −0.0118) per patient in the model with pharmacogenomics-based risk classification, when compared to usual care. For the high-risk subgroup, a larger reduction in total costs and deaths was estimated (Table 2). No confidence intervals or p-values were reported.

**TABLE 2:** Incremental costs and effects of the care with pharmacogenomics-based risk classification compared to usual care per patient in Dionne et al., 2017.

	Care with pharmacogenomics-based risk classification compared to usual care		
Risk category	Change in average lifetime cost per patient (incremental cost)	Death averted per patient (incremental effect)	Cost per death averted (ICER)^a^
All patients	CA$ 495 saved	0.0118	−CA$ 41,949.15
High-risk	CA$ 1858 saved	0.0503	−CA$ 36,938.37
Intermediate-risk	CA$ 854 increased	0	N/A
Low-risk	CA$ 421 saved	0	N/A

Risk classification was based on the combined model using both clinical and genetic variables as described in Visscher et al., 2012. It defined risk categories based on their predictive value for ACT: high-risk (>0.5), intermediate-risk (0.1–0.5) and low-risk (<0.1).

Abbreviations: CA$, Canadian Dollar in 2017; ICER, incremental cost-effectiveness ratio; N/A, not applicable.

^a^ ICERs were manually calculated from the reported data; ICER was undefined when zero deaths were averted.

The negative ICER for all patients and high-risk patients showed that care with pharmacogenomics-based risk classification was the economically dominant strategy (Cohen and Reynolds, 2008) as it was less costly and more effective.

We downgraded the certainty of cost-effectiveness evidence to *very low* due to imprecision (−1 level as the cost-effectiveness estimate came from only one study with small sample size (Brunetti et al., 2013)). We could not assess inconsistency and publication bias with only one study.

## 4 Discussion

This systematic review and meta-analysis evaluated the clinical effectiveness and cost-effectiveness of pharmacogenomic testing for ACT in childhood cancer patients. From the 38 included studies, we identified five cardiotoxic (*ABCC2* rs8187710, *ETFB* rs79338777, *GPR35* rs12468485, *HNMT* rs17583889 and *UGT1A6* rs17863783) and three cardioprotective variants (*GSTA2* rs2180314, *HFE* rs1799945 and *ABCC5* rs7627754) that were significantly associated with ACT and cardiac function. Although most of these variants were involved in shared pathophysiological pathways for anthracycline metabolism, anthracycline transport and oxidative stress capacity (Ding et al., 2022; Cejas et al., 2024), effect sizes were heterogeneous and moderate across variants. Some of these variants were subsequently integrated into prediction models that exhibited moderate accuracy. Combining genetic variables into clinical prediction models increased their ability to discriminate between cases and controls. Application of one of these models for risk classification of ACT showed that it was more cost-effective than usual care, although this was reported by only one study. Overall, less than half of the individual studies were judged as having low risk of bias. Therefore, given the very-low-certainty evidence with suspected publication bias, the findings of this review should be interpreted with caution.

Considering all these perspectives, we believe the current evidence is inconclusive to support the implementation of pharmacogenomic testing for ACT in clinical practice. The “bench to bedside” translation of confirmed genetic associations with ACT into clinically useful pharmacogenomic tests has yet to be realised. Firstly, compared to established pharmacogenomic tests such as those for *TPMT* polymorphisms, the variants implicated in ACT showed weaker associations (Nguyen et al., 2011). However, the pooled effect estimates in this review were mostly consistent with those reported in the systematic review by Leong et al. (2017). The significance of *CYBA* rs4673 differed, but this may be due to differences in the current study’s focus on childhood cancer patients, thus highlighting potential pharmacogenomic distinctions between children and adults. Secondly, temporal patterns suggest that initial observations of strong associations for variants such as *RARG* rs2229774 and *SLC28A3* rs7853758 were often not detected upon subsequent replication, revealing potential false positives in the discovery studies and highlighting the “winner’s curse” phenomenon (Kraft, 2008).

While certain variants may demonstrate statistical significance, their clinical significance remains conflicted. Clinical recommendations by Aminkeng et al. (2016) suggest pharmacogenomic testing for *RARG* rs2229774, *SLC28A3* rs7853758 and *UGT1A6* rs17863783 variants in childhood cancer patients indicated for doxorubicin or daunorubicin treatment. However, since the publication of this recommendation in 2016, interest in this field of research has increased exponentially with the number of included studies published in 2020–2024 being almost equivalent to those from the 2010s. Incorporation of this growing body of evidence in the meta-analysis showed that, of the three recommended variants, only *UGT1A6* rs17863783 exhibited a significant association with ACT. Therefore, in agreement with the systematic reviews conducted by Conyers et al. (2017) and Linschoten et al. (2018), the findings of this review highlight the need for further validation of genetic associations prior to implementation of such guidelines. Meanwhile, ClinVar currently classifies all three variants as “benign” (National Center for Biotechnology Information, c.2024), indicating no clinical actionability in the general population, although this may not apply to anthracycline-treated patients (Richards et al., 2015). We highlight the conflicting clinical interpretations based on the same body of evidence and the evolving nature of this field. The varied conclusions may stem from the lack of a unified definition of ACT, different study designs and genetic approaches, variability in adjusting for confounding factors, and the polygenic nature of ACT.

The inconclusive evidence on genetic associations with ACT raises doubts about the accuracy of prediction models. Studies have shown that the addition of genetic variables increased the accuracy of clinical prediction models, with a notable AUC of 0.87 reported by Visscher et al. (2012). Yet, when this combined model was replicated by Visscher et al. (2013) using identical variables on a different cohort, its accuracy declined to an AUC of 0.67. Similarly, Chaix et al. (2020) showed that the combined model achieved an AUC of 0.9923 during training but only 0.7156 in the testing set. This inconsistency may suggest over-fitting of these models (Subramanian and Simon, 2013) or highlight important differences unexplained by the model, further necessitating validation of prediction models in other cohorts. This reinforces our concerns about the consistency of association evidence, particularly since the models in Visscher et al., 2012; Visscher et al., 2013 also included the *SLC28A3* rs7853758 variant as one of the genetic variables. We also detected publication bias in the evidence for these models, supporting the same concerns raised by Siemens et al. (2022) in their systematic review of pharmacogenomic models across different drugs.

The model of Visscher et al., 2012 was subsequently found to be highly cost-effective in comparison to usual care, aligning with the NICE guidelines (National Institute for Health and Care Excellence, 2012) which may recommend this strategy for anthracycline-treated patients. Nonetheless, as Plöthner et al. (2016) highlighted in their systematic review, the cost-effectiveness of pharmacogenomic tests depends on their sensitivity, specificity and the association between the genotype and clinical outcome. Given the inconclusive nature of upstream evidence in these two areas, we hesitate to draw conclusions regarding the cost-effectiveness of pharmacogenomic testing for ACT. Importantly, our comprehensive search strategy identified only one relevant study, highlighting an under-informed area of research. Future clinical effectiveness studies should aim to incorporate economic evaluations in their study designs or examine the costs of the pharmacogenomic tests used. Ongoing studies by the Australia and New Zealand Cardio-Oncology Registry (Lapirow et al., 2021) and also Yan et al. (2020) may help address this research gap. With decreasing sequencing costs and the adoption of multi-variant panels, pharmacogenomic tests are expected be less costly (Verbelen et al., 2017), enabling exploration of more complex genetic variations for ACT.

The strengths of this review lie in its comprehensive search strategy, robust statistical analyses and the extensive scale of variants studied. The findings provide a multi-perspective and timely overview of the current landscape of pharmacogenomic testing for ACT amidst the global drive towards pharmacogenomics. Nonetheless, the limitations of this review warrant discussion. Firstly, potential unconscious bias among reviewers may have influenced study selection, contributing to inter-rater variability (κ = 0.772–0.866). Secondly, most included studies adopted retrospective non-randomised observational study designs and adjusted for different ACT confounders (e.g., age, sex and cumulative anthracycline dose). This may have affected the summary estimates as they were pooled from individual adjusted and unadjusted ORs. Thirdly, variations in the reporting of participant age posed challenges in terms of identifying age at key time points like cancer diagnosis or ACT occurrence. Consequently, our review included paediatric cancer patients as well as childhood cancer survivors who developed ACT in adulthood, resulting in a mixed-age population. Paediatric patients may be more prone to early-onset ACT, while adult survivors are more likely to experience late-onset ACT (Volkova and Russell, 2012), which may have contributed to heterogeneity in the findings. The included studies also exhibited variations in genotype groupings and ACT definitions used. Additionally, the meta-analysed variants were investigated in few studies (n = 2–7) with small overlapping cohorts of predominantly Caucasian patients, representing longstanding challenges in pharmacogenomic research (McInnes et al., 2021) that restrict generalisability and statistical power. Collapsing the three possible genotypes into two binary groups may have increased statistical power but also the risk for type I error (Matthews et al., 2008). Our discussion focused on the meta-analysed variants with significant associations, but this does not imply their superiority over other variants (Leong et al., 2017). Finally, we did not receive sufficient data from authors for study inclusion.

Further research exploring the genetic associations with ACT and the accuracy and cost-effectiveness of pharmacogenomic testing is necessary prior to clinical implementation. Specifically, 170 out of 193 unique variants (88.1%) were assessed in only one study and lacked functional validation in pre-clinical models and paediatric oncology trials. This may diminish the benefit of these pharmacogenomic studies in driving clinical changes (Conyers et al., 2017). Therefore, hypothesis-free GWAS approaches with large sample sizes and greater ethnic diversity could be employed more widely to identify new variants (Visscher et al., 2017), with subsequent replication in prospective CGS. Promising pre-clinical models such as human-induced pluripotent stem cell-derived cardiomyocytes (hiPSC-CMs) with *in vitro* quantification of ACT (Cejas et al., 2024) have emerged as high-throughput screening methods to provide functional validation of the identified variants. Translating pharmacogenomic approaches to transcriptomics (Scott E. N. et al., 2021) and proteomics (Mörth et al., 2021) have also offered novel insights in the relationship between gene expression and ACT. With additional validation, we could more confidently apply these markers into prediction models and assess their discriminatory abilities and cost-effectiveness. Additional studies are also required to demonstrate that pharmacogenomic testing for ACT leads to meaningful improvements in downstream clinical outcomes, such as reduced ACT incidence, and improved chemotherapy adherence, survival rates and clinical decision-making. At a broader level, other factors affecting pharmacogenomics implementation such as availability of healthcare infrastructure, national regulations, data protection and ethical considerations should also be considered (Patel, 2016; Caraballo et al., 2017; Kabbani et al., 2023).

At a global level, the predominance of included studies from developed countries highlights the need for collaborative research, especially in low- and middle-income countries where ACT management is hindered by limited supportive care and pharmacogenomic resources (Klein et al., 2017; Ayati et al., 2021). Further studies in resource-limited settings are also needed to evaluate the cost-effectiveness of pre-emptive pharmacogenomic testing (Chenchula et al., 2024). To address these challenges, we support the call for an editorial or position paper advocating for adequately powered and harmonised studies, standardised protocols and international multicentre partnerships. Key examples include the Pharmacogenetics for Every Nation Initiative (Roederer et al., 2011) and the Pharmacogenomics Global Research Network’s collaboration with Human Heredity and Health in Africa (Giacomini et al., 2021). These partnerships could integrate diverse patient populations, accelerating the development of universally applicable and accessible pharmacogenomic interventions for ACT.

In conclusion, although numerous studies have reported significant associations between genetic variants and ACT, as well as the availability of moderately accurate and cost-effective prediction models, much of the evidence remains conflicting and of very low certainty. High-quality studies with larger, ethnically diverse cohorts, along with further cost-effectiveness analyses, are needed to assess the clinical and economic impact of pharmacogenomic testing for ACT before its implementation in childhood cancer patients.

## Data Availability

The original contributions presented in the study are included in the article/Supplementary Material, further inquiries can be directed to the corresponding author.
