# Supplementary material for: Clinical and cost-effectiveness of pharmacogenomic testing for anthracycline-induced cardiotoxicity in childhood cancer: a systematic review and meta-analysis
Source: Front Pharmacol. 2025 Jul 16;16:1568320. doi: 10.3389/fphar.2025.1568320 (PMC12308240; doi:10.3389/fphar.2025.1568320)
Supplement: Supplementary file 1 [file Supplementaryfile1.docx]

Supplementary Material

#
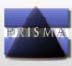
Supplementary Appendix S1. PRISMA 2020 Checklist

**PRISMA 2020 Checklist**

| **Section and Topic** | **Item #** | **Checklist item** | **Location where item is reported** |
| --- | --- | --- | --- |
| **TITLE** | | |  |
| Title | 1 | Identify the report as a systematic review. | Title |
| **ABSTRACT** | | |  |
| Abstract | 2 | See the PRISMA 2020 for Abstracts checklist. | Abstract |
| **INTRODUCTION** | | |  |
| Rationale | 3 | Describe the rationale for the review in the context of existing knowledge. | Section 1 |
| Objectives | 4 | Provide an explicit statement of the objective(s) or question(s) the review addresses. | Section 1 |
| **METHODS** | | |  |
| Eligibility criteria | 5 | Specify the inclusion and exclusion criteria for the review and how studies were grouped for the syntheses. | Section 2.1 |
| Information sources | 6 | Specify all databases, registers, websites, organisations, reference lists and other sources searched or consulted to identify studies. Specify the date when each source was last searched or consulted. | Section 2.1 |
| Search strategy | 7 | Present the full search strategies for all databases, registers and websites, including any filters and limits used. | Supplementary Appendix S2 |
| Selection process | 8 | Specify the methods used to decide whether a study met the inclusion criteria of the review, including how many reviewers screened each record and each report retrieved, whether they worked independently, and if applicable, details of automation tools used in the process. | Section 2.1 |
| Data collection process | 9 | Specify the methods used to collect data from reports, including how many reviewers collected data from each report, whether they worked independently, any processes for obtaining or confirming data from study investigators, and if applicable, details of automation tools used in the process. | Section 2.2 |
| Data items | 10a | List and define all outcomes for which data were sought. Specify whether all results that were compatible with each outcome domain in each study were sought (e.g. for all measures, time points, analyses), and if not, the methods used to decide which results to collect. | Section 2.2 |
|  | 10b | List and define all other variables for which data were sought (e.g. participant and intervention characteristics, funding sources). Describe any assumptions made about any missing or unclear information. | Section 2.2 |
| Study risk of bias assessment | 11 | Specify the methods used to assess risk of bias in the included studies, including details of the tool(s) used, how many reviewers assessed each study and whether they worked independently, and if applicable, details of automation tools used in the process. | Section 2.3 |
| Effect measures | 12 | Specify for each outcome the effect measure(s) (e.g. risk ratio, mean difference) used in the synthesis or presentation of results. | Section 2.2 |
| Synthesis methods | 13a | Describe the processes used to decide which studies were eligible for each synthesis (e.g. tabulating the study intervention characteristics and comparing against the planned groups for each synthesis (item #5)). | Section 2.2 |
|  | 13b | Describe any methods required to prepare the data for presentation or synthesis, such as handling of missing summary statistics, or data conversions. | Section 2.2 |
|  | 13c | Describe any methods used to tabulate or visually display results of individual studies and syntheses. | Section 2.2 |
|  | 13d | Describe any methods used to synthesize results and provide a rationale for the choice(s). If meta-analysis was performed, describe the model(s), method(s) to identify the presence and extent of statistical heterogeneity, and software package(s) used. | Section 2.2 |
|  | 13e | Describe any methods used to explore possible causes of heterogeneity among study results (e.g. subgroup analysis, meta-regression). | Section 2.2 |
|  | 13f | Describe any sensitivity analyses conducted to assess robustness of the synthesized results. | Section 2.2 |
| Reporting bias assessment | 14 | Describe any methods used to assess risk of bias due to missing results in a synthesis (arising from reporting biases). | Section 2.3 |
| Certainty assessment | 15 | Describe any methods used to assess certainty (or confidence) in the body of evidence for an outcome. | Section 2.3 |
| **RESULTS** | | |  |
| Study selection | 16a | Describe the results of the search and selection process, from the number of records identified in the search to the number of studies included in the review, ideally using a flow diagram. | Section 3.1 |
|  | 16b | Cite studies that might appear to meet the inclusion criteria, but which were excluded, and explain why they were excluded. | Supplementary Appendix S3 |
| Study characteristics | 17 | Cite each included study and present its characteristics. | Section 3.1 |
| Risk of bias in studies | 18 | Present assessments of risk of bias for each included study. | Section 3.2 |
| Results of individual studies | 19 | For all outcomes, present, for each study: (a) summary statistics for each group (where appropriate) and (b) an effect estimate and its precision (e.g. confidence/credible interval), ideally using structured tables or plots. | Section 3.3 |
| Results of syntheses | 20a | For each synthesis, briefly summarise the characteristics and risk of bias among contributing studies. | Sections 3.3–3.4 |
|  | 20b | Present results of all statistical syntheses conducted. If meta-analysis was done, present for each the summary estimate and its precision (e.g. confidence/credible interval) and measures of statistical heterogeneity. If comparing groups, describe the direction of the effect. | Section 3.3 |
|  | 20c | Present results of all investigations of possible causes of heterogeneity among study results. | Section 3.3 |
|  | 20d | Present results of all sensitivity analyses conducted to assess the robustness of the synthesized results. | Section 3.3 |
| Reporting biases | 21 | Present assessments of risk of bias due to missing results (arising from reporting biases) for each synthesis assessed. | Section 3.2 |
| Certainty of evidence | 22 | Present assessments of certainty (or confidence) in the body of evidence for each outcome assessed. | Sections 3.3–3.4 |
| **DISCUSSION** | | |  |
| Discussion | 23a | Provide a general interpretation of the results in the context of other evidence. | Section 4 |
|  | 23b | Discuss any limitations of the evidence included in the review. | Section 4 |
|  | 23c | Discuss any limitations of the review processes used. | Section 4 |
|  | 23d | Discuss implications of the results for practice, policy, and future research. | Section 4 |
| **OTHER INFORMATION** | | |  |
| Registration and protocol | 24a | Provide registration information for the review, including register name and registration number, or state that the review was not registered. | Section 2 |
|  | 24b | Indicate where the review protocol can be accessed, or state that a protocol was not prepared. | Section 2 |
|  | 24c | Describe and explain any amendments to information provided at registration or in the protocol. | Section 2 |
| Support | 25 | Describe sources of financial or non-financial support for the review, and the role of the funders or sponsors in the review. | Acknowledgments |
| Competing interests | 26 | Declare any competing interests of review authors. | Conflict of interest statement |
| Availability of data, code and other materials | 27 | Report which of the following are publicly available and where they can be found: template data collection forms; data extracted from included studies; data used for all analyses; analytic code; any other materials used in the review. | Supplementary Appendix S2, References |

*From:*  Page MJ, McKenzie JE, Bossuyt PM, Boutron I, Hoffmann TC, Mulrow CD, et al. The PRISMA 2020 statement: an updated guideline for reporting systematic reviews. BMJ 2021;372:n71. doi: 10.1136/bmj.n71

For more information, visit: <http://www.prisma-statement.org/>

# Supplementary Appendix S2. Search strategies

## S2.1. Search strategy for databases

**Supplementary Table S1.** Search strategy for MEDLINE(R) ALL via Ovid <1946 to 1 October 2024>.

| Line | Search terms | Results |
| --- | --- | --- |
| **Search concept 1: pharmacogenomics** | | |
| 1 | exp Pharmacogenetics/ or exp Genetic Testing/ or exp Whole Genome Sequencing/ or exp Genetic Association Studies/ or exp Polymorphism, Genetic/ or exp Genetic Variation/ | 1304149 |
| 2 | pharmacogen* or pharmaco-gen* | 28007 |
| 3 | genetic* or gene or genom* or genotype* | 5646272 |
| 4 | (genetic* or gene or genom* or genotype* or nucleotide*) adj3 (test* or screening* or sequenc* or marker* or expression* or varia* or polymorphism*) | 2205787 |
| 5 | or/1-4 | 5725913 |
| **Search concept 2: anthracycline** | | |
| 6 | exp Anthracyclines/ | 80960 |
| 7 | anthracyclin* or anthracycline antibiotics | 18908 |
| 8 | doxorubicin or doxorubic* or liposomal doxorubicin or DOX-SL or Lipo-Dox or Lipodox or Doxil or doxorubicin hydrochloride or Adriblastin* or Adriablastin* or Adriamyc* or Caelyx or Myocet | 89988 |
| 9 | daunorubicin or daunorubic* or daunomycin or dauno-rubidomyc* or dauno rubidomyc* or rubidomyc* or rubomycin or liposomal daunorubicin or Daunoxom* or daunosom* or NSC 82151 or NSC82151 or daunorubicin hydrochloride or Cerubidine or Daunoblastin* | 11362 |
| 10 | epirubicin or epirubic* or 4'-epiadriamycin or 4'-epi-adriamycin or 4'-epidoxorubicin or 4'-epi-doxorubicin or 4'-epi-DXR or IMI-28 or IMI28 or NSC 256942 or NSC256942 or epirubicin hydrochloride or Ellence or Farmorubicin* or Pharmorubicin* | 8309 |
| 11 | idarubicin or idarubic* or 4-demethoxydaunorubicin or 4-desmethoxydaunorubicin or IMI-30 or IMI30 or NSC 256439 or NSC256439 or idarubicin hydrochloride or Idamycin or Zavedos | 2642 |
| 12 | or/6-11 | 118098 |
| **Search concept 3: cardiotoxicity** | | |
| 13 | Cardiotoxicity/ | 5297 |
| 14 | cardiotox* or cardio-tox* or cardiac tox* | 25571 |
| 15 | (heart or cardiac or ventric*) adj5 (damage* or disease* or disorder* or dysfunction or failure or event* or complication*) | 721817 |
| 16 | cardiomyop* or arrhythmi* | 279461 |
| 17 | cancer therapy-related cardiovascular dysfunction or CTRCD or cancer treatment-related cardiovascular toxicity or CTR-CVT or CTRCVT or major adverse cardiac event* or major adverse cardiovascular event* or MACE | 21926 |
| 18 | or/13-17 | 917911 |
| **Search concept 4: children** | | |
| 19 | exp Pediatrics/ or exp Child/ or exp Minors/ or exp Adolescent/ | 3510501 |
| 20 | p?ediatric* or infan* or newborn* or new-born* or baby* or babies or neonat* or toddler* or child* or kid or kids or boy* or girl* | 3912972 |
| 21 | teen* or adolesc* or juvenil* or puber* or pubescen* or prepubescen* or prepuberty* or young people or young person* or youth* or minor* or underag* | 2879953 |
| 22 | (nursery or pre or primary or secondary or elementary or high) adj school* | 91799 |
| 23 | school* or student* or preschool* or highschool* or kindergar* or school age* or schoolage* or schoolchild* or school child* | 1696538 |
| 24 | or/19-23 | 5863915 |
| **Search concept 5: cancer** | | |
| 25 | exp Neoplasms/ | 4036651 |
| 26 | cancer* or tumo?r* or malignan* or carcino* or onco* or neoplas* or chemotherap* or anticancer* or anti-cancer* or antineoplas* or anti-neoplas* | 5580514 |
| 27 | leuk?emi* or childhood ALL or AML or lymphom* or hodgkin* or non hodgkin or sarcom* or Ewing* or osteosarcom* or wilms tumor or wilms* or nephroblastom* or neuroblastom* or rhabdomyosarcom* or teratom* or hepatom* or hepatoblastom* or medulloblastom* or PNET* or primitive neuroectodermal tumo?r* or retinoblastom* or meningiom* or gliom* | 1039450 |
| 28 | or/25-27 | 6023164 |
| **Final search** | | |
| **29** | **5 and 12 and 18 and 24 and 28** | **277** |

**Supplementary Table S2.** Search strategy for Embase Classic+Embase via Ovid <1947 to 1 October 2024>.

| Line | Search terms | Results |
| --- | --- | --- |
| **Search concept 1: pharmacogenomics** | | |
| 1 | exp pharmacogenetics/ or exp genetic screening/ or exp whole genome sequencing/ or exp genetic association study/ or exp genetic polymorphism/ or exp genetic variation/ | 768907 |
| 2 | pharmacogen* or pharmaco-gen* | 49613 |
| 3 | genetic* or gene or genom* or genotype* | 6575197 |
| 4 | (genetic* or gene or genom* or genotype* or nucleotide*) adj3 (test* or screening* or sequenc* or marker* or expression* or varia* or polymorphism*) | 3328004 |
| 5 | or/1-4 | 6722267 |
| **Search concept 2: anthracycline** | | |
| 6 | exp anthracycline/ | 29393 |
| 7 | anthracyclin* or anthracycline antibiotics | 48760 |
| 8 | doxorubicin or doxorubic* or liposomal doxorubicin or DOX-SL or Lipo-Dox or Lipodox or Doxil or doxorubicin hydrochloride or Adriblastin* or Adriablastin* or Adriamyc* or Caelyx or Myocet | 247616 |
| 9 | daunorubicin or daunorubic* or daunomycin or dauno-rubidomyc* or dauno rubidomyc* or rubidomyc* or rubomycin or liposomal daunorubicin or Daunoxom* or daunosom* or NSC 82151 or NSC82151 or daunorubicin hydrochloride or Cerubidine or Daunoblastin* | 36053 |
| 10 | epirubicin or epirubic* or 4'-epiadriamycin or 4'-epi-adriamycin or 4'-epidoxorubicin or 4'-epi-doxorubicin or 4'-epi-DXR or IMI-28 or IMI28 or NSC 256942 or NSC256942 or epirubicin hydrochloride or Ellence or Farmorubicin* or Pharmorubicin* | 35242 |
| 11 | idarubicin or idarubic* or 4-demethoxydaunorubicin or 4-desmethoxydaunorubicin or IMI-30 or IMI30 or NSC 256439 or NSC256439 or idarubicin hydrochloride or Idamycin or Zavedos | 13656 |
| 12 | or/6-11 | 321611 |
| **Search concept 3: cardiotoxicity** | | |
| 13 | exp cardiotoxicity/ | 56377 |
| 14 | cardiotox* or cardio-tox* or cardiac tox* | 67681 |
| 15 | (heart or cardiac or ventric*) adj5 (damage* or disease* or disorder* or dysfunction or failure or event* or complication*) | 1277250 |
| 16 | cardiomyop* or arrhythmi* | 469003 |
| 17 | cancer therapy-related cardiovascular dysfunction or CTRCD or cancer treatment-related cardiovascular toxicity or CTR-CVT or CTRCVT or major adverse cardiac event* or major adverse cardiovascular event* or MACE | 47199 |
| 18 | or/13-17 | 1591818 |
| **Search concept 4: children** | | |
| 19 | exp pediatrics/ or exp child/ or exp "minor (person)"/ or exp adolescent/ | 4697181 |
| 20 | p?ediatric* or infan* or newborn* or new-born* or baby* or babies or neonat* or toddler* or child* or kid or kids or boy* or girl* | 4993482 |
| 21 | teen* or adolesc* or juvenil* or puber* or pubescen* or prepubescen* or prepuberty* or young people or young person* or youth* or minor* or underag* | 2872203 |
| 22 | (nursery or pre or primary or secondary or elementary or high) adj school* | 126368 |
| 23 | school* or student* or preschool* or highschool* or kindergar* or school age* or schoolage* or schoolchild* or school child* | 2024086 |
| 24 | or/19-23 | 7070671 |
| **Search concept 5: cancer** | | |
| 25 | exp neoplasm/ | 6313182 |
| 26 | cancer* or tumo?r* or malignan* or carcino* or onco* or neoplas* or chemotherap* or anticancer* or anti-cancer* or antineoplas* or anti-neoplas* | 7856706 |
| 27 | leuk?emi* or childhood ALL or AML or lymphom* or hodgkin* or non hodgkin or sarcom* or Ewing* or osteosarcom* or wilms tumor or wilms* or nephroblastom* or neuroblastom* or rhabdomyosarcom* or teratom* or hepatom* or hepatoblastom* or medulloblastom* or PNET* or primitive neuroectodermal tumo?r* or retinoblastom* or meningiom* or gliom* | 1630606 |
| 28 | or/25-27 | 8719491 |
| **Final search** | | |
| **29** | **5 and 12 and 18 and 24 and 28** | **851** |

**Supplementary Table S3.** Search strategy for CENTRAL via Cochrane Library <2000 to 1 October 2024>.

| Line | Search terms | Results |
| --- | --- | --- |
| **Search concept 1: pharmacogenomics** | | |
| 1 | (pharmacogen* or pharmaco-gen* or genetic* or gene or genom* or genotype* or nucleotide* or polymorphism*):ti,ab,kw | 90973 |
| **Search concept 2: anthracycline** | | |
| 2 | (anthracyclin* or doxorubic* or daunorubic* or epirubic* or idarubic*):ti,ab,kw | 15922 |
| **Search concept 3: cardiotoxicity** | | |
| 3 | (cardiotox* or cardio-tox* or cardiac tox* or cardiomyop* or arrhythmi* or heart failure* or ventricular dysfunction):ti,ab,kw | 67289 |
| **Search concept 4: children** | | |
| 4 | (pediatric* or paediatric* or infan* or newborn* or new-born* or baby* or babies or neonat* or toddler* or child* or kid or kids or boy* or girl* or teen* or adolesc* or juvenil* or puber* or pubescen* or prepubescen* or prepuberty* or young people or young person* or youth* or minor* or underag* or school* or student* or preschool* or high school* or kindergar* or school age* or schoolage* or schoolchild* or school child*):ti,ab,kw | 461798 |
| **Search concept 5: cancer** | | |
| 5 | (cancer* or tumor* or tumour* or malignan* or carcino* or onco* or neoplas* or chemotherap* or anticancer* or anti-cancer* or antineoplas* or anti-neoplas* or leukemi* or leukaemi* or childhood ALL or AML or lymphom* or hodgkin* or non hodgkin or sarcom* or Ewing* or osteosarcom* or wilms tumor or wilms* or nephroblastom* or neuroblastom* or rhabdomyosarcom* or teratom* or hepatom* or hepatoblastom* or medulloblastom* or PNET* or primitive neuroectodermal tumor* or primitive neuroectodermal tumour* or retinoblastom* or meningiom* or gliom*):ti,ab,kw | 320708 |
| **Final search** | | |
| **6** | **1 and 2 and 3 and 4 and 5** | **34** |

**Supplementary Table S4.** Search strategy for Scopus via scopus.com <1966 to 1 October 2024>.

| Line | Search terms | Results |
| --- | --- | --- |
| **Search concept 1: pharmacogenomics** | | |
| 1 | TITLE-ABS-KEY ( pharmacogen* OR pharmaco-gen* ) | 46,514 |
| 2 | TITLE-ABS-KEY ( genetic* OR gene OR genom* OR genotype* ) | 7,209,705 |
| 3 | TITLE-ABS-KEY ( ( genetic* OR gene OR genom* OR genotype* OR nucleotide* ) W/3 ( test* OR screening* OR sequenc* OR marker* OR expression* OR varia* OR polymorphism* ) ) | 3,518,689 |
| 4 | #1 OR #2 OR #3 | 7,296,174 |
| **Search concept 2: anthracycline** | | |
| 5 | TITLE-ABS-KEY ( anthracyclin* OR "anthracycline antibiotics" ) | 39,246 |
| 6 | TITLE-ABS-KEY ( doxorubicin OR doxorubic* OR "liposomal doxorubicin" OR dox-sl OR lipo-dox OR lipodox OR doxil OR "doxorubicin hydrochloride" OR adriblastin* OR adriablastin* OR adriamyc* OR caelyx OR myocet ) | 232,345 |
| 7 | TITLE-ABS-KEY ( daunorubicin OR daunorubic* OR daunomycin OR dauno-rubidomyc* OR "dauno rubidomyc*" OR rubidomyc* OR rubomycin OR "liposomal daunorubicin" OR daunoxom* OR daunosom* OR "nsc 82151" OR nsc82151 OR "daunorubicin hydrochloride" OR cerubidine OR daunoblastin* ) | 33,766 |
| 8 | TITLE-ABS-KEY ( epirubicin OR epirubic* OR 4'-epiadriamycin OR 4'-epi-adriamycin OR 4'-epidoxorubicin OR 4'-epi-doxorubicin OR 4'-epi-dxr OR imi-28 OR imi28 OR "nsc 256942" OR nsc256942 OR "epirubicin hydrochloride" OR ellence OR farmorubicin* OR pharmorubicin* ) | 255 |
| 9 | TITLE-ABS-KEY ( idarubicin OR idarubic* OR 4-demethoxydaunorubicin OR 4-desmethoxydaunorubicin OR imi-30 OR imi30 OR "nsc 256439" OR nsc256439 OR "idarubicin hydrochloride" OR idamycin OR zavedos ) | 11,970 |
| 10 | CASREGNUMBER ( 23214-92-8 OR 20830-81-3 OR 56390-09-1 OR 58957-92-9 ) | 241,890 |
| 11 | #5 OR #6 OR #7 OR #8 OR #9 OR #10 | 291,651 |
| **Search concept 3: cardiotoxicity** | | |
| 12 | TITLE-ABS-KEY ( cardiotox* OR cardio-tox* OR "cardiac tox*" ) | 59,264 |
| 13 | TITLE-ABS-KEY ( ( heart OR cardiac OR ventric* ) W/5 ( damage* OR disease* OR disorder* OR dysfunction OR failure OR event* OR complication* ) ) | 1,057,557 |
| 14 | TITLE-ABS-KEY ( cardiomyop* OR arrhythmi* ) | 409,300 |
| 15 | TITLE-ABS-KEY ( "cancer therapy-related cardiovascular dysfunction" OR ctrcd OR "cancer treatment-related cardiovascular toxicity" OR ctr-cvt OR ctrcvt OR "major adverse cardiac event*" OR "major adverse cardiovascular event*" OR mace ) | 29,554 |
| 16 | #12 OR #13 OR #14 OR #15 | 1,339,233 |
| **Search concept 4: children** | | |
| 17 | TITLE-ABS-KEY ( pediatric* OR paediatric* OR infan* OR newborn* OR new-born* OR baby* OR babies OR neonat* OR toddler* OR child* OR kid OR kids OR boy* OR girl* ) | 5,379,945 |
| 18 | TITLE-ABS-KEY ( teen* OR adolesc* OR juvenil* OR puber* OR pubescen* OR prepubescen* OR prepuberty* OR "young people" OR "young person*" OR youth* OR minor* OR underag* ) | 3,960,243 |
| 19 | TITLE-ABS-KEY ( ( nursery OR pre OR primary OR secondary OR elementary OR high ) W/1 school* ) | 334,958 |
| 20 | TITLE-ABS-KEY ( school* OR student* OR preschool* OR highschool* OR kindergar* OR "school age*" OR schoolage* OR schoolchild* OR "school child*" ) | 3,751,590 |
| 21 | #17 OR #18 OR #19 OR #20 | 9,520,480 |
| **Search concept 5: cancer** | | |
| 22 | TITLE-ABS-KEY ( cancer* OR tumor* OR tumour* OR malignan* OR carcino* OR onco* OR neoplas* OR chemotherap* OR anticancer* OR anti-cancer* OR antineoplas* OR anti-neoplas* ) | 7,183,713 |
| 23 | TITLE-ABS-KEY ( leukemi* OR leukaemi* OR "childhood all" OR aml OR lymphom* OR hodgkin* OR "non hodgkin" OR sarcom* OR ewing* OR osteosarcom* OR "wilms tumor" OR wilms* OR nephroblastom* OR neuroblastom* OR rhabdomyosarcom* OR teratom* OR hepatom* OR hepatoblastom* OR medulloblastom* OR pnet* OR "primitive neuroectodermal tumor*" OR "primitive neuroectodermal tumour*" OR retinoblastom* OR meningiom* OR gliom* ) | 1,386,410 |
| 24 | #22 OR #23 | 7,508,795 |
| **Final search** | | |
| **25** | **#4 AND #11 AND #16 AND #21 AND #24** | **770** |

**Supplementary Table S5.** Search strategy for CINAHL Plus via EBSCOhost <1937 to 1 October 2024>.

| Line | Search terms | Results |
| --- | --- | --- |
| **Search concept 1: pharmacogenomics** | | |
| 1 | (MH "Pharmacogenetics") OR (MH "Genetic Screening+") OR (MH "Genome Wide Association Study") OR (MH "Polymorphism, Genetic+") OR (MH "Genetic Variation+") | 103,813 |
| 2 | pharmacogen* or pharmaco-gen* | 4,105 |
| 3 | genetic* or gene or genom* or genotype* | 357,065 |
| 4 | (genetic* or gene or genom* or genotype* or nucleotide*) N3 (test* or screening* or sequenc* or marker* or expression* or varia* or polymorphism*) | 156,530 |
| 5 | S1 OR S2 OR S3 OR S4 | 375,132 |
| **Search concept 2: anthracycline** | | |
| 6 | (MH "Anthracyclines+") | 7,685 |
| 7 | anthracyclin* or anthracycline antibiotics | 3,640 |
| 8 | doxorubicin or doxorubic* or liposomal doxorubicin or DOX-SL or Lipo-Dox or Lipodox or Doxil or doxorubicin hydrochloride or Adriblastin* or Adriablastin* or Adriamyc* or Caelyx or Myocet | 8,846 |
| 9 | daunorubicin or daunorubic* or daunomycin or dauno-rubidomyc* or dauno rubidomyc* or rubidomyc* or rubomycin or liposomal daunorubicin or Daunoxom* or daunosom* or NSC 82151 or NSC82151 or daunorubicin hydrochloride or Cerubidine or Daunoblastin* | 528 |
| 10 | epirubicin or epirubic* or 4'-epiadriamycin or 4'-epi-adriamycin or 4'-epidoxorubicin or 4'-epi-doxorubicin or 4'-epi-DXR or IMI-28 or IMI28 or NSC 256942 or NSC256942 or epirubicin hydrochloride or Ellence or Farmorubicin* or Pharmorubicin* | 1,121 |
| 11 | idarubicin or idarubic* or 4-demethoxydaunorubicin or 4-desmethoxydaunorubicin or IMI-30 or IMI30 or NSC 256439 or NSC256439 or idarubicin hydrochloride or Idamycin or Zavedos | 278 |
| 12 | S6 OR S7 OR S8 OR S9 OR S10 OR S11 | 13,029 |
| **Search concept 3: cardiotoxicity** | | |
| 13 | (MH "Cardiotoxicity") | 1,711 |
| 14 | cardiotox* or cardio-tox* or cardiac tox* | 4,436 |
| 15 | (heart or cardiac or ventric*) N5 (damage* or disease* or disorder* or dysfunction or failure or event* or complication*) | 186,624 |
| 16 | cardiomyop* or arrhythmi* | 53,811 |
| 17 | cancer therapy-related cardiovascular dysfunction or CTRCD or cancer treatment-related cardiovascular toxicity or CTR-CVT or CTRCVT or major adverse cardiac event* or major adverse cardiovascular event* or MACE | 6,777 |
| 18 | S13 OR S14 OR S15 OR S16 OR S17 | 227,057 |
| **Search concept 4: children** | | |
| 19 | (MH "Pediatrics+") OR (MH "Child+") OR (MH "Minors (Legal)") OR (MH "Adolescence+") | 1,151,714 |
| 20 | p#ediatric* or infan* or newborn* or new-born* or baby* or babies or neonat* or toddler* or child* or kid or kids or boy* or girl* | 1,221,160 |
| 21 | teen* or adolesc* or juvenil* or puber* or pubescen* or prepubescen* or prepuberty* or young people or young person* or youth* or minor* or underag* | 777,212 |
| 22 | (nursery or pre or primary or secondary or elementary or high) N1 school* | 57,103 |
| 23 | school* or student* or preschool* or highschool* or kindergar* or school age* or schoolage* or schoolchild* or school child* | 640,262 |
| 24 | S19 OR S20 OR S21 OR S22 OR S23 | 1,880,347 |
| **Search concept 5: cancer** | | |
| 25 | (MH "Neoplasms+") | 676,289 |
| 26 | cancer* or tumo#r* or malignan* or carcino* or onco* or neoplas* or chemotherap* or anticancer* or anti-cancer* or antineoplas* or anti-neoplas* | 956,374 |
| 27 | leuk#emi* or childhood ALL or AML or lymphom* or hodgkin* or non hodgkin or sarcom* or Ewing* or osteosarcom* or wilms tumor or wilms* or nephroblastom* or neuroblastom* or rhabdomyosarcom* or teratom* or hepatom* or hepatoblastom* or medulloblastom* or PNET* or primitive neuroectodermal tumo#r* or retinoblastom* or meningiom* or gliom* | 123,977 |
| 28 | S25 OR S26 OR S27 | 1,026,946 |
| **Final search** | | |
| **29** | **S5 AND S12 AND S18 AND S24 AND S28** | **51** |

**Supplementary Table S6.** Search strategy for ProQuest Dissertations & Theses Global (PQDT) via ProQuest <1637 to 1 October 2024>.

| Line | Search terms | Results |
| --- | --- | --- |
| **Search concept 1: pharmacogenomics** | | |
| 1 | noft(pharmacogen* OR "pharmaco-gen*") | 1,272 |
| 2 | noft(genetic* OR gene OR genom* OR genotype*) | 358,064 |
| 3 | noft((genetic* OR gene OR genom* OR genotype* OR nucleotide*) NEAR/3 (test* OR screening* OR sequenc* OR marker* OR expression* OR varia* OR polymorphism*)) | 131,893 |
| 4 | [S1] OR [S2] OR [S3] | 359,371 |
| **Search concept 2: anthracycline** | | |
| 5 | noft(anthracyclin* OR "anthracycline antibiotics") | 728 |
| 6 | noft(doxorubicin OR doxorubic* OR "liposomal doxorubicin" OR "dox-sl" OR "lipo-dox" OR lipodox OR doxil OR "doxorubicin hydrochloride" OR adriblastin* OR adriablastin* OR adriamyc* OR caelyx OR myocet) | 3,224 |
| 7 | noft(daunorubicin OR daunorubic* OR daunomycin OR "dauno-rubidomyc*" OR "dauno rubidomyc*" OR rubidomyc* OR rubomycin OR "liposomal daunorubicin" OR daunoxom* OR daunosom* OR "nsc 82151" OR nsc82151 OR "daunorubicin hydrochloride" OR cerubidine OR daunoblastin*) | 400 |
| 8 | noft(epirubicin OR epirubic* OR "4'-epiadriamycin" OR "4'-epi-adriamycin" OR "4'-epidoxorubicin" OR "4'-epi-doxorubicin" OR "4'-epi-dxr" OR "imi-28" OR imi28 OR "nsc 256942" OR nsc256942 OR "epirubicin hydrochloride" OR ellence OR farmorubicin* OR pharmorubicin*) | 158 |
| 9 | noft(idarubicin OR idarubic* OR "4-demethoxydaunorubicin" OR "4-desmethoxydaunorubicin" OR "imi-30" OR imi30 OR "nsc 256439" OR nsc256439 OR "idarubicin hydrochloride" OR idamycin OR zavedos) | 44 |
| 10 | [S5] OR [S6] OR [S7] OR [S8] OR [S9] | 3,960 |
| **Search concept 3: cardiotoxicity** | | |
| 11 | noft(cardiotox* OR "cardio-tox*" OR "cardiac tox*" ) | 1,040 |
| 12 | noft((heart OR cardiac OR ventric*) NEAR/5 (damage* OR disease* OR disorder* OR dysfunction OR failure OR event* OR complication*)) | 23,911 |
| 13 | noft(cardiomyop* OR arrhythmi*) | 8,086 |
| 14 | noft("cancer therapy-related cardiovascular dysfunction" OR ctrcd OR "cancer treatment-related cardiovascular toxicity" OR "ctr-cvt" OR ctrcvt OR "major adverse cardiac event*" OR "major adverse cardiovascular event*" OR mace) | 947 |
| 15 | [S11] OR [S12] OR [S13] OR [S14] | 30,217 |
| **Search concept 4: children** | | |
| 16 | noft(pediatric* OR paediatric* OR infan* OR newborn* OR "new-born*" OR baby* OR babies OR neonat* OR toddler* OR child* OR kid OR kids OR boy* OR girl*) | 458,312 |
| 17 | noft(teen* OR adolesc* OR juvenil* OR puber* OR pubescen* OR prepubescen* OR prepuberty* OR "young people" OR "young person*" OR youth* OR minor* OR underag*) | 279,351 |
| 18 | noft((nursery OR "pre" OR primary OR secondary OR elementary OR high) NEAR/1 school*) | 193,083 |
| 19 | noft(school* OR student* OR preschool* OR highschool* OR kindergar* OR "school age*" OR schoolage* OR schoolchild* OR "school child*") | 909,263 |
| 20 | [S16] OR [S17] OR [S18] OR [S19] | 1,318,220 |
| **Search concept 5: cancer** | | |
| 21 | noft(cancer* OR tumor* OR tumour* OR malignan* OR carcino* OR onco* OR neoplas* OR chemotherap* OR anticancer* OR "anti-cancer*" OR antineoplas* OR "anti-neoplas*") | 185,706 |
| 22 | noft(leukemi* OR leukaemi* OR "childhood all" OR aml OR lymphom* OR hodgkin* OR "non hodgkin" OR sarcom* OR ewing* OR osteosarcom* OR "wilms tumor" OR wilms* OR nephroblastom* OR neuroblastom* OR rhabdomyosarcom* OR teratom* OR hepatom* OR hepatoblastom* OR medulloblastom* OR pnet* OR "primitive neuroectodermal tumor*" OR "primitive neuroectodermal tumour*" OR retinoblastom* OR meningiom* OR gliom*) | 35,901 |
| 23 | [S21] OR [S22] | 200,304 |
| **Final search** | | |
| **24** | **[S4] AND [S10] AND [S15] AND [S20] AND [S23]** | **26** |

**Supplementary Table S7.** Search strategy for Pharmacogenomics Knowledge Base (PharmGKB) via pharmgkb.org <2001 to 1 October 2024>.

| Line | Search terms | Results |
| --- | --- | --- |
| **Final search (under “Literature” page)** | | |
| 1 | anthracyclines and related substances AND cardiotoxicity | 29 |

**Supplementary Table S8.** Search strategy for NHS Economic Evaluation Database (EED) via crd.york.ac.uk/CRDWeb/ <1968 to 31 December 2014*>.

| Line | Search terms | Results |
| --- | --- | --- |
| **Final search** | | |
| 1 | anthracycline | 28 |

*NHS EED is no longer actively updated; their coverage ended on 31 December 2014.

**Supplementary Table S9.** Search strategy for Cost-Effectiveness Analysis (CEA) Registry via cear.tuftsmedicalcenter.org <1976 to 1 October 2024>.

| Line | Search terms | Results |
| --- | --- | --- |
| **Final search** | | |
| 1 | anthracycline | 19 |

## S2.2. Search strategy for trial registers

**Supplementary Table S10.** Search strategy for ClinicalTrials.gov via clinicaltrials.gov <2000 to 1 October 2024>.

| Line | Search terms | Results |
| --- | --- | --- |
| **Final search** | | |
| 1 | Anthracycline [Intervention/treatment] AND Cardiotoxicity [Condition/disease] AND Child [Other terms] | 16 |

**Supplementary Table S11.** Search strategy for World Health Organization International Clinical Trials Registry Platform (WHO ICTRP) via trialsearch.who.int <2005 to 1 October 2024>.

| Line | Search terms | Results |
| --- | --- | --- |
| **Final search (Basic Search interface)** | | |
| 1 | anthracycline AND cardiotoxicity | 68 |

**Supplementary Table S12.** Search strategy for International Standard Randomised Controlled Trial Number (ISRCTN) Registry via isrctn.com <2000 to 1 October 2024>.

| Line | Search terms | Results |
| --- | --- | --- |
| **Final search (Basic Search interface)** | | |
| 1 | anthracycline AND cardiotoxicity AND child | 3 |

## S2.3. Search strategy for organisations

**Supplementary Table S13.** Search strategy for Clinical Pharmacogenetics Implementation Consortium (CPIC) via cpicpgx.org/genes-drugs/ <2009 to 1 October 2024>.

| Line | Search terms | Results |
| --- | --- | --- |
| **Final search** | | |
| 1 | rubicin | 1 |

**Supplementary Table S14.** Search strategy for Canadian Pharmacogenomics Network for Drug Safety (CPNDS) via cpnds.ubc.ca/publications/ <2005 to 1 October 2024>.

| Line | Search terms | Results |
| --- | --- | --- |
| **Final search** | | |
| 1 | Section “Anthracycline-induced heart failure in the treatment of cancer” | 14 |

**Supplementary Table S15.** Search strategy for Australian Cardio-Oncology Registry (ACOR) via acor-registry.org.au/publications/ <2016 to 1 October 2024>.

| Line | Search terms | Results |
| --- | --- | --- |
| **Final search** | | |
| 1 | Sections “2016” to “2023” | 29 |

## S2.4. Search strategy for conference abstracts

**Supplementary Table S16.** Search strategy for International Society for Paediatric Oncology (SIOP) via onlinelibrary.wiley.com/loi/15455017 <2018 to 2023>.

| Conference | Search terms | Results |
| --- | --- | --- |
| **Final search** | | |
| 55th Congress of the International Society of Paediatric Oncology; 11-14 October 2023; Ottawa, Canada | anthracycline AND cardiotoxicity | 4 |
| 54th Congress of the International Society of Paediatric Oncology; 28 September - 1 October 2022; Barcelona, Spain |  | 4 |
| 53rd Congress of the International Society of Paediatric Oncology; 21-24 October 2021; Virtual |  | 3 |
| 52nd Congress of the International Society of Paediatric Oncology, 14-17 October 2020; Virtual |  | 7 |
| 51st Congress of the International Society of Paediatric Oncology; 23-26 October 2019; Lyon, France |  | 3 |
| 50th Congress of the International Society of Paediatric Oncology; 16-19 November 2018; Kyoto, Japan |  | 5 |

**Supplementary Table S17.** Search strategy for American Society of Clinical Oncology (ASCO) via meetings.asco.org/abstracts-presentations <2018 to 2023>.

| Conference | Search terms | Results |
| --- | --- | --- |
| **Final search** | | |
| 59th Annual Meeting of the American Society of Clinical Oncology; 2-6 June 2023; Chicago, Illinois | anthracycline AND cardiotoxicity | 5 |
| 58th Annual Meeting of the American Society of Clinical Oncology; 3-7 June 2022; Chicago, Illinois |  | 8 |
| 57th Annual Meeting of the American Society of Clinical Oncology; 4-8 June 2021; Virtual |  | 4 |
| 56th Annual Meeting of the American Society of Clinical Oncology; 29-31 May 2020; Virtual |  | 6 |
| 55th Annual Meeting of the American Society of Clinical Oncology; 31 May - 4 June, 2019; Chicago, Illinois |  | 4 |
| 54th Annual Meeting of the American Society of Clinical Oncology; 1-5 June 2018; Chicago, Illinois |  | 6 |

**Supplementary Table S18.** Search strategy for American Society of Pediatric Hematology/Oncology (ASPHO) via aspho.org/meetings/conference/meetings-archive <2018 to 2023>.

| Conference | Search terms | Results |
| --- | --- | --- |
| **Final search** | | |
| 2023 ASPHO Conference; 10-13 May 2023; Fort Worth, Texas | anthracycline AND cardiotoxicity | 0 |
| 2022 ASPHO Conference; 4-7 May 2022; Pittsburgh, Pennsylvania |  | 0 |
| 2021 ASPHO Conference; 20-23 April 2021; Virtual |  | 1 |
| 2020 ASPHO Conference; 6-9 May 2020; Fort Worth, Texas |  | 0 |
| 2019 ASPHO Conference; 1-4 May 2019; New Orleans, Louisiana |  | 2 |
| 2018 ASPHO Conference; 2-5 May 2018; Pittsburgh, Pennsylvania |  | 0 |

## S2.5. Citation searching

**Supplementary Table S19.** Search strategy for reference lists of relevant systematic reviews via Web of Science Core Collection <1900 to 1 October 2024>.

| Study | Results from Web of Science | Results after de-duplication |
| --- | --- | --- |
| **Final search** | | |
| Aminkeng et al., 2016 | 111 | **111** |
| Conyers et al., 2017 | 86 | **77** |
| Ehrhardt et al., 2023 | 95 | **86** |
| Hurkmans et al., 2022 | 105 | **86** |
| Leong et al., 2017 | 58 | **31** |
| Linschoten et al., 2018 | 79 | **43** |

# Supplementary Appendix S3. Characteristics of studies

**Supplementary Table S20.** Characteristics of included clinical effectiveness and cost-effectiveness studies.

| **Study design** | | **Population** | | | | | | | **Exposure** | | | | **Outcome** |
| --- | --- | --- | --- | --- | --- | --- | --- | --- | --- | --- | --- | --- | --- |
| **Study** | **Study design** | **Cohort size (n); cohort name** | **Country;**  **Ethnicity** | **Sex (n, male/female)** | **Age at cancer diagnosis (years)** | **Length of follow-up (years)** | **Cancer type** | **Anthracycline type; Cumulative dose (mg/m²)** | **Genotyping technique** | **Biospecimen type;**  **Quality control (call rate, HWE)** | **Chest radiation (n received)** | **Cardio-protectant use (n used)** | **Definition of ACT**  **(produces binary outcomes unless otherwise specified)** |
| **Clinical effectiveness studies** | | | | | | | | | | | | | |
| Aminkeng et al., 2015 | Case-control  GWAS and CGS | *Discovery:*  Cases, 32  Controls, 248  *Replication 1:*  Cases, 22  Controls, 74  *Replication 2:*  Cases, 19  Controls, 61  CPNDS | Canada;  *Discovery:*  **Canadian European**  *Replication 1:* **Dutch European**  *Replication 2:*  **East Asian**, Hispanic USA, African, Aboriginal Canadian | *Discovery:*  Cases, 15/17  Controls, 136/112  *Replication 1:*  Cases, 12/10  Controls, 38/36  *Replication 2:*  Cases, 9/10  Controls, 31/30 | *Discovery:*  Cases, 9 (IQR 2.5–14) Controls, 4 (IQR 2–7.5)  *Replication 1:*  Cases, 7.5 (IQR 5–12)  Controls, 11 (IQR 6–14)  *Replication 2:*  Cases, Hispanic USA 14 (IQR 12.5–17.5); African 4.5 (IQR 4–5); East Asian 3.5 (IQR 0.5–8); Aboriginal Canadians 3.5 (IQR 1.5–5.5)  Controls, Hispanic USA 5.5 (IQR 3–12); African 4 (IQR 1.5–7); East Asian 6 (IQR 2.5–9.5); Aboriginal Canadians 4 (IQR 2.5–7)  *^Age at start of treatment* | *Discovery:*  Cases, 7.5 (range 2.5–15.5)  Controls, 9 (range 7–12)  *Replication 1:*  Cases, 22 (range 19–25)  Controls, 17 (range 14–22)  *Replication 2:*  Cases, range 2–19  Controls, range 5–10.5 | **ALL**, AML, other leukaemia, HL, NHL, OS, RMS, Ewing sarcoma, other sarcoma, NB, HB, WT | Doxo, Dauno, Epi  *Discovery:*  Cases, 260 (IQR 177.5–365)  Controls, 175 (IQR 140–295)  *Replication 1:*  Cases, 407.5 (IQR 270–480)  Controls, 277.5 (IQR 180–364)  *Replication 2:*  Cases, Hispanic USA 200 (IQR 141–245); African 319.5 (IQR 240–399); East Asian 300.5 (IQR 270–362.5); Aboriginal Canadians 250 (IQR 137.5–330)  Controls, Hispanic USA 162.5 (IQR 150–300); African 240 (IQR 114–382.5); East Asian 290 (IQR 162.5–360); Aboriginal Canadians 150 (IQR 135–245)  In doxorubicin isotoxic equivalent doses | Customised Illumina Infinium  HumanOmniExpress assay  (microarray) | Biospecimen type NR;  Call rate = 99.5%,  HWE P>10^-4^ | *Discovery:*  Cases, 12  Controls, 40  *Replication 1:*  Cases, 6  Controls, 18  *Replication 2:*  Cases, 1  Controls, 2 | Cardio-protectant type NR  *Discovery:*  Cases, 12  Controls, 40  *Replication 1:*  Cases, 6  Controls, 18  *Replication 2:*  Cases, 1  Controls, 2 | i) FS ≤24%; or  ii) Signs/symptoms requiring intervention according to CTCAE Version 3 |
| Aslam et al., 2021 | Cross-sectional  CGS | Cases, 79  Controls, 137 | Pakistan;  **Pakistani** | NR separately for cardiotoxicity arm | NR separately for cardiotoxicity arm | 15 days after remission induction | **ALL** | Dauno;  Cumulative dose NR | Tetra-primer ARMS-PCR and gel electrophoresis  (PCR-based) | Blood;  Quality control NR | NR | NR | i) Any drop in LVEF > 10 to < 53%; and  ii) Presence of pericardial effusion, high muscular restrictive VSD, spontaneous closure of ASD, regressed pulmonary hypertension or tamponade |
| Blanco et al., 2008 | Nested case-control  CGS | Cases, 30  Controls, 115  CCSS | USA;  **White**, Black | Cases, 10/20  Controls, 57/58 | Cases, 10.3 ± 6.5  Controls, 9.1 ± 5.8 | NR, “controls were followed at least until the case developed the event” | **Leukaemia**, HL, NHL, bone cancer, STS | Anthracycline type NR;  <100: 1 case, 2 controls  100–350: 13 cases, 46 controls  350–500: 7 cases, 31 controls  >500: 9 cases, 36 controls | i) PCR-RFLP,  ii) allelic discrimination with specific fluorescent probes  (PCR-based) | Buccal cells/saliva;  Call rate NR,  HWE P>0.05 | Cases, 18  Controls, 63 | NR | Self-reported signs/symptoms of CHF and medications used for CHF management |
| Blanco et al., 2012 | Case-control  CGS | Cases, 170  Controls, 317  COG-ALTE03N1 | USA;  **Non-Hispanic White**, Hispanic, Black | Cases, 76/94  Controls, 162/155 | Cases, 7.3 (range 0–20.7)  Controls, 7.6 (range 0–21.1) | Cases, 7 (range 0.1–35.1)  Controls, 11.2 (range 0.4–40.3) | **ALL**, AML, HL, NHL, bone tumour, STS | Doxo, Dauno, Epi, Ida;  Cases, 300 (range 0–575)  Controls, 140 (range 0–1050)  In doxorubicin isotoxic equivalent doses | Allelic discrimination with specific fluorescent probes  (PCR-based) | Blood, buccal cells/saliva  Call rate NR,  HWE P>0.05 | Cases, 42  Controls, 43 | NR | i) Signs/symptoms of cardiac compromise (dyspnoea, orthopnoea, fatigue, oedema, hepatomegaly, and/or rales) according to  AHA criteria; or  ii) LVEF ≤40%, FS ≤28% if asymptomatic |
| Boies, 2021 | Case-control GWAS  [Thesis] | Cases, 259  Controls, 879  MD Anderson Cancer Centre | USA;  **Non-Hispanic White**, Hispanic, Non-Hispanic Black, Asian | Cases, 140/119  Controls, 475/404 | Cases, 6.06 ± 5.27  Controls, 7.42 ± 5.28 | Cases, 14.47 ± 9.97  Controls, 12.25 ± 7.04 | **Leukaemia**, sarcoma, lymphoma, WT, NB | Doxo;  Cases, 224.31 ± 158.62  Controls, 197 ± 126.96 | NR | NR | Cases, 19  Controls, 51 | NR | i) Presence of a cardiovascular condition (heart failure, coronary heart disease, myocardial infarction, ischemic heart disease, congestive heart failure, pericarditis, myocarditis, pericardial effusion, valvular heart disease, arrhythmia, angina pectoris, cardiomyopathy, cardiac transplant, structural abnormalities to the heart, and/or utilization of a ventricular assist device. Also patients treated with cardiac medications.); and/or  ii) LVEF ≤45%, FS ≤25% in two measurements |
| Chaix et al., 2020 | Nested case-control  GWAS | *Discovery:*  Cases, 183  Controls, 106  *Replication:*  Cases, 30  Controls, 30 | Canada;  Ethnicity NR | *Discovery:*  Cases, 91/92  Controls, 49/57  *Replication:*  Cases, 13/17  Controls, 18/12 | *Discovery:*  Cases, 4 (IQR 2–7)  Controls, 6 (IQR 2–10)  *Replication:*  Cases, 4 (IQR 2–7)  Controls, 3 (IQR 1.8–6)  *^Age at start of treatment* | *Discovery:*  Cases, 9 (IQR 6–12.3)  Controls, 8.5 (IQR 5–12.3)  *Replication:*  Cases, 12 (IQR 8.3–15.8)  Controls, 10.5 (IQR 6.8–14.3) | **Leukaemia (ALL, AML),**  OS, RMS, Ewing, NB, HB, HL, NHL, WT | Doxo, Dauno, Epi, Ida, Mitox  *Discovery:*  Cases, 128 ± 59  Controls, 371 ± 115  *Replication:*  Cases, 220.1 ± 109.2  Controls, 189.7 ± 55.6  In doxorubicin isotoxic equivalent doses | WES with Illumina HiSeq X platform and Illumina HiSeq4000  (next-generation sequencing) | Blood;  Call rate NR,  HWE p>10^-6^ | *Discovery:*  Cases, 64  Controls, 44  *Replication:*  Cases, 3  Controls, 0 | Dexrazoxane  *Discovery:*  Cases, 2  Controls, 13  *Replication:*  Cases, 2  Controls, 2 | i) LVEF ≤50%, or >10% LVEF decline to ≤55% from a previous echocardiogram during follow-up; or  ii) LVEF ≤55% |
| Gándara-Mireles et al., 2021 | Case-control  CGS | Cases, 36  Controls, 31 | Mexico;  **Mexican** | Cases, 26/10  Controls, 20/11 | Cases, 10.67 ± 4.5  Controls, 11.2 ± 4.7  *^Age at study participation* | 8 months | **ALL** | Doxo;  Cases, 170.6 ± 49.37  Controls, cumulative dose NR | TaqMan real-time PCR with specific probes  (PCR-based) | Blood;  Call rate NR,  HWE p>0.05 | NR | NR | Decrease of 5–10% in LVEF |
| Giljeviae et al., 2010 | Prospective cohort  CGS  [Conf Abs] | Cases, 27  Controls, 4 | Croatia;  **Croatian** | 20/11 | Overall, range 1–17  *^Age at study participation* | NR | **Bone tumours**, lymphoma, NB, malignant mesenchymal tumour, WT | Doxo;  Cumulative dose NR | Real-time PCR Taqman SNP genotyping assay  (PCR-based) | Biospecimen type NR;  Quality control NR | NR | NR | CTCAE Version 2.0 |
| Gündüz et al., 2024 | Prospective cohort  CGS | Cases, 23  Controls, 37 | Turkey;  **Turkish** | Cases, 13/10  Controls, 23/14 | Cases, 14.3 ± 4.9  Controls, 13.7 ± 4.6 | Cases, 4.1 ± 1.6  Controls, 5 ± 3 | **HL,** NHL, Ewing sarcoma, OS, NB, WT | Doxo;  Cases, 290 ± 137  Controls, 230 ± 120.7 | i) Real-time PCR,  ii) TaqMan SNP genotyping assay,  iii) allelic discrimination analysis  (PCR-based) | Blood;  Call rate = 100%,  HWE p>0.05 | No chest radiotherapy | NR | FS <28% and LVEF <57%, or E/A ratio >2 or <1 |
| Hildebrandt et al., 2017 | Case-control  CGS | Cases, 46  Controls, 62  MD Anderson Cancer Centre | USA;  **White**, Hispanic, Black | Cases, 25/21  Controls, 30/32 | Cases, 9.2 ± 4.7  Controls, 9.3 ± 5.7 | Cases, 21.2 ± 11.2  Controls, 15.7 ± 7.6 | **Sarcoma**, leukaemia, lymphoma | Anthracycline type NR;  Cases, 319.5 ± 111.5  Controls, 273.9 ± 157.6  In doxorubicin isotoxic equivalent doses | TaqMan genotyping assays  (PCR-based) | Blood;  Quality control NR | Cases, 15  Controls, 14 | NR | i) LVEF 45–50%, and symptoms and other echocardiographic findings considered by a cardiologist to warrant cardiac medications; or  iii) LVEF ≤ 45%, FS ≤ 25% on ≥2 echocardiograms |
| Krajinovic et al., 2015 | Prospective cohort  CGS | *Discovery:*  251 QcALL  *Replication:*  44 DFCI | Canada;  *Discovery:*  **French-Canadian Caucasian**  *Replication:*  Ethnicity NR | *Discovery:*  134/117  *Replication:*  21/23 | *Discovery:*  5 (IQR 3–8)  *Replication:*  4 (IQR 2–6.75) | *Discovery:*  8 (IQR 5–11)  *Replication:*  5.11 (IQR 3.66–6.35) | **ALL** | Doxo;  *Discovery:*  Mean 313.1  *Replication:*  Mean 300 | PCR allele specific oligonucleotide hybridisation assay  (PCR-based) | Biospecimen type NR;  Quality control NR | NR | Dexrazoxane  *Discovery:*  70  *Replication:*  8 | Decrease in LVEF and FS  **(continuous outcome)** |
| Lipshultz et al., 2013 | Prospective cohort  CGS | 184 DFCI | USA;  Ethnicity NR | 101/83 | 6.3 (range <1–17.9) | 6.1 (range 1.0–16.1) | **ALL** | Doxo;  300 (range 204–420) | i) Pyrosequencing (PyroMark HFE),  ii) Sequenom genotyping assays,  iii) TaqMan genotyping assays  (PCR-based) | Blood;  Quality control NR | NR | Dexrazoxane  116 | i) Decrease in LV end-systolic and end-diastolic dimensions, LV mass, LV end-systolic and end-diastolic posterior wall thicknesses, LV thickness-to-dimension ratio, FS  **(continuous outcome)**  ii) cTnT >0.01 ng/mL  iii) NT-proBNP >150 pg/mL in <1 year old or >100 pg/mL in ≥1 year old |
| Liu, 2011 | Case-control  GWAS  [Thesis] | Cases, 130  Controls, 269  COG-ALTE03N1 | USA;  Ethnicity NR | NR | <21 | NR | NR | Doxo, Dauno, Ida, Mitox;  Cumulative dose NR  In doxorubicin isotoxic equivalent doses | Illumina IBCv2 BeadChip array  (microarray) | Blood;  Call rate ≥95%,  compliance with HWE | Yes;  Frequency NR | NR | Diagnosis of CHF with echocardiographic confirmation |
| McOwan et al., 2020 | Retro-spective cohort  CGS | Cases, 15  Controls, 266 | Australia;  **Australian** | Cases, 5/10  Controls, NR | Cases, 6 (IQR 3–10)  Controls, NR | Cases, 145 (IQR 71–276) days  Controls, NR | **ALL**, AML, NHL, WT, Ewing sarcoma | Doxo, Dauno, Ida, Mitox;  Cases, 200 (IQR 147–325)  Controls, cumulative dose NR  In doxorubicin isotoxic equivalent doses | Sanger sequencing of PCR amplicons  (PCR-based) | Blood;  Quality control NR | NR | NR | FS ≤24% |
| Petrykey et al., 2021 | Retro-spective cohort  CGS and EWAS | *Discovery:*  233 DFCI  *Replication:*  149 SJLIFE | Canada;  *Discovery:*  **European**  *Replication:*  **European** | *Discovery:*  114/119  *Replication:*  76/73 | *Discovery:*  4 (range 0–18)  *Replication:*  4.6 (range 0.2–17.4) | *Discovery:*  13 (range 3–24)  *Replication:*  29.7 (range 17.9–50) | **ALL** | Doxo;  *Discovery:*  209.3 (IQR 41.3–472.9)  *Replication:*  Cumulative dose NR | *Discovery:*  WES with Life Technologies SOLiD System 4.0 or Illumina HiSeq 2500 platform  *Replication:*  WGS  (next-generation sequencing) | Blood;  Call rate >80%,  HWE p>0.001 | NR | Dexrazoxane  *Discovery:*  68  *Replication:*  0 | Decrease in LVEF, FS and LV end-diastolic diameter  **(continuous outcome)** |
| Rajić et al., 2009 | Case-control  CGS | Cases, 31  Controls, 45 | Slovenia;  **Caucasian** | 32/44 | 6.3 ± 4.4 | 19.3 ± 6.3 | **ALL** | Anthracycline type NR;  199 ± 108 | i) Real-time PCR,  ii) custom TaqMan SNP genotyping assays,  iii) allelic discrimination with differently labelled fluorescent probes,  iv) multiplex PCR  (PCR-based) | Bone marrow;  Call rate NR,  compliance with HWE | NR | NR | i) Clear conduction disturbances, depolarization and repolarization changes on ECG  ii) FS <30%, LVEF <54%  iii) Abnormal E (normal 0.75 ± 0.13), A (normal 0.51 ± 0.11), E/A (normal 1.53 ± 0.4), IVRT (normal 67 ± 8), PV-A (normal 0.21 ± 0.08), PV-D (normal 0.47 ± 0.11), PV-S (normal 0.44 ± 0.1) |
| Ruiz-Pinto et al., 2017a | Case-control  GWAS | Cases, 31  Controls, 52 | Spain;  **Spanish** | Cases, 24/7  Controls, 29/23 | Cases, 10.4 (range 1.2–21.1)  Controls, 5.1 (range 1.4–16.9) | Cases, 10 (range 1–27.5)  Controls, 8.55 (range 1–24.1) | **Leukaemia**, OS, Ewing sarcoma | Doxo, Dauno, Epi; Cases, 360 (range 105–780)  Controls, 134 (range 49.2–562)  In doxorubicin isotoxic equivalent doses | Illumina HumanExome Beadchip array  (microarray) | Biospecimen type NR;  Call rate ≥99%,  HWE p≥10^-8^ | Cases, 2  Controls, 0 | NR | i) Signs/symptoms of severe mitral valve insufficiency, pericardial effusion, LV hypertrophy or pulmonary hypertension  ii) FS ≤27% if asymptomatic |
| Ruiz-Pinto et al., 2017b | Case-control  GWAS | Cases, 35  Controls, 58 | Spain;  **Spanish** | Cases, 25/10  Controls, 33/25 | Cases, 10.4 (range 1.2–21.1)  Controls, 5.1 (range 1.4–16.9) | Cases, 10.5 (range 1–27.5)  Controls, 8.3 (range 1–24.1) | **Leukaemia**, OS, Ewing sarcoma | Doxo, Dauno, Epi;  Cases, 360 (range 105–780)  Controls, 130 (range 49.2–562)  In doxorubicin isotoxic equivalent doses | i) Illumina HumanExome BeadChip array,  ii) Sanger sequencing  (microarray) | Blood;  Call rate ≥99%,  HWE p≥10^-8^ | Cases, 6  Controls, 3 | NR | i) Signs/symptoms of severe mitral valve insufficiency, pericardial effusion, LV hypertrophy or pulmonary hypertension  ii) FS ≤27% if asymptomatic |
| Sági et al., 2018 | Case-control  CGS | Cases, 20  Controls, 641 | Hungary;  **Hungarian** | 399/262 | 5.3 (range 0–18) | NR | **ALL**, OS | Doxo, Dauno;  Range 60–840  In doxorubicin isotoxic equivalent doses | i) TaqMan Open-Array Genotyping System,  ii) KASPar assays  (PCR-based) | Blood;  Call rate >87.5%,  HWE p>8.9x10^-3^ | NR | NR | FS ≤ 28% |
| Sapkota et al., 2021 | Retro-spective cohort  GWAS | *Discovery:*  246 SJILIFE  *Replication:*  1645 SJLIFE | USA;  *Discovery:*  **African**  *Replication:*  **European** | *Discovery:*  128/118  *Replication:*  878/767 | *Discovery:*  8.8 (range 0.1–19.7)  *Replication:*  7.7 (range 0–23.6) | NR | **Leukaemia**, bone sarcoma, CNS malignancy, germ cell tumour, HL, NHL, liver malignancies, melanoma, nasopharyngeal carcinoma, other carcinoma, retinoblastoma, STS, WT | Anthracycline type NR;  *Discovery:*  99.4 (range 0–689.5)  *Replication:*  100 (range 0–694.7)  In doxorubicin isotoxic equivalent doses | WGS with Illumina HiSeq X10 sequencers  (next-generation sequencing) | Blood;  Call rate >95%,  HWE p>10^-10^ | *Discovery:*  153  *Replication:*  1028 | NR | Classified as mild (grade 1), moderate (grade 2), severe or disabling (grade 3), life- threatening (grade 4), or fatal (grade 5) according to modified CTCAE Version 4.03 |
| Sapkota et al., 2022 | Retro-spective cohort  GWAS | *Discovery:*  Cases, 227  Controls, 1643  SJLIFE  *Replication 1:*  Cases, 43  Controls, 258  SJLIFE  *Replication 2:*  Cases, 230  Controls, 3790  CCSS | USA;  *Discovery:*  **European**  *Replication 1:*  **African**  *Replication 2:*  **European** | *Discovery:*  Cases, 135/92  Controls, 851/792  *Replication 1:*  Cases, 32/11  Controls, 113/145  *Replication 2:*  Cases, 96/134  Controls, 1823/1967 | *Discovery:*  Cases, 10.5 (range 0–20.5)  Controls, 6.9 (range 0–23.6)  *Replication 1:*  Cases, 9.4 (range 1.8–18.6)  Controls, 8 (range 0–19.7)  *Replication 2:*  Cases, 12 (range 0.1–20.8)  Controls, 6.8 (range 0–20.9) | *Discovery:*  Cases, 28.9 (range 11.4-53.8)  Controls, 24.7 (range 7.7–49.8)  *Replication 1:*  Cases, 27.3 (range 11.3–48.3)  Controls, 22.9 (range 10.2–49.7)  *Replication 2:*  Cases, 22.4 (range 0–42.7)  Controls, NR | **Leukaemia**, CNS malignancy, HL, NHL, WT, NB, STS, malignant bone tumour | Doxo, Dauno;  *Discovery:*  0: 64 cases, 536 controls  1–250: 90 cases, 875 controls  >250: 73 cases, 232 controls  *Replication 1:*  0: 13 cases, 104 controls  1–250: 13 cases, 122 controls  >250: 17 cases, 32 controls  *Replication 2:*  0: 82 cases, 2585 controls  1–250: 44 cases, 623 controls  >250: 104 cases, 582 controls  In doxorubicin isotoxic equivalent doses | WGS with Illumina HiSeq X10 sequencers  (next-generation sequencing) | Blood;  Call rate >95%,  HWE p>10^-10^ | *Discovery:*  Cases, 143  Controls, 885  *Replication 1:*  Cases, 28  Controls, 125  *Replication 2:*  Cases, 170  Controls, 2385 | NR | *Discovery and Replication 1:*  Classified as moderate (grade 2: resting EF < 50%–40% or 10%–19% absolute drop from baseline),  severe or disabling (grade 3: resting EF < 39%–20%, or >20% absolute drop from baseline, or medication initiated),  life-threatening (grade 4: resting EF < 20%; refractory or poorly controlled heart failure; intervention such as ventricular assist device, intravenous vasopressor support; or heart transplant indicated), or  fatal (grade 5: death), according to modified CTCAE Version 4.03  *Replication 2:*  Self-reported diagnosis of CHF, classified as moderate (grade 2: self-reported CHF not requiring medication), severe or disabling (grade 3: cardio- myopathy of CHF requiring medication), life-threatening (grade 4: cardiac transplantation), or fatal (grade 5: death as a result of heart failure) according to CTCAE |
| Scott et al., 2020 | Prospective cohort  GWAS  [Conf Abs] | 280 | Canada;  Ethnicity NR | NR | Children | NR | NR | NR | i) IMPUTE2,  ii) TaqMan genotyping assays  (PCR-based) | Biospecimen type NR;  Quality control NR | NR | NR | NR |
| Semsei et al., 2012 | Retro-spective cohort  CGS | 235 | Hungary;  **Hungarian** | 126/109 | 5.7 ± 3.8 | 6.3 (range 2.4–13.7) | **ALL** | Doxo, Dauno;  120–360 | i) Mini-sequencing,  ii) GenomeLab SNPstream genotyping platform  (PCR-based) | Blood, bone marrow;  Call rate NR,  HWE p≥0.01 | NR | Dexrazoxane  69 | Decrease in FS  **(continuous outcome)** |
| Sharafeldin et al., 2023 | Case-control  GWAS | *Discovery:*  Cases, 129  Controls, 149  COG-ALTE03N1  *Replication 1:*  Cases, 32  Controls, 173  COG-ALTE03N1  *Replication 2:*  N/A, as adults at diagnosis  *Replication 3:*  Cases, 229  Controls, 5360  CCSS | USA;  *Discovery:*  **White**, Asian, Black, Hispanic  *Replication 1:*  **White**, Asian, Black, Hispanic  *Replication 2:*  N/A, as adults at diagnosis  *Replication 3:*  **White** | *Discovery:*  Cases, 58/71  Controls, 80/69  *Replication 1:*  Cases, 12/20  Controls, 88/85  *Replication 2:*  N/A, as adults at diagnosis  *Replication 3:*  Cases, 95/134  Controls, 2617/2743 | *Discovery:*  Cases, 7.80 ± 5.62  Controls, 7.9 ± 5.9  *Replication 1:*  Cases, 8.66 ± 5.13  Controls, 8.1 ± 5.6  *Replication 2:*  N/A, as adults at diagnosis  *Replication 3:*  Cases, 11.5 ± 5.6  Controls, 7.7 ± 5.9 | *Discovery:*  Cases, 9.97 ± 8.68  Controls, 15.8 ± 8.7  *Replication 1:*  Cases, 8.88 ± 9.51  Controls, 12.5 ± 7.9  *Replication 2:*  N/A, as adults at diagnosis  *Replication 3:*  Cases, 23.8 ± 10.2  Controls, 32.7 ± 6 | **ALL**, AML, brain tumour, Ewing sarcoma, HL, NHL, NB, OS, sarcoma, WT | Doxo, Dauno, Epi, Ida;  *Discovery:*  Cases, 311.8 ± 136.6  Controls, 185.8 ± 167.4  *Replication 1:*  Cases, 279.3 ± 143.6  Controls, 200.9 ± 170.2  *Replication 2:*  N/A, as adults at diagnosis  *Replication 3:*  Cases, 232 ± 221  Controls, 100.1 ± 160.8  In doxorubicin isotoxic equivalent doses | *Discovery:*  WES with Illumina NovaSeq  *Replication 1:*  Juno system, 96.96 Genotyping IFCs  *Replication 2:*  N/A, as adults at diagnosis  *Replication 3:*  Illumina HumanOmni5Exome array  (next-generation sequencing) | Blood, saliva;  Call rate ≥95%,  HWE p≥10^-6^ | *Discovery:*  Cases, 46  Controls, 37  *Replication 1:*  Cases, 7  Controls, 31  *Replication 2:*  N/A, as adults at diagnosis  *Replication 3:*  Cases, 115  Controls, 1248 | NR | *Discovery and Replication 1:*  i) Signs/symptoms of cardiac compromise (dyspnoea, orthopnoea, fatigue, oedema, hepatomegaly, and/or rales) according to  AHA criteria;  ii) LVEF ≤40%, FS ≤28% if asymptomatic  *Replication 2:*  N/A, as adults at diagnosis  *Replication 3:*  Self-reported cardiomyopathy or HF, use of medications, heart transplant |
| Siemens et al., 2023 | Case-control  CGS | Cases, 139  Controls, 456  CPNDS | Canada;  **Canadian**, Dutch, American | Cases, 73/66  Controls, 242/214 | Cases, 8 (range 0.04–18)  Controls, 5.2 (range 0.1–17.7) | NR | **ALL**, AML, Ewing sarcoma, HB, HL, NHL, NB, OS, RMS, WT | Anthracycline type NR;  Cases, 300 (range 36–840)  Controls, 200 (range 25–720)  In doxorubicin isotoxic equivalent doses | Infinium Global Screening Array-24 with Multi-Disease custom add-on array (microarray) | Blood, saliva;  Quality control NR | Cases, 31  Controls, 72 | Dexrazoxane  Cases, 4  Controls, 14 | FS ≤26% (cardiotoxicity of Grade ≥2 according to modified CTCAE Version 3) |
| Singh et al., 2020 | Case-control  CGS | Cases, 75  Controls, 92  COG-ALTE03N1 | USA;  Ethnicity NR | Cases, 32/43  Controls, 52/40 | Cases, 7.8 (range 3.8–11.5)  Controls, 9.6 (range 3.3–14.8) | Cases, 6 (range 1.3–11.6)  Controls, 12 (range 7.4–17.2) | **Bone cancer**, ALL, AML, HL, NHL, WT, NB, sarcoma | Anthracycline type NR;  Cases, 300 (range 230.3–375)  Controls, 255 (range 150–368)  In doxorubicin isotoxic equivalent doses | Multiplex real-time PCR assay  (PCR-based) | Blood;  Quality control NR | Cases, 26  Controls, 24 | NR | Signs/symptoms of cardiac compromise (dyspnoea, orthopnoea, fatigue, oedema, hepatomegaly, and/or rales) according to  AHA criteria |
| Singh et al., 2023a | Case-control  CGS | Cases, 40  Controls, 64  COG-ALTE03N1 | USA;  **Non-Hispanic White**, Hispanic, Black, Asian | Cases, 16/24  Controls, 30/34 | Cases, 8.2 (IQR 3.6–13.9)  Controls, 9.7 (IQR 3.3–14.4) | Cases, 5.3 (IQR 0.8–12.8)  Controls, 10.1 (IQR 7.1–14.5) | **ALL**, AML, Ewing sarcoma, HL, kidney tumours, NB, NHL, OS, STS | Anthracycline type NR;  0–250: 15 cases, 41 controls  ≥250: 25 cases, 23 controls  In doxorubicin isotoxic equivalent doses | i) PCR,  ii) Juno system, 96.96 Genotyping IFCs  (PCR-based) | Blood, saliva;  Quality control NR | Cases, 19  Controls, 13 | NR | i) Signs/symptoms of cardiac compromise (dyspnoea, orthopnoea, fatigue, oedema, hepatomegaly, and/or rales) according to  AHA criteria; or  ii) LVEF ≤40%, FS ≤28% if asymptomatic |
| Tonorezos et al., 2017 | Case-control  CGS  [Conf Abs] | Cases, 41  Controls, 276 | USA;  **Non-Hispanic White** | 218/237 | Median 15 | Median 17.8 | **HL**, sarcoma, leukaemia | NR | Low density SNP array – Illumina Exomearray  (microarray) | Biospecimen type NR;  Quality control NR | NR | NR | LVEF <55% |
| Vargas-Neri et al., 2022 | Case-control  CGS | Cases, 4  Controls, 75 | Mexico;  **Mexican** | Cases, 1/3  Controls, 38/37 | Cases, 7.9 (IQR 7.3–13.9)  Controls, 5.3 (IQR 2.9–9.6)  *^Age at study participation* | Cases, 20.5 (IQR 10–42.3) days  Controls, 883 (IQR 121–1722) days | **ALL**, AML, NHL, HL, OS, WT, HB, NB, endodermal sinus tumour, germ tumour | Doxo, Dauno, Epi, Ida, Mitox;  Cases, 289.6 (IQR 70–506)  Controls, 175 (IQR 120–256.3)  In doxorubicin isotoxic equivalent doses | Illumina Global Screening Array v2.0 with Multi-Disease array  (microarray) | Saliva/buccal swabs;  Quality control NR | Cases, 1  Controls, 16 | Dexrazoxane  Cases, 1  Controls, 29 | FS ≤26% |
| Visscher et al., 2012 | Case-control  CGS | *Discovery:*  Cases, 38  Controls, 118  *Replication 1:*  Cases, 40  Controls, 148  CPNDS  *Replication 2:*  Cases, 43  Controls, 53  Dutch-EKZ | Canada;  *Discovery:*  **Canadian**  *Replication 1:*  **Canadian**  *Replication 2:*  **Dutch** | *Discovery:*  Cases, 17/21  Controls, 66/52  *Replication 1:*  Cases, 22/18  Controls, 82/66  *Replication 2:*  Cases, 22/21  Controls, 27/26 | *Discovery:*  Cases, 5.5 (range 0.04–17)  Controls, 3.9 (range 0.5–16.5)  *Replication 1:*  Cases, 6.2 (range 0.4–17.6)  Controls, 3.7 (range 0.05–16.9)  *Replication 2:*  Cases, 9 (range 0.5–16.8)  Controls, 10.6 (range 2.1–17.7)  *^Age at study participation* | *Discovery:*  Cases, 6.5 (range 0.1–21.2)  Controls, 7.8 (range 5–17.9)  *Replication 1:*  Cases, 7.4 (range 0.2–20.7)  Controls, 9.2 (range 5–18.6)  *Replication 2:*  Cases, 20.2 (range 7.4–27.9)  Controls, 15.4 (range 5.1–29.8) | **ALL**, AML, other leukaemia, HL, NHL, OS, RMS, Ewing sarcoma, other sarcoma, WT, HB, NB, carcinoma | Doxo, Dauno, Epi, Ida, Mitox;  *Discovery:*  Cases, 300 (range 36–540)  Controls, 175 (range 60–600)  *Replication 1:*  Cases, 270 (range 45–840)  Controls, 250 (range 25–600)  *Replication 2:*  Cases, 360 (range 100–720)  Controls, 300 (range 50–720)  In doxorubicin isotoxic equivalent doses | *Discovery and Replication 1:*  Customised Illumina GoldenGate SNP genotyping assay  *Replication 2:*  TaqMan SNP genotyping  (microarray) | Blood, saliva or buccal swabs;  Call rate NR,  HWE p>1.5x10^-4^ | *Discovery:*  Cases, 6  Controls, 8  *Replication 1:*  Cases, 12  Controls, 29  *Replication 2:*  Cases, 10  Controls, 13 | NR | i) Signs/symptoms requiring intervention according to CTCAE Version 3; and/or  ii) FS ≤26% |
| Visscher et al., 2013 | Case-control  CGS | *Discovery:*  Cases, 78  Controls, 266 CPNDS  *Replication:*  Cases, 56  Controls, 162  Dutch-EKZ + CPNDS | Canada;  *Discovery:*  **Canadian**  *Replication:*  **Dutch**, Canadian | *Discovery:*  Cases, 39/39  Controls, 148/118  *Replication:*  Cases, 31/25  Controls, 75/87 | *Discovery:*  Cases, range 0.04–17.6  Controls, range 0.05–16.9  *Replication:*  Cases, range 0.5–17.0  Controls, range 0.5–17.7  *^Age at study participation* | *Discovery:*  Cases, range 0.1–21.2  Controls, range 5–18.6  *Replication:*  Cases, range 0.4–28.5  Controls, range 5–31.6 | **ALL**, AML, other leukaemia, HL, NHL, OS, RMS, Ewing sarcoma, other sarcoma, WT, HB, NB, carcinoma, germ cell tumour | Doxo, Dauno, Epi, Ida, Mitox;  *Discovery:*  Cases, range 36–840  Controls, range 25–600  *Replication:*  Cases, range 100–720  Controls, range 50–720  In doxorubicin isotoxic equivalent doses | Custom 96-plex Illumina Veracode GoldenGate SNP genotyping assay  (microarray) | Blood, saliva or buccal swabs;  Call rate >98%,  HWE p>0.05 | *Discovery:*  Cases,  Controls,  *Replication:*  Cases, 13  Controls, 37 | NR | i) Signs/symptoms requiring intervention according to CTCAE Version 3; and/or  ii) FS ≤26% |
| Visscher et al., 2015 | Case-control  CGS | *Discovery:*  Cases, 78  Controls, 266 CPNDS  *Replication:*  Cases, 56  Controls, 162  Dutch-EKZ + CPNDS | Canada;  *Discovery:*  **Canadian**  *Replication:*  **Dutch**, Canadian | *Discovery:*  Cases, 39/39  Controls, 148/118  *Replication:*  Cases, 31/25  Controls, 75/87 | *Discovery:*  Cases, range 0.04–17.6  Controls, range 0.05–16.9  *Replication:*  Cases, range 0.5–17.0  Controls, range 0.5–17.7  *^Age at study participation* | *Discovery:*  Cases, range 0.1–21.2  Controls, range 5–18.6  *Replication:*  Cases, range 0.4–28.5  Controls, range 5–31.6 | **ALL**, AML, other leukaemia, HL, NHL, OS, RMS, Ewing sarcoma, other sarcoma, WT, HB, NB, carcinoma, germ cell tumour | Doxo, Dauno, Epi, Ida, Mitox;  *Discovery:*  Cases, range 36–840  Controls, range 25–600  *Replication:*  Cases, range 100–720  Controls, range 50–720  In doxorubicin isotoxic equivalent doses | i) Customised Illumina GoldenGate SNP genotyping assay,  ii) custom 96-SNP Illumina Veracode GoldenGate genotyping assay  (microarray) | Blood, saliva or buccal swabs;  Call rate = 99.8%,  compliance with HWE | *Discovery:*  Cases,  Controls,  *Replication:*  Cases, 13  Controls, 37 | NR | i) Signs/symptoms requiring intervention according to CTCAE Version 3; and/or  ii) FS ≤26% |
| Wang et al., 2014 | Case-control  GWAS | *Discovery:*  Cases, 93  Controls, 194  COG-ALTE03N1  *Replication:*  N/A, as no controls | USA;  *Discovery:*  **Non-Hispanic White**  *Replication:*  N/A, as no controls | *Discovery:*  Cases, 40/53  Controls, 94/100  *Replication:*  N/A, as no controls | *Discovery:*  Cases, 6.9 (range 0–20.2)  Controls, 6.3 (range 0–20.6)  *Replication:*  N/A, as no controls | *Discovery:*  Cases, 10 (range 0.1–35.1)  Controls, 11.3 (range 0.9–41)  *Replication:*  N/A, as no controls | **ALL**, HL, NHL, bone tumours, STS , AML | Doxo, Dauno, Epi, Ida, Mitox;  *Discovery:*  Cases, 300 (range 0–630)  Controls, 152 (range 0–825)  *Replication:*  N/A, as no controls  In doxorubicin isotoxic equivalent doses | *Discovery:*  Illumina IBC cardiovascular SNP array  *Replication:*  N/A, as no controls  (microarray) | Blood, buccal cells/saliva;  Call rate >95%,  HWE p≥0.000001 | *Discovery:*  Cases, 23  Controls, 22  *Replication:*  N/A, as no controls | NR | i) Signs/symptoms of cardiac compromise (dyspnoea, orthopnoea, fatigue, oedema, hepatomegaly, and/or rales) according to  AHA criteria;  ii) LVEF ≤40%, FS ≤28% if asymptomatic |
| Wang et al., 2016 | Case-control  GWAS | *Discovery:*  Cases, 112  Controls, 219  COG-ALTE03N1  *Replication:*  N/A, as no controls | USA;  *Discovery:*  **Non-Hispanic White**  *Replication:*  N/A, as no controls | *Discovery:*  Cases, 46/66  Controls, 106/113  *Replication:*  N/A, as no controls | *Discovery:*  Cases, 7.5 (range 0–20)  Controls, 7.9 (range 0–21)  *Replication:*  N/A, as no controls | *Discovery:*  Cases, 9.4 (range 0.1–35.1)  Controls, 12.9 (range 1.4–41)  *Replication:*  N/A, as no controls | **Sarcoma,** HL, NHL, ALL, AML | Anthracycline type NR;  *Discovery:*  Cases, 319 (range 0–760)  Controls, 180 (range 0–825)  *Replication:*  N/A, as no controls | *Discovery:*  Illumina HumanOmniExpress-12 v1.0 DNA analysis bead-chip  *Replication:*  N/A, as no controls  (microarray) | Blood, buccal cells/saliva;  Call rate >99.8%,  HWE p≥0.0001 | *Discovery:*  Cases, 25  Controls, 27  *Replication:*  N/A, as no controls | NR | i) Signs/symptoms of cardiac compromise (dyspnoea, orthopnoea, fatigue, oedema, hepatomegaly, and/or rales) according to  AHA criteria;  ii) LVEF ≤40%, FS ≤28% if asymptomatic |
| Wang et al., 2019 | Case-control  CGS  [Conf Abs] | *Discovery:*  Cases, 155  Controls, 256  COG-ALTE03N1  *Replication:*  Cases, 229  Controls, N/A, as half of the participants had not been treated with anthracyclines  CCSS | USA;  Ethnicity NR | NR | *Discovery:*  Cases, 7.2 (range 0–21)  Controls, 7.6 (range 0–2)  *Replication:*  Cases, 13 (range 0–20)  Controls, N/A, as half of the participants had not been treated with anthracyclines | NR | NR | Anthracycline type NR;  *Discovery:*  Cases, 340 (range 0–760)  Controls, 175 (range 0–825)  *Replication:*  Cases, 230 (range 0–918)  Controls, N/A, as half of the participants had not been treated with anthracyclines (median cumulative dose = 0) | NR | NR | *Discovery:*  Cases, 35  Controls, 33  *Replication:*  Cases, 115  Controls, N/A, as half of the participants had not been treated with anthracycline | NR | NR |
| Wang et al., 2023 | Case-control  GWAS | *Discovery:*  Cases, 126  Controls, 1740  CCSS  *Replication:*  Cases, 105  Controls, 160  COG-ALTE03N1 | USA;  *Discovery:*  **Non-Hispanic White**  *Replication:*  **Non-Hispanic White**, Hispanic, African American | *Discovery:*  Cases, 75/51  Controls, 847/893  *Replication:*  Cases, 47/58  Controls, 85/75 | *Discovery:*  Cases, 7 (range 0–20)  Controls, 11 (range 0–20)  *Replication:*  Cases, 6.6 (range 0–20.6)  Controls, 8.6 (range 0.3–21.7) | *Discovery:*  Cases, 22 (range 0–39)  Controls, 31 (range 14–45)  *Replication:*  Cases, 7.1 (range 0.1–27.7)  Controls, 10.7 (range 1.2–33) | **ALL**, AML, HL, NHL, bone tumours, STS | Doxo, Dauno, Epi, Ida, Mitox;  *Discovery:*  Cases, 362.1 (range 50–917.8)  Controls, 277.9 (range 10–1120)  *Replication:*  Cases, 7.1 (range 0.1–27.7)  Controls, 10.7 (range 1.2–33)  In doxorubicin isotoxic equivalent doses | *Discovery:*  Illumina HumanOmni5Exome Array  *Replication:*  Juno system  (microarray) | Blood, saliva;  Call rate NR,  HWE p≥0.000005 | *Discovery:*  Cases, 43  Controls, 318  *Replication:*  Cases, 29  Controls, 29 | NR | *Discovery:*  Self-reported cardiomyopathy, CHF, heart transplantation, and medications graded as Grades 3–5 according to CTCAE  *Replication:*  i) Signs/symptoms of cardiac compromise (dyspnoea, orthopnoea, fatigue, oedema, hepatomegaly, and/or rales) according to  AHA criteria;  ii) LVEF ≤40%, FS ≤28% if asymptomatic |
| Yunis et al., 2022 | Prospective cohort  CGS | 51 | Colombia;  **Colombian** | 30/21 | 10 (range 0.15–18)  *^Age at study participation* | NR | **AML** | Dauno;  At least 360 | i) SNaPshot assay,  ii) multiplex PCR,  iii) conventional PCR  (PCR-based) | Biospecimen type NR;  Quality control NR | NR | NR | Cardiotoxicity (Grades 3–4) according to CTCAE version 5.0 |
| **Cost-effectiveness study** | | | | | | | | | | | | | |
| Dionne et al., 2017 | Decision model-based full economic evaluation with cost-effective-ness analysis | Hypothetical cohort, 100 | Canada (in Canadian dollars);  Ethnicity NR | NR | Mean 6  *^Age at start of treatment* | Range 1–25 | **ALL**, AML, Ewing sarcoma, WT, OS, other sarcoma, HL, NHL, NB, HB | NR | NR | NR | NR | Dexrazoxane  *Experimental model:*  100% high risk patients  *Usual care:* 9.1% high risk patients | i) Stable asymptomatic cardiac damage:  no clinical symptoms, but damage is identified through testing and does not worsen over time  ii) Symptomatic cardiac damage:  cardiac damage that progressed from asymptomatic to cardiomyopathy and CHF, which may cause death or require heart transplant |

**Bold** text indicates the most frequent ethnicity and caner type in the study population.

Values of age at cancer diagnosis, length of follow-up and cumulative anthracycline dose are expressed in mean±SD or median (IQR/range).

**Abbreviations:** AHA, American Heart Association; ALL, acute lymphoblastic leukaemia; AML, acute myeloid leukaemia; ARMS, amplification refractory mutation system; CCSS, Childhood Cancer Survivor Study; CGS, candidate gene study; COG, Children’s Oncology Group; Conf Abs, conference abstract; CPNDS, Canadian Pharmacogenomics Network for Drug Safety; CTCAE, Common Terminology Criteria for Adverse Events; Dauno, daunorubicin; DFCI, Dana-Farber Cancer Institute; Doxo, doxorubicin; Epi, epirubicin; EWAS, exome-wide association study; FS, fractional shortening; GWAS, genome-wide association study; HB, hepatoblastoma; HL, Hodgkin lymphoma; HWE, Hardy-Weinberg equilibrium; Ida, idarubicin; IQR, inter-quartile range; LVEF, left ventricular ejection fraction; Mitox, mitoxantrone; NB, neuroblastoma; NHL, Non-Hodgkin lymphoma; NR, not reported; OS, osteosarcoma; PCR, polymerase chain reaction; RFLP, restriction fragment length polymorphism; RMS, rhabdomyosarcoma; SJLIFE, St. Jude Lifetime Cohort; SNP, single nucleotide polymorphism; STS, soft tissue sarcoma; WES, whole exome sequencing; WGS, whole genome sequencing; WT, Wilms tumour.

**Supplementary Table S21.** Characteristics of excluded studies. Contains studies which might appear to meet the inclusion criteria or are thought to be relevant, but which were excluded.

| **Study** | **Title** | **Reason for exclusion** |
| --- | --- | --- |
| **Clinical effectiveness studies** | | |
| Armenian et al., 2013 | Genetic susceptibility to anthracycline‐related congestive heart failure in survivors of haematopoietic cell transplantation | Not children at cancer diagnosis |
| Ater et al., 2016 | PD-061 Relationships among Hypertension Susceptibility Loci, Hypertension, and Late Anthracycline-Related Cardiotoxicity in Long-Term Childhood Cancer Survivors | Conference abstract of the included Hildebrandt et al., 2017 study |
| Bhavsar et al., 2016 | O-074 Pharmacogenomic Strategies for the Prevention of Anthracycline-Induced Heart Failure: Validation of a Genetic Association with a Non-Synonymous Variant in RARG | Conference abstract of the included Aminkeng et al., 2015 study |
| Chaix et al., 2018 | Genomic Factors Associated With Anthracycline Cardiotoxicity in Pediatric Cancer Survivors | Conference abstract of the included Chaix et al., 2020 study |
| Gándara-Mireles et al., 2022 | Impact of single-nucleotide variants and nutritional status on population pharmacokinetics of Doxorubicin, and its effect on cardiotoxicity in children with leukemia | Duplicate publication of the included Gándara-Mireles et al., 2021 study |
| Garcia-Pavia et al., 2019 | Genetic Variants Associated With Cancer Therapy–Induced Cardiomyopathy | No control group: all participants had ACT |
| Güntürkün et al., 2021 | Artificial Intelligence–Assisted Prediction of Late-Onset Cardiomyopathy Among Childhood Cancer Survivors | Did not use genetic techniques:  Genetic algorithm (GA) is a computer-based rather than genetic technique, and GA was used to select only ECG and clinical variables to predict ACT. Therefore, no genetic techniques or variables were involved in predicting ACT. |
| Hellmann et al., 2020 | Genetic Polymorphisms Affecting Cardiac Biomarker Concentrations in Children with Cancer: an Analysis from the “European Paediatric Oncology Off-patents Medicines Consortium” (EPOC) Trial | ACT was defined based on cardiac biomarkers alone. Authors also acknowledged that “the role of cardiac biomarkers in the evaluation of anthracycline-induced cardiotoxicity is still a matter of debate”. |
| Hertz et al., 2016 | Evidence for association of SNPs in ABCB1 and CBR3, but not RAC2, NCF4, SLC28A3 or TOP2B, with chronic cardiotoxicity in a cohort of breast cancer patients treated with anthracyclines | Not children at cancer diagnosis |
| Huang et al., 2017 | Effects of cytochrome P450 family 3 subfamily A member 5 gene polymorphisms on daunorubicin metabolism and adverse reactions in patients with acute leukemia | Studied the genetic association with mRNA expression, enzyme activity and plasma anthracycline concentration, but not ACT |
| Kitagawa et al., 2012 | Prospective evaluation of corrected QT intervals and arrhythmias after exposure to epirubicin, cyclophosphamide, and 5-fluorouracil in women with breast cancer | Not children at cancer diagnosis |
| Lares-Asseff et al., 2021 | Association of Genetic Polymorphisms NCF4 rs1883112, CBR3 rs1056892, and ABCC1 rs3743527 with the Cardiotoxic Effects of Doxorubicin | Conference abstract of the included Gándara-Mireles et al., 2021 study |
| Leger et al., 2017 | Circulating microRNAs: Potential Markers of Cardiotoxicity in Children and Young Adults Treated With Anthracycline Chemotherapy | RNA-based genetic techniques |
| Loucks et al., 2021 | Pharmacogenetic testing to guide therapeutic decision-making and improve outcomes for children undergoing anthracycline-based chemotherapy | Case series |
| Lubieniecka et al., 2012 | Single-Nucleotide Polymorphisms in Aldo-Keto and Carbonyl Reductase Genes Are Not Associated with Acute Cardiotoxicity after Daunorubicin Chemotherapy | Not children at cancer diagnosis |
| Lubieniecka et al., 2013 | A discovery study of daunorubicin induced cardiotoxicity in a sample of acute myeloid leukemia patients prioritizes P450 oxidoreductase polymorphisms as a potential risk factor | Not children at cancer diagnosis |
| Magdy et al., 2022 | Identification of Drug Transporter Genomic Variants and Inhibitors That Protect Against Doxorubicin-Induced Cardiotoxicity | Not humans: genetic association was studied in children-derived cardiomyocytes, but not the children themselves |
| Oatmen et al., 2018 | Identification of a novel microRNA profile in pediatric patients with cancer treated with anthracycline chemotherapy | RNA-based genetic techniques |
| Osterweil, 2010 | Genes raise risk of anthracycline-related cardiotoxicity | News article of the included Blanco et al., 2012 study |
| Ragab et al., 2024 | CELF4 (rs1786814) gene polymorphism and speckle-tracking Echocardiography for cardiovascular complications in childhood cancer survivors | Healthy children were used as controls |
| Reichwagen et al., 2015 | Association of NADPH oxidase polymorphisms with anthracycline-induced cardiotoxicity in the RICOVER-60 trial of patients with aggressive CD20+ B-cell lymphoma | Not children at cancer diagnosis |
| Reinbolt et al., 2015 | Risk factors for anthracycline-associated cardiotoxicity | Not children at cancer diagnosis |
| Rossi et al., 2009 | Analysis of the host pharmacogenetic background for prediction of outcome and toxicity in diffuse large B-cell lymphoma treated with R-CHOP21 | Not children at cancer diagnosis |
| Ruiz-Pinto et al., 2016 | Exome Array Analysis Identifies New Loci And Low-Frequency Variants Associated With Anthracycline-Induced Cardiotoxicity | Conference abstract of the included Ruiz-Pinto et al., 2017b study |
| Sachidanandam et al., 2012 | Unexpected doxorubicin-mediated cardiotoxicity in sisters: Possible role of polymorphisms in histamine n-methyl transferase | Case report |
| Salanci et al., 2010 | OP-046 The Relation between Functional Cardiac Parameters and Single Nucleotide Polmorphisms [*sic*] in Glutathione S Transferase P1 and Carbonyl Reductase3 Genes | Participant age not reported. However, since the majority (76.5%) have breast cancer, which is very rare in children (1 in 1,000,000 (Kennedy and Boughey, 2013)), we assumed that such majority of participants are adults. |
| Salanci et al., 2013 | The relationship between changes in functional cardiac parameters following anthracycline therapy and carbonyl reductase 3 and glutathione S transferase Pi polymorphisms | Not children at cancer diagnosis |
| Samosir et al., 2021 | Risk Factors of Daunorubicine [*sic*] Induced Early Cardiotoxicity in Childhood Acute Lymphoblastic Leukemia: A Retrospective Study | Did not use genetic techniques |
| Semsei, 2011 | Transzportfehérjék genetikai polimorfizmusainak szerepe akut limfoid leukémiában; farmakogenetikai vizsgálatok *[translated as: The role of genetic polymorphisms of transport proteins in acute lymphoid leukaemia; pharmacogenetic studies]* | Duplicate publication of the included Semsei et al., 2012 study |
| Singh et al., 2023b | Altered Peripheral Blood Gene Expression in Childhood Cancer Survivors With Anthracycline‐Induced Cardiomyopathy – A COG‐ALTE03N1 Report | RNA-based genetic techniques |
| Singh et al., 2023c | Identification of novel hypermethylated or hypomethylated CpG sites and genes associated with anthracycline-induced cardiomyopathy | DNA methylation is an epigenetic change, which does not affect the genetic sequence |
| Skitch et al., 2017 | Novel approaches to the prediction, diagnosis and treatment of cardiac late effects in survivors of childhood cancer: a multi-centre observational study | Study protocol of the included Chaix et al., 2020 study |
| Svyatova et al., 2023 | Genes of Predisposition to Childhood Beta-Cell Acute Lymphoblastic Leukemia in the Kazakh Population | Studied the genetic association with risk of childhood cancer, but not ACT |
| Tron et al., 2021 | Could pharmacogenetics testing explain anthracycline-induced cardiotoxicity sensitivity in hematologic pediatric patients? | Case report |
| Vinodhini et al., 2018 | Evaluation of a polymorphism in MYBPC3 in patients with anthracycline induced cardiotoxicity | No control group: all participants had ACT |
| Vivenza et al., 2018 | Role of the Renin-Angiotensin-Aldosterone System and the Glutathione S-Transferase Mu, Pi and Theta Gene Polymorphisms in Cardiotoxicity after Anthracycline Chemotherapy for Breast Carcinoma | Not children at cancer diagnosis |
| Völler et al., 2015 | Age-Dependent Pharmacokinetics of Doxorubicin in Children with Cancer | Studied the genetic association with anthracycline clearance, but not ACT |
| Vulsteke et al., 2015 | Clinical and genetic risk factors for epirubicin-induced cardiac toxicity in early breast cancer patients | Not children at cancer diagnosis |
| Wang et al., 2015 | CELF4 variant and Anthracycline-related Cardiomyopathy (anth-card) – A COG Study (ALTE03N1) | Conference abstract of the included Wang et al., 2016 study |
| Wasielewski et al., 2014 | Potential genetic predisposition for anthracycline-associated cardiomyopathy in families with dilated cardiomyopathy | No control group: all participants had ACT |
| Weiss et al., 2006 | Glutathione S-transferase (GSTM1, GSTT1 and GSTA1) polymorphisms and outcomes after treatment for acute myeloid leukemia: pharmacogenetics in Southwest Oncology Group (SWOG) clinical trials | Not children at cancer diagnosis |
| Windsor et al., 2012 | Germline genetic polymorphisms may influence chemotherapy response and disease outcome in osteosarcoma: A pilot study | Not children at cancer diagnosis:  Median age at diagnosis is 18 years (range 10-51), meaning 50% of the 58 patients would have been above 18 years. |
| Wojnowski et al., 2005 | NAD(P)H Oxidase and Multidrug Resistance Protein Genetic Polymorphisms Are Associated With Doxorubicin-Induced Cardiotoxicity | Not children at cancer diagnosis |
| **Cost-effectiveness study** | | |
| Ehrhardt et al., 2020 | Cost-Effectiveness of the International Late Effects of Childhood Cancer Guideline Harmonization Group Screening Guidelines to Prevent Heart Failure in Survivors of Childhood Cancer | Did not use genetic techniques |

**Supplementary Table S22.** Characteristics of ongoing studies. Contains studies which are ongoing and potentially eligible for future inclusion.

| **Study** | **Title** | **Status** |
| --- | --- | --- |
| **Clinical effectiveness studies** | | |
| Kissoon, 2019 | Using Pharmacogenomics Testing to Optimize Care in Children with Acute Hematologic Malignancies | The study may not have reached its specified endpoint. Results were not reported in this conference abstract. LYFW contacted authors; no reply received. |
| Salazar, 2024  (NCT04036045) | Approaches to Identify Early Biomarkers and Pathogenesis of Anthracycline Cardiotoxicity | Estimated to start in January 2025 and complete in August 2030. |
| Zolk et al., 2022  (DRKS00015084) | Cardiovascular Health Status And Genetic Risk In Survivors of Childhood Neuroblastoma and Nephroblastoma Treated With Doxorubicin: Protocol of the Pharmacogenetic Part of the LESS-Anthra Cross-Sectional Cohort Study | The study protocol states that “we are in the final phase of data collection” and “data analyses are yet to be completed”. Genotype frequencies were reported but not analysed with case/control status. LYFW contacted authors; no reply received. |
| **Cost-effectiveness study** | | |
| Lapirow et al., 2021 | The Australia and New Zealand Cardio-Oncology Registry: evaluation of chemotherapy-related cardiotoxicity in a national cohort of paediatric cancer patients | Awaiting results of the cost-effectiveness analysis. LYFW contacted authors; no reply received. |

**Supplementary Table S23.** Characteristics of studies awaiting classification. Contains studies for which an inclusion or exclusion decision cannot be made due to unavailable information. All reasonable attempts were made to obtain the missing information.

| **Study** | **Title** | **Reason for awaiting classification** |
| --- | --- | --- |
| **Clinical effectiveness studies** | | |
| Aba et al., 2023 | CO12.1 - A case-control study to identify potential genetic biomarkers related to cardiac diseases occurrence in childhood cancer survivors | Conference abstract. Missing information on the clinical and genetic variables used in the prediction model. LYFW contacted authors; no reply received. |
| Hagleitner et al., 2011 | Relevance of germ-line genetic variations for treatment response in pediatric osteosarcoma patients | Conference abstract. Missing information on the names of ACT-associated SNPs. LYFW contacted authors; no reply received. |
| Hiyama et al., 2016 | Genetic risk facotrs [*sic*] of chemotherapy-related ototoxicity and cardiotoxicity in hepatoblastoma | Conference abstract. Missing information on the names of ACT-associated SNPs. LYFW contacted authors; no reply received. |
| Kutszegi et al., 2013 | Genetic risk factors of anthracycline-induced cardiotoxicity – relevant polymorphisms identified in enzymes and transporters of anthracycline pharmacokinetics | Conference abstract. Missing information on the number of cases and controls. LYFW contacted authors; a reply was received but it did not contain the required information. |
| Petrykey et al., 2023 | A genome-wide association study for doxorubicin-induced cardiomyopathy in childhood cancer survivors from the St. Jude lifetime cohort (SJLIFE) and the childhood cancer survivor (CCSS) studies | Conference abstract. Missing information on the number of cases and controls for the CCSS-European and SJLIFE-African cohorts. LYFW contacted authors; no reply received. |
| Toro et al., 2022 | Abstract 9749: Anthracicline-Induced Cardiotoxicity in Children. Pharmacogenetics and Clinical Study at a Tertiary Center. | Conference abstract. Missing information on the results of genetic associations. LYFW contacted authors; a reply was received but it did not contain the required information. |
| **Cost-effectiveness study** | | |
| Yan et al., 2020 | Economic evaluation of pharmacogenomic testing in pediatric oncology patients treated with anthracyclines | Conference abstract. Missing information on cost-effectiveness analysis results. LYFW contacted authors; no reply received. |

# Supplementary Appendix S4. Criteria for risk-of-bias and quality assessments

**Supplementary Table S24.** Criteria for the risk-of-bias and quality assessment of included studies.

| **ROBINS-E tool (Higgins et al., 2024) for risk-of-bias assessment of clinical effectiveness studies** | |
| --- | --- |
| **Bias domain** | **Criteria** |
| D1: Bias due to confounding | To assess whether important pre-specified confounding factors (Kremer et al., 2002; Lipshultz et al., 2007; Norton et al., 2021; Qiu et al., 2021) lead to bias sufficient to affect the direction of estimated effect or the ability to draw a conclusion from the study. Baseline confounders:   - age, gender/sex, race/ethnicity = minimum to be controlled for - cumulative anthracycline dose, type of cancer, type of anthracycline = important to be controlled for - pre-existing cardiovascular conditions (e.g. hypertension/coronary artery disease/diabetes/obesity) = important to be controlled for - use of other chemotherapy drugs, radiotherapy, dexrazoxane = important to be controlled for - length of follow-up = optional to be controlled for   We assessed if these confounders were controlled for in the analysis by regression, or controlled for in the study design by stratification, matching and recruitment.  Negative controls are defined as controls who did not receive the treatment (anthracyclines), and as a result, are expected to not develop the outcome (ACT, or simply cardiotoxicity in the absence of anthracyclines). They are usually used as an alternative analysis to identify sources of uncontrolled confounding. If controls who did not receive anthracycline develops cardiotoxicity, it may suggest there are other confounders contributing to this cardiotoxicity. Nonetheless, in this review, we excluded studies where participants were not receiving anthracyclines, thus the included studies are unlikely to have used negative controls.  Since the exposure (genetic variant) does not change over time, there were no repeated measurements of the exposure. Only baseline confounding, but not time-varying confounding, needs to be addressed. Therefore, we used the *Domain 1, Variant (a)* model in ROBINS-E for all included studies. |
| D2: Bias arising from measurement of the exposure | To assess whether the exposure status (genotype status) was adequately measured and classified using the chosen DNA-based genetic technique. We also assessed if any quality control measures (e.g. filtering, call rate, minor allele frequency [MAF], test for compliance with Hardy-Weinberg equilibrium [HWE]), were used for genotyping. In all studies, the single measurement of exposure (DNA genotyping) is sufficient to characterise the level and pattern of the exposure (genetic variant).  Since the exposure (genetic variant) was measured at a single point in time, there were no repeated measurements of the exposure. Therefore, we used the *Domain 2, Variant (a)* model in ROBINS-E for all included studies. |
| D3: Bias in selection of participants into the study (or into the analysis) | To assess whether participant selection is related to their outcome status (ACT) and exposure status (genotype status), i.e. selection bias.  The effect of genetic variants remains constant throughout life and the resulting ACT arises only after anthracycline use. Therefore, selecting participants *after* anthracycline use, instead of at the start of exposure, is more suitable and unlikely to introduce selection bias. |
| D4: Bias due to post-exposure interventions | To assess whether post-exposure interventions were given and affected the true effect of exposure.  Post-exposure interventions are defined as interventions which are given due to exposure status and may affect the true effect of exposure. Examples include gene therapy. |
| D5: Bias due to missing data | To assess whether data on exposure status, outcome and confounding variables are complete for all participants.  If there were missing data, to distinguish between data “missing at random” (MAR) and “missing not at random” (MNAR). |
| D6: Bias arising from measurement of the outcome | To assess whether the outcome (ACT) was consistently and objectively measured across different exposure status (genotype status). |
| D7: Bias in selection of the reported result | To assess whether results, including the measurements of exposure, outcome, exposure-outcome relationship and different subgroups, were clearly and fully reported. |
| **Overall risk of bias** | Low risk of bias if all domains have *Low risk of bias*.  Some concerns if i) at least one domain has *Some concerns*, ii) but no domains have *High*  *risk of bias* or *Very high risk of bias*.  High risk of bias if i) at least one domain has *High risk of bias*, ii) but no domains have *Very*  *high risk of bias.*  Very high risk of bias if at least one domain has *Very high risk of bias.* |
| **Drummond checklist (Drummond et al., 2015) for quality assessment of cost-effectiveness studies** | |
| 1. Was a well-defined question posed in answerable form? | Did the study comprehensively analyse the costs, effects, comparison of alternatives, analysis perspective, decision-making context, and patient population definitions? |
| 2. Was a comprehensive description of the competing alternatives given? | Were any relevant alternatives, e.g. a “do nothing” option, considered comprehensively for all patient subgroups? |
| 3. Was the effectiveness of the programmes or services established? | Was the effectiveness of this study determined through a comprehensive evaluation combining randomised controlled clinical trials, systematic reviews of clinical studies and consideration of observational data or assumptions, with adherence to trial protocols reflecting real-world scenarios and clear methodologies for data collection and analysis? |
| 4. Were all the important and relevant costs and consequences for each alternative identified? | Was the range of perspectives and costs adequately comprehensive for the research question, encompassing various relevant viewpoints including those of patients, third-party payers and other pertinent considerations, such as both capital and operating costs? |
| 5. Were costs and consequences measured accurately in appropriate physical units prior to valuation? | Were the sources of resource utilisation described, justified, and did the study address any items omitted from measurement?  Were any difficulties in measurement due to special circumstances, e.g. joint resource use, appropriately managed within the analysis? |
| 6. Were costs and consequences valued credibly? | Were all sources of values clearly identified, e.g. market values, patient or client preferences, policy-makers’ views and health professionals’ judgements?  Were market values used for changes in resources gained or depleted?  In cases where market values were not available (such as volunteer labour) or did not reflect actual values (like clinic space donated at a reduced rate), were adjustments made to approximate market values?  Was the valuation of consequences appropriate for the question posed, ensuring selection of the right analysis type (cost-effectiveness, cost-benefit)? |
| 7. Were costs and consequences adjusted for differential timing? | Was there a discounting of future costs and consequences to their present values in the study, and was there a rationale provided for the choice of discount rates utilised? |
| 8. Was an incremental analysis of costs and consequences of alternatives performed? | Were the incremental costs of one alternative compared to another in relation to the incremental effects, benefits, or utilities generated? |
| 9. Was uncertainty in the estimates of costs and consequences adequately characterised? | Were appropriate statistical analyses performed on patient-level data for costs or consequences, and was justification provided for the types and ranges of values used in sensitivity analyses?  Did the study’s conclusions consider the uncertainty in results, as quantified by statistical and/or sensitivity analyses, and did it recognise patient population heterogeneity by presenting results for relevant subgroups? |
| 10. Did the presentation and discussion of study results include all issues of concern to users? | Were conclusions based on an overall index or ratio of costs to consequences, and if so, was this index interpreted thoughtfully or mechanically?  Were the results compared with those of other investigations into the same question, with allowances made for potential differences in methodology?  Did the study discuss the generalisability of its results to other settings and patient/client groups, and did it consider other important factors in the decision-making process, such as the distribution of costs and consequences or relevant ethical issues?  Did the study address implementation issues, such as the feasibility of adopting the preferred program within existing constraints and the potential redeployment of freed resources to other worthwhile programs?  Were the implications of uncertainty for decision-making, including the need for future research, explored within the study? |

# Supplementary Appendix S5. Sensitivity analyses of conference abstracts and theses


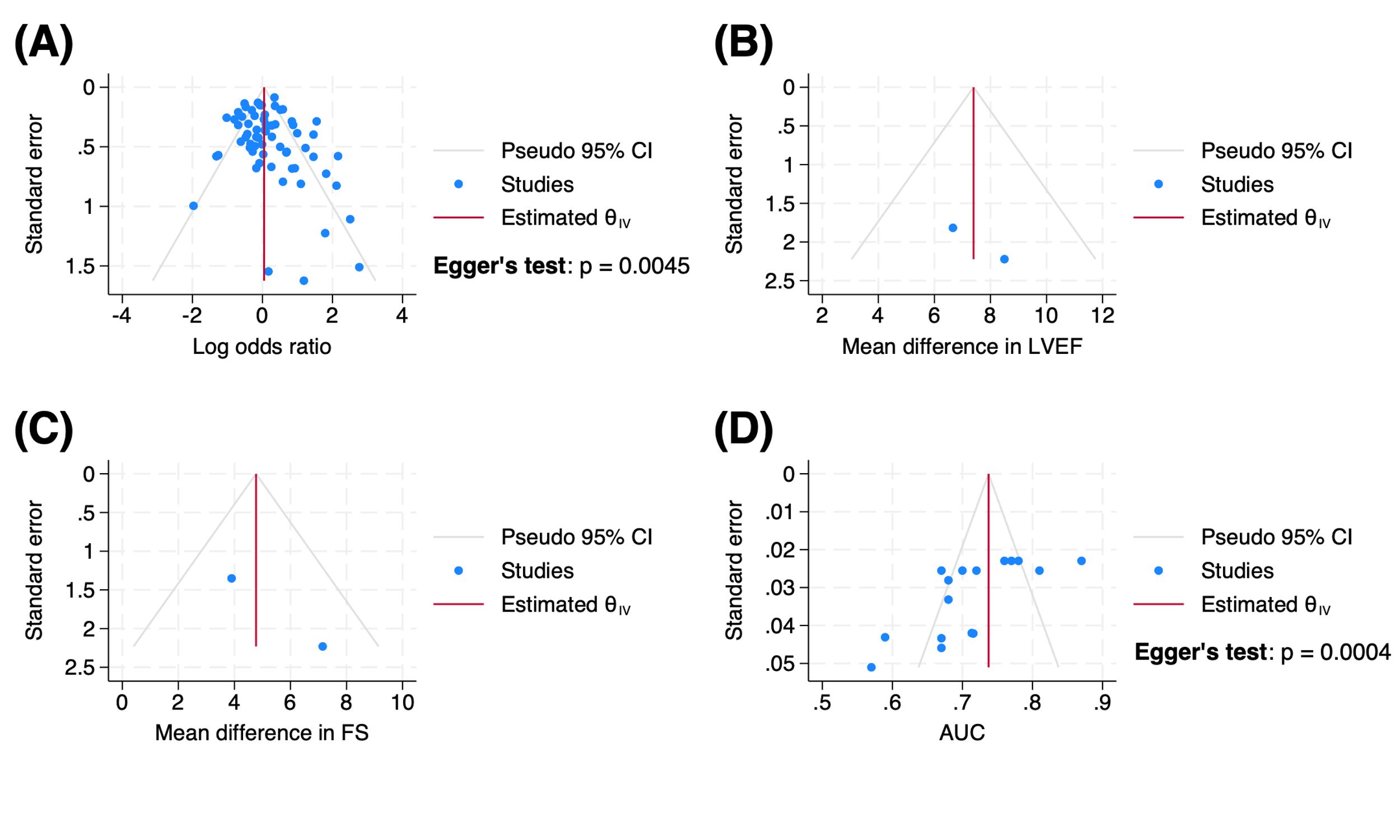


**Supplementary Figure S1.** Funnel plots with inclusion of conference abstracts and theses of standard error by **(A)** log odds ratio, **(B)** mean difference in left ventricular ejection fraction, **(C)** mean difference in fractional shortening and **(D)** area under the receiver operating characteristic curve in the body of evidence at outcome level. Wang et al., 2019 was excluded from Supplementary Figure S1D as it did not report 95% confidence interval or standard error for AUC. Abbreviations: AUC, area under the receiver operating characteristic curve; FS, fractional shortening; LVEF, left ventricular ejection fraction.


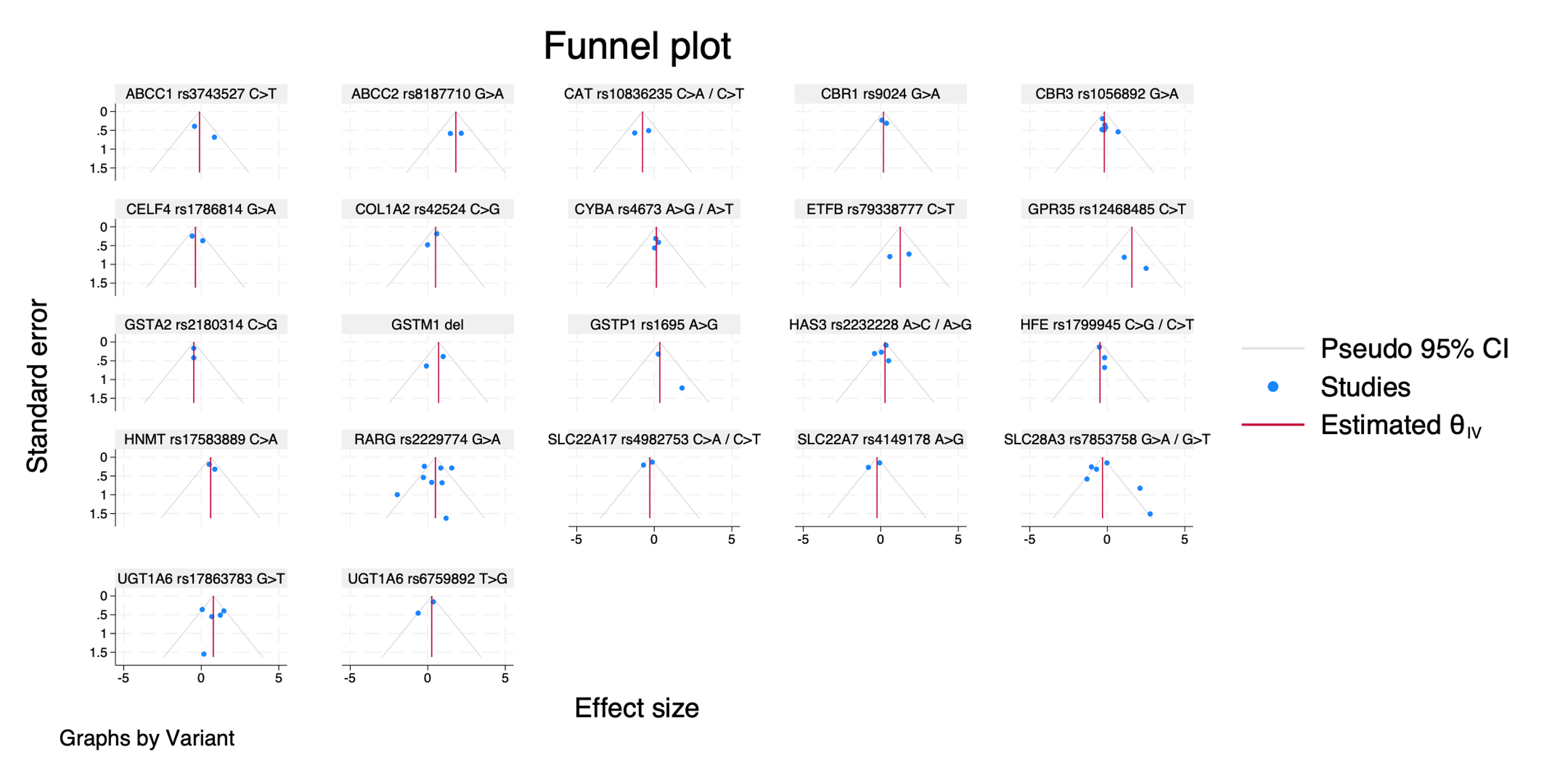


Log odds ratio


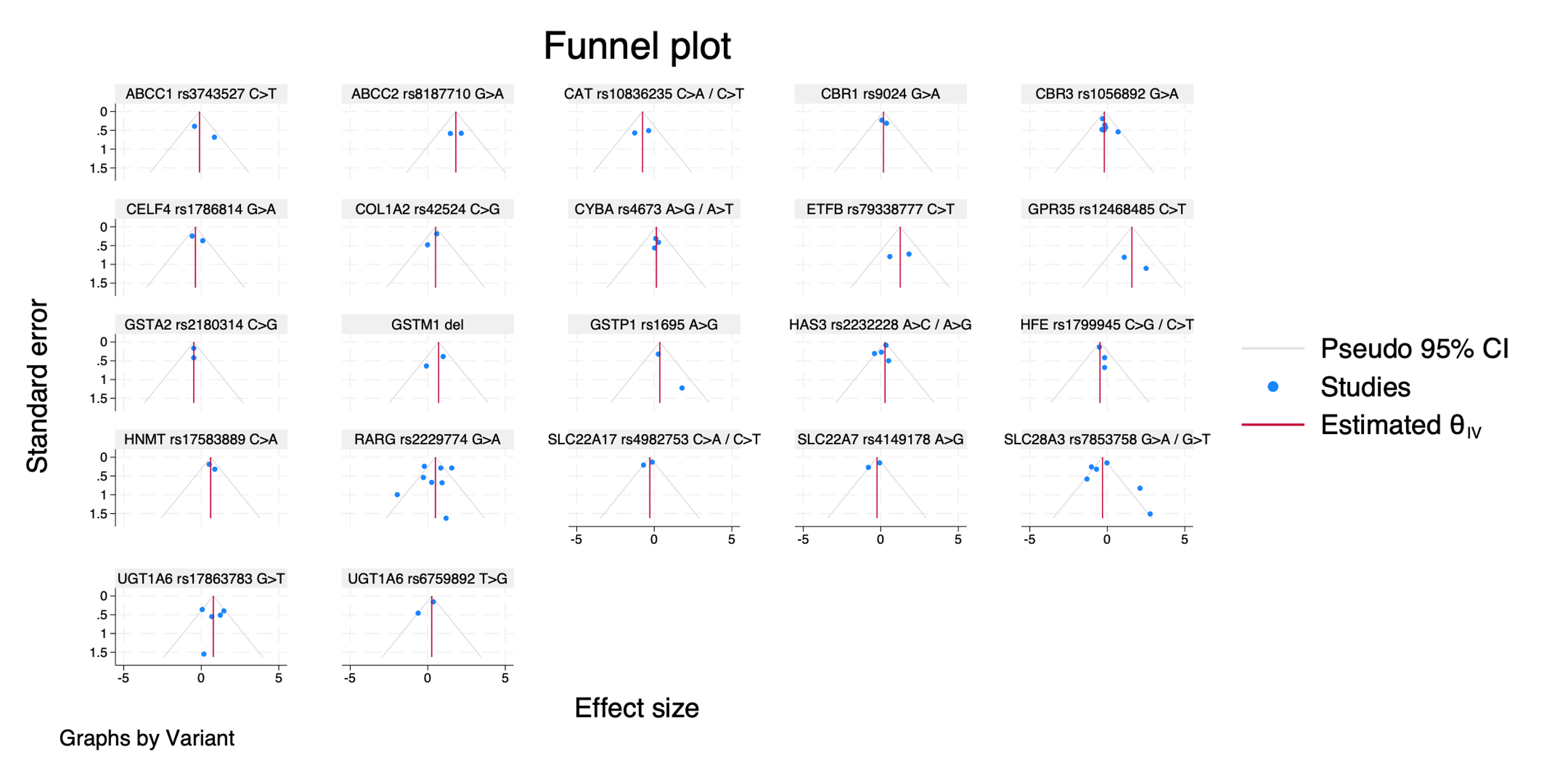


**Supplementary Figure S2.** Funnel plots with inclusion of conference abstracts and theses of standard error by log odds ratio. Subgroup analysis by variant was performed. After inclusion of the conference abstract Giljeviae et al., 2010, *GSTP1* rs1695 was assessed in two studies and thus eligible for meta-analysis.


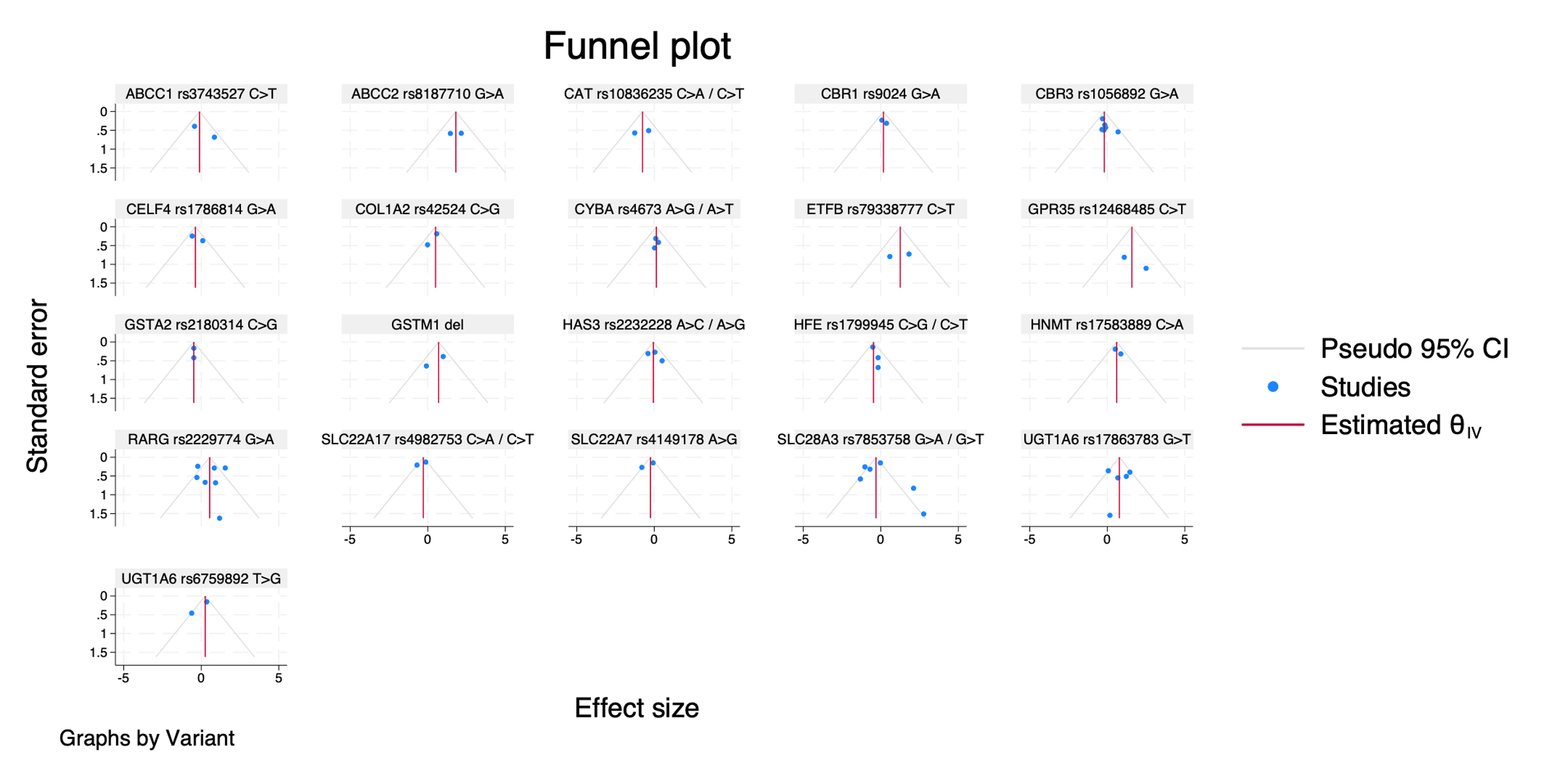


Log odds ratio


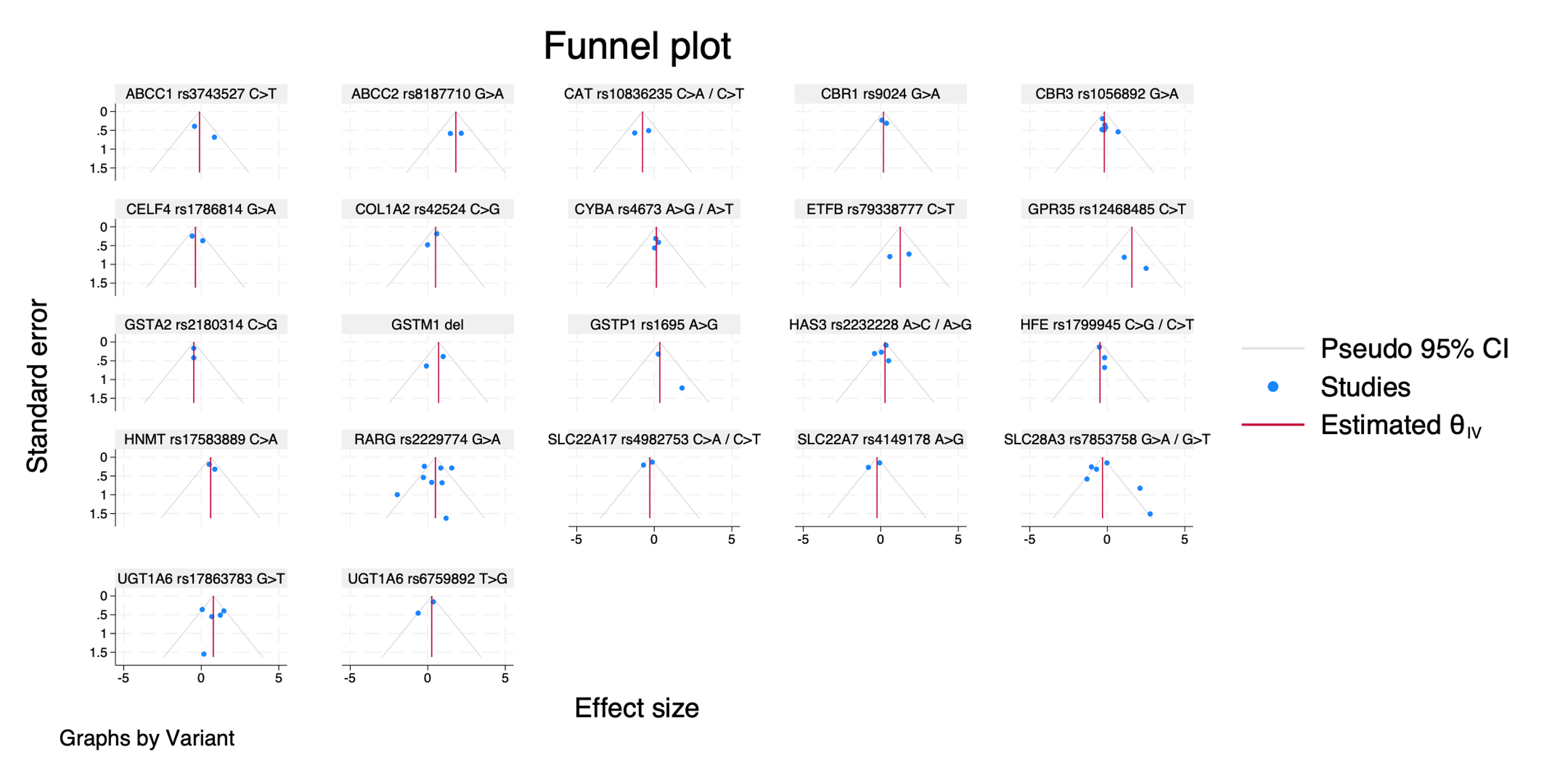


**Supplementary Figure S3.** Funnel plots *without* inclusion of conference abstracts and theses of standard error by log odds ratio. Subgroup analysis by variant was performed.


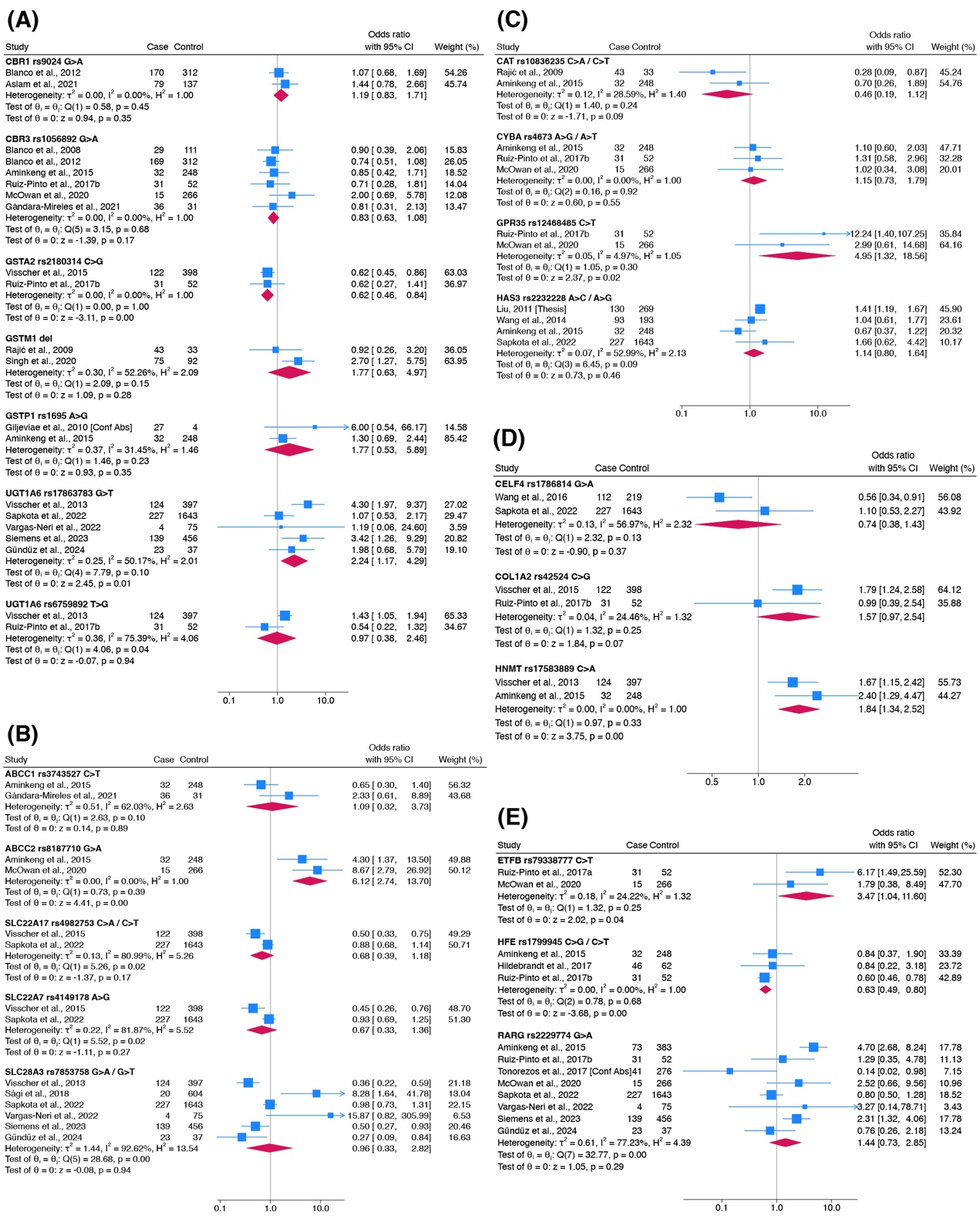


**Supplementary Figure S4.** Forest plots with inclusion of conference abstracts and theses showing the association of genetic variants responsible for **(A)** anthracycline metabolism, **(B)** anthracycline transport, **(C)** oxidative stress capacity, **(D)** contractility and **(E)** other functions with ACT occurrence. Other functions include mitochondrial function (*ETFB*), iron homeostasis (*HFE*) and DNA damage (*RARG*). Haldane-Anscombe correction was applied to zero-cell counts in Vargas-Neri et al., 2022. After inclusion of the conference abstract by Giljeviae et al., 2010, *GSTP1* rs1695 was assessed in two studies and thus became eligible for meta-analysis. Abbreviation: Conf Abs, conference abstract.

# References

Aba, N., Belhechmi, S., Fresneau, B., El-Fayech, C., Rubino, C., Allodji, R., et al. (2023). CO12.1 - A case-control study to identify potential genetic biomarkers related to cardiac diseases occurrence in childhood cancer survivors. *Revue d'Épidémiologie et de Santé Publique* 71. doi: 10.1016/j.respe.2023.101634.

Aminkeng, F., Bhavsar, A.P., Visscher, H., Rassekh, S.R., Li, Y., Lee, J.W., et al. (2015). A coding variant in RARG confers susceptibility to anthracycline-induced cardiotoxicity in childhood cancer. *Nature Genetics* 47(9)**,** 1079-1084. doi: 10.1038/ng.3374.

Aminkeng, F., Ross, C.J.D., Rassekh, S.R., Hwang, S., Rieder, M.J., Bhavsar, A.P., et al. (2016). Recommendations for genetic testing to reduce the incidence of anthracycline‐induced cardiotoxicity. *British Journal of Clinical Pharmacology* 82(3)**,** 683-695. doi: 10.1111/bcp.13008.

Armenian, S.H., Ding, Y., Mills, G., Sun, C., Venkataraman, K., Wong, F.L., et al. (2013). Genetic susceptibility to anthracycline‐related congestive heart failure in survivors of haematopoietic cell transplantation. *British Journal of Haematology* 163(2)**,** 205-213. doi: 10.1111/bjh.12516.

Aslam, S., Ameer, S., Shabana, N.A., and Ahmed, M. (2021). Pharmacogenetics of induction therapy-related toxicities in childhood acute lymphoblastic leukemia patients treated with UKALL 2003 protocol. *Scientific Reports* 11(1). doi: 10.1038/s41598-021-03208-9.

Ater, J., Thompson, K., and Hildebrandt, M. (2016). PD-061 Relationships among Hypertension Susceptibility Loci, Hypertension, and Late Anthracycline-Related Cardiotoxicity in Long-Term Childhood Cancer Survivors. *48th Congress of the International Society of Paediatric Oncology (SIOP)* 63(S3)**,** S5-S321. doi: 10.1002/pbc.26233.

Bhavsar, A.P., Rassekh, S.R., Aminkeng, F., Li, Y., Gunaretnam, E., Rieder, M.J., et al. (2016). O-074 Pharmacogenomic Strategies for the Prevention of Anthracycline-Induced Heart Failure: Validation of a Genetic Association with a Non-Synonymous Variant in RARG. *48th Congress of the International Society of Paediatric Oncology (SIOP)* 63(S3)**,** S5-S321. doi: 10.1002/pbc.26233.

Blanco, J.G., Leisenring, W.M., Gonzalez‐Covarrubias, V.M., Kawashima, T.I., Davies, S.M., Relling, M.V., et al. (2008). Genetic polymorphisms in the carbonyl reductase 3 gene CBR3 and the NAD(P)H:quinone oxidoreductase 1 gene NQO1 in patients who developed anthracycline‐related congestive heart failure after childhood cancer. *Cancer* 112(12)**,** 2789-2795. doi: 10.1002/cncr.23534.

Blanco, J.G., Sun, C.-L., Landier, W., Chen, L., Esparza-Duran, D., Leisenring, W., et al. (2012). Anthracycline-Related Cardiomyopathy After Childhood Cancer: Role of Polymorphisms in Carbonyl Reductase Genes—A Report From the Children's Oncology Group. *Journal of Clinical Oncology* 30(13)**,** 1415-1421. doi: 10.1200/jco.2011.34.8987.

Boies, L.N. (2021). *Identification of Anthracycline-Induced Cardiotoxic Susceptible Loci in Childhood Cancer Survivors.* M.P.H., The University of Texas School of Public Health.

Chaix, M., Lafreniere-Roula, M., Akinrinade, O., Yao, R., Miron, A., Lam, E., et al. (2018). Abstract 12821: Genomic Factors Associated With Anthracycline Cardiotoxicity in Pediatric Cancer Survivors. *2018 American Heart Association Scientific Sessions* 138(Suppl_1)**,** A12821.

Chaix, M.-A., Parmar, N., Kinnear, C., Lafreniere-Roula, M., Akinrinade, O., Yao, R., et al. (2020). Machine Learning Identifies Clinical and Genetic Factors Associated With Anthracycline Cardiotoxicity in Pediatric Cancer Survivors. *JACC: CardioOncology* 2(5)**,** 690-706. doi: 10.1016/j.jaccao.2020.11.004.

Conyers, R., Devaraja, S., and Elliott, D. (2017). Systematic review of pharmacogenomics and adverse drug reactions in paediatric oncology patients. *Pediatric Blood & Cancer* 65(4). doi: 10.1002/pbc.26937.

Dionne, F., Aminkeng, F., Bhavsar, A.P., Groeneweg, G., Smith, A., Visscher, H., et al. (2017). An initial health economic evaluation of pharmacogenomic testing in patients treated for childhood cancer with anthracyclines. *Pediatric Blood & Cancer* 65(3). doi: 10.1002/pbc.26887.

Drummond, M., Sculpher, M.J., Claxton, K., Stoddart, G.L., Torrance, G.W., and Ebscohost (2015). "Methods for the economic evaluation of health care programmes", in: *Oxford medical publications Methods for the economic evaluation of health care programmes.* Fourth edition. ed. (Oxford, England: Oxford University Press).

Ehrhardt, M.J., Leerink, J.M., Mulder, R.L., Mavinkurve-Groothuis, A., Kok, W., Nohria, A., et al. (2023). Systematic review and updated recommendations for cardiomyopathy surveillance for survivors of childhood, adolescent, and young adult cancer from the International Late Effects of Childhood Cancer Guideline Harmonization Group. *The Lancet Oncology* 24(3)**,** e108-e120. doi: 10.1016/s1470-2045(23)00012-8.

Ehrhardt, M.J., Ward, Z.J., Liu, Q., Chaudhry, A., Nohria, A., Border, W., et al. (2020). Cost-Effectiveness of the International Late Effects of Childhood Cancer Guideline Harmonization Group Screening Guidelines to Prevent Heart Failure in Survivors of Childhood Cancer. *Journal of Clinical Oncology* 38(33)**,** 3851-3862. doi: 10.1200/jco.20.00418.

Gándara-Mireles, J.A., Lares-Asseff, I., Reyes Espinoza, E.A., Blanco, J.G., González Font, A.E., Córdova Hurtado, L.P., et al. (2021). Association of genetic polymorphisms NCF4 rs1883112, CBR3 rs1056892, and ABCC1 rs3743527 with the cardiotoxic effects of doxorubicin in children with acute lymphoblastic leukemia. *Pharmacogenetics and Genomics* 31(5)**,** 108-115. doi: 10.1097/fpc.0000000000000428.

Gándara-Mireles, J.A., Lares-Asseff, I., Reyes Espinoza, E.A., Fierro, I.V., Castañeda, V.L., Cordova Hurtado, L.P., et al. (2022). Impact of single-nucleotide variants and nutritional status on population pharmacokinetics of Doxorubicin, and its effect on cardiotoxicity in children with leukemia. *Journal of Oncology Pharmacy Practice* 29(6)**,** 1290-1305. doi: 10.1177/10781552221117810.

Garcia-Pavia, P., Kim, Y., Restrepo-Cordoba, M.A., Lunde, I.G., Wakimoto, H., Smith, A.M., et al. (2019). Genetic Variants Associated With Cancer Therapy–Induced Cardiomyopathy. *Circulation* 140(1)**,** 31-41. doi: 10.1161/circulationaha.118.037934.

Giljeviae, J.S., Ivkovic, T.C., Bonevski, A., Krleza, J.L., Jakovljevic, G., and Kapitanovic, S. (2010). GSTP1 ILE105VAL polymorphism and doxorubicin induced toxicity in children with solid tumors. *42nd Congress of the International Society of Pediatric Oncology, SIOP 2010* 55(5)**,** 941.

Gündüz, A., Duman, D., Başbinar, Y., Taşdelen, B., Küpeli, S., and Karpuz, D. (2024). The Role of RARG rs2229774, SLC28A3 rs7853758, and UGT1A6*4 rs17863783 Single-nucleotide Polymorphisms in the Doxorubicin-induced Cardiotoxicity in Solid Childhood Tumors. *Journal of Pediatric Hematology/Oncology* 46(1)**,** e65-e70. doi: 10.1097/mph.0000000000002768.

Güntürkün, F., Akbilgic, O., Davis, R.L., Armstrong, G.T., Howell, R.M., Jefferies, J.L., et al. (2021). Artificial Intelligence–Assisted Prediction of Late-Onset Cardiomyopathy Among Childhood Cancer Survivors. *JCO Clinical Cancer Informatics* (5)**,** 459-468. doi: 10.1200/cci.20.00176.

Hagleitner, M., Hoogerbrugge, P., Schreuder, B.W.B., Flucke, U.E., Coenen, M.J., and Te Loo, D.M.W. (2011). Relevance of germ-line genetic variations for treatment response in pediatric osteosarcoma patients. *Journal of Clinical Oncology* 29(15_suppl)**,** 9518-9518. doi: 10.1200/jco.2011.29.15_suppl.9518.

Hellmann, F., Völler, S., Krischke, M., Jamieson, D., André, N., Bisogno, G., et al. (2020). Genetic Polymorphisms Affecting Cardiac Biomarker Concentrations in Children with Cancer: an Analysis from the “European Paediatric Oncology Off-patents Medicines Consortium” (EPOC) Trial. *European Journal of Drug Metabolism and Pharmacokinetics* 45(3)**,** 413-422. doi: 10.1007/s13318-019-00592-6.

Hertz, D.L., Caram, M.V., Kidwell, K.M., Thibert, J.N., Gersch, C., Seewald, N.J., et al. (2016). Evidence for association of SNPs in ABCB1 and CBR3, but not RAC2, NCF4, SLC28A3 or TOP2B, with chronic cardiotoxicity in a cohort of breast cancer patients treated with anthracyclines. *Pharmacogenomics* 17(3)**,** 231-240. doi: 10.2217/pgs.15.162.

Higgins, J.P.T., Morgan, R.L., Rooney, A.A., Taylor, K.W., Thayer, K.A., Silva, R.A., et al. (2024). A tool to assess risk of bias in non-randomized follow-up studies of exposure effects (ROBINS-E). *Environment International* 186. doi: 10.1016/j.envint.2024.108602.

Hildebrandt, M.A.T., Reyes, M., Wu, X., Pu, X., Thompson, K.A., Ma, J., et al. (2017). Hypertension Susceptibility Loci are Associated with Anthracycline-related Cardiotoxicity in Long-term Childhood Cancer Survivors. *Scientific Reports* 7(1). doi: 10.1038/s41598-017-09517-2.

Hiyama, E., Hishiki, T., Ida, K., Watanabe, K., Oue, T., Yano, M., et al. (2016). Genetic risk facotrs of chemotherapy-related ototoxicity and cardiotoxicity in hepatoblastoma. *48th Congress of the International Society of Paediatric Oncology, SIOP 2016* 63(Supplement 3)**,** S165.

Huang, Z., Wang, J., Qian, J., Li, Y., Xu, Z., Chen, M., et al. (2017). Effects of cytochrome P450 family 3 subfamily A member 5 gene polymorphisms on daunorubicin metabolism and adverse reactions in patients with acute leukemia. *Molecular Medicine Reports* 15(6)**,** 3493-3498. doi: 10.3892/mmr.2017.6470.

Hurkmans, E.G.E., Brand, A.C.A.M., Verdonschot, J.A.J., te Loo, D.M.W.M., and Coenen, M.J.H. (2022). Pharmacogenetics of chemotherapy treatment response and -toxicities in patients with osteosarcoma: a systematic review. *BMC Cancer* 22(1). doi: 10.1186/s12885-022-10434-5.

Kennedy, R., and Boughey, J. (2013). Management of Pediatric and Adolescent Breast Masses. *Seminars in Plastic Surgery* 27(01)**,** 019-022. doi: 10.1055/s-0033-1343991.

Kissoon, T. (2019). Using Pharmacogenomics Testing to Optimize Care in Children with Acute Hematologic Malignancies. *Blood* 134(Supplement_1)**,** 5062-5062. doi: 10.1182/blood-2019-129511.

Kitagawa, K., Kawada, K., Morita, S., Inada, M., Mitsuma, A., Sawaki, M., et al. (2012). Prospective evaluation of corrected QT intervals and arrhythmias after exposure to epirubicin, cyclophosphamide, and 5-fluorouracil in women with breast cancer. *Annals of Oncology* 23(3)**,** 743-747. doi: 10.1093/annonc/mdr296.

Krajinovic, M., Elbared, J., Drouin, S., Bertout, L., Rezgui, A., Ansari, M., et al. (2015). Polymorphisms of ABCC5 and NOS3 genes influence doxorubicin cardiotoxicity in survivors of childhood acute lymphoblastic leukemia. *The Pharmacogenomics Journal* 16(6)**,** 530-535. doi: 10.1038/tpj.2015.63.

Kremer, L.C.M., van der Pal, H.J.H., Offringa, M., van Dalen, E.C., and Voûte, P.A. (2002). Frequency and risk factors of subclinical cardiotoxicity after anthracycline therapy in children: a systematic review. *Annals of Oncology* 13(6)**,** 819-829. doi: 10.1093/annonc/mdf167.

Kutszegi, N., Semsei, A.F., Lautner-Csorba, O., Hegyi, M., Szalai, C., Kovacs, G.T., et al. (2013). Genetic risk factors of anthracycline-induced cardiotoxicity – relevant polymorphisms identified in enzymes and transporters of anthracycline pharmacokinetics. *2nd ESPT Conference "Pharmacogenomics: From Cell to Clinic"* 28(3)**,** A14.

Lapirow, D., La Gerche, A., Toro, C., Masango, E., Costello, B., Porello, E., et al. (2021). The Australia and New Zealand Cardio‐Oncology Registry: evaluation of chemotherapy‐related cardiotoxicity in a national cohort of paediatric cancer patients. *Internal Medicine Journal* 51(2)**,** 229-234. doi: 10.1111/imj.14719.

Lares-Asseff, I., Gandara-Mireles, J.A., Reyes Espinoza, E.A., Blanco, J.G., Gonzalez Font, A.E., Cordova Hurtado, L.P., et al. (2021). Association of Genetic Polymorphisms NCF4 rs1883112, CBR3 rs1056892, and ABCC1 rs3743527 with the Cardiotoxic Effects of Doxorubicin. *18th Latin American Congress of Genetics, 54th Annual Meeting of the Chilean Society of Genetics, 49th Argentine Congress of Genetics, 8th Congress of the Uruguayan Society of Genetics, 1st Paraguayan Congress of Genetics and 5th Latin American Congress of Human Genetics* 32(SUPPL 1)**,** 18.

Leger, K.J., Leonard, D., Nielson, D., de Lemos, J.A., Mammen, P.P.A., and Winick, N.J. (2017). Circulating microRNAs: Potential Markers of Cardiotoxicity in Children and Young Adults Treated With Anthracycline Chemotherapy. *Journal of the American Heart Association* 6(4). doi: 10.1161/jaha.116.004653.

Leong, S.L., Chaiyakunapruk, N., and Lee, S.W.H. (2017). Candidate Gene Association Studies of Anthracycline-induced Cardiotoxicity: A Systematic Review and Meta-analysis. *Scientific Reports* 7(1). doi: 10.1038/s41598-017-00075-1.

Linschoten, M., Teske, A.J., Cramer, M.J., van der Wall, E., and Asselbergs, F.W. (2018). Chemotherapy-Related Cardiac Dysfunction: A Systematic Review of Genetic Variants Modulating Individual Risk. *Circulation: Genomic and Precision Medicine* 11(1). doi: 10.1161/circgen.117.001753.

Lipshultz, S.E., Alvarez, J.A., and Scully, R.E. (2007). Anthracycline associated cardiotoxicity in survivors of childhood cancer. *Heart* 94(4)**,** 525-533. doi: 10.1136/hrt.2007.136093.

Lipshultz, S.E., Lipsitz, S.R., Kutok, J.L., Miller, T.L., Colan, S.D., Neuberg, D.S., et al. (2013). Impact of hemochromatosis gene mutations on cardiac status in doxorubicin‐treated survivors of childhood high‐risk leukemia. *Cancer* 119(19)**,** 3555-3562. doi: 10.1002/cncr.28256.

Liu, W. (2011). *Gene-Environmental Interaction Assessment in Genome Wide Association Study.* M.Sc., University of Alberta (Canada).

Loucks, C.M., Yan, K., Tanoshima, R., Ross, C.J.D., Rassekh, S.R., and Carleton, B.C. (2021). Pharmacogenetic testing to guide therapeutic decision‐making and improve outcomes for children undergoing anthracycline‐based chemotherapy. *Basic & Clinical Pharmacology & Toxicology* 130(S1)**,** 95-99. doi: 10.1111/bcpt.13593.

Lubieniecka, J.M., Graham, J., Heffner, D., Mottus, R., Reid, R., Hogge, D., et al. (2013). A discovery study of daunorubicin induced cardiotoxicity in a sample of acute myeloid leukemia patients prioritizes P450 oxidoreductase polymorphisms as a potential risk factor. *Frontiers in Genetics* 4. doi: 10.3389/fgene.2013.00231.

Lubieniecka, J.M., Liu, J., Heffner, D., Graham, J., Reid, R., Hogge, D., et al. (2012). Single-Nucleotide Polymorphisms in Aldo-Keto and Carbonyl Reductase Genes Are Not Associated with Acute Cardiotoxicity after Daunorubicin Chemotherapy. *Cancer Epidemiology, Biomarkers & Prevention* 21(11)**,** 2118-2120. doi: 10.1158/1055-9965.Epi-12-1037.

Magdy, T., Jouni, M., Kuo, H.-H., Weddle, C.J., Lyra-Leite, D., Fonoudi, H., et al. (2022). Identification of Drug Transporter Genomic Variants and Inhibitors That Protect Against Doxorubicin-Induced Cardiotoxicity. *Circulation* 145(4)**,** 279-294. doi: 10.1161/circulationaha.121.055801.

McOwan, T.N., Craig, L.A., Tripdayonis, A., Karavendzas, K., Cheung, M.M., Porrello, E.R., et al. (2020). Evaluating anthracycline cardiotoxicity associated single nucleotide polymorphisms in a paediatric cohort with early onset cardiomyopathy. *Cardio-Oncology* 6(1). doi: 10.1186/s40959-020-00060-0.

Norton, N., Weil, R.M., and Advani, P.P. (2021). Inter-Individual Variation and Cardioprotection in Anthracycline-Induced Heart Failure. *Journal of Clinical Medicine* 10(18). doi: 10.3390/jcm10184079.

Oatmen, K.E., Toro-Salazar, O.H., Hauser, K., Zellars, K.N., Mason, K.C., Hor, K., et al. (2018). Identification of a novel microRNA profile in pediatric patients with cancer treated with anthracycline chemotherapy. *American Journal of Physiology-Heart and Circulatory Physiology* 315(5)**,** H1443-H1452. doi: 10.1152/ajpheart.00252.2018.

Osterweil, N. (2010). *Genes raise risk of anthracycline-related cardiotoxicity* [Online]. United States: Oncology Report. Available: <http://ovidsp.ovid.com/ovidweb.cgi?T=JS&PAGE=reference&D=emed11&NEWS=N&AN=359633744> [Accessed 26 February 2024].

Petrykey, K., Rezgui, A.M., Guern, M.L., Beaulieu, P., St-Onge, P., Drouin, S., et al. (2021). Genetic factors in treatment-related cardiovascular complications in survivors of childhood acute lymphoblastic leukemia. *Pharmacogenomics* 22(14)**,** 885-901. doi: 10.2217/pgs-2021-0067.

Petrykey, K., Wang, H., Im, C., Dixon, S.B., Ehrhardt, M.J., Mulrooney, D.A., et al. (2023). A genome-wide association study for doxorubicin-induced cardiomyopathy in childhood cancer survivors from the St. Jude lifetime cohort (SJLIFE) and the childhood cancer survivor (CCSS) studies. *Journal of Clinical Oncology* 41(16_suppl)**,** 12088-12088. doi: 10.1200/JCO.2023.41.16_suppl.12088.

Qiu, S., Zhou, T., Qiu, B., Zhang, Y., Zhou, Y., Yu, H., et al. (2021). Risk Factors for Anthracycline-Induced Cardiotoxicity. *Frontiers in Cardiovascular Medicine* 8. doi: 10.3389/fcvm.2021.736854.

Ragab, S.M., El-Hawy, M.A., El-Hefnawy, S.M., El –Deeb, H.M.A., Elfalah, A.S., and Mahmoud, A.A. (2024). CELF4 (rs1786814) gene polymorphism and speckle-tracking Echocardiography for cardiovascular complications in childhood cancer survivors. *Pediatric Research*. doi: 10.1038/s41390-024-03400-3.

Rajić, V., Aplenc, R., Debeljak, M., Prestor, V.V., Karas-Kuželicki, N., MlinariČ-RašČan, I., et al. (2009). Influence of the polymorphism in candidate genes on late cardiac damage in patients treated due to acute leukemia in childhood. *Leukemia & Lymphoma* 50(10)**,** 1693-1698. doi: 10.1080/10428190903177212.

Reichwagen, A., Ziepert, M., Kreuz, M., Gödtel-Armbrust, U., Rixecker, T., Poeschel, V., et al. (2015). Association of NADPH oxidase polymorphisms with anthracycline-induced cardiotoxicity in the RICOVER-60 trial of patients with aggressive CD20+ B-cell lymphoma. *Pharmacogenomics* 16(4)**,** 361-372. doi: 10.2217/pgs.14.179.

Reinbolt, R.E., Patel, R., Pan, X., Timmers, C.D., Pilarski, R., Shapiro, C.L., et al. (2015). Risk factors for anthracycline-associated cardiotoxicity. *Supportive Care in Cancer* 24(5)**,** 2173-2180. doi: 10.1007/s00520-015-3008-y.

Rossi, D., Rasi, S., Franceschetti, S., Capello, D., Castelli, A., De Paoli, L., et al. (2009). Analysis of the host pharmacogenetic background for prediction of outcome and toxicity in diffuse large B-cell lymphoma treated with R-CHOP21. *Leukemia* 23(6)**,** 1118-1126. doi: 10.1038/leu.2008.398.

Ruiz-Pinto, S., Pita, G., Martín, M., Alonso-Gordoa, T., Barnes, D.R., Alonso, M.R., et al. (2017a). Exome array analysis identifies ETFB as a novel susceptibility gene for anthracycline-induced cardiotoxicity in cancer patients. *Breast Cancer Research and Treatment* 167(1)**,** 249-256. doi: 10.1007/s10549-017-4497-9.

Ruiz-Pinto, S., Pita, G., Patiño-García, A., Alonso, J., Pérez-Martínez, A., Cartón, A.J., et al. (2017b). Exome array analysis identifies GPR35 as a novel susceptibility gene for anthracycline-induced cardiotoxicity in childhood cancer. *Pharmacogenetics and Genomics* 27(12)**,** 445-453. doi: 10.1097/fpc.0000000000000309.

Ruiz-Pinto, S., Pita, G., Patiño-García, A., García-Miguel, P., Pérez-Martínez, A., Cartón, A.J., et al. (2016). Exome Array Analysis Identifies New Loci And Low-Frequency Variants Associated With Anthracycline-Induced Cardiotoxicity. *7th Conference of the Spanish Pharmacogenetics and Pharmacogenomics Society, SEFF 2015* 31(1)**,** eA6.

Sachidanandam, K., Gayle, A.A., Robins, H.I., and Kolesar, J.M. (2012). Unexpected doxorubicin-mediated cardiotoxicity in sisters: Possible role of polymorphisms in histamine n-methyl transferase. *Journal of Oncology Pharmacy Practice* 19(3)**,** 269-272. doi: 10.1177/1078155212461022.

Sági, J.C., Egyed, B., Kelemen, A., Kutszegi, N., Hegyi, M., Gézsi, A., et al. (2018). Possible roles of genetic variations in chemotherapy related cardiotoxicity in pediatric acute lymphoblastic leukemia and osteosarcoma. *BMC Cancer* 18(1). doi: 10.1186/s12885-018-4629-6.

Salanci, B.V., Aksoy, H., Kiratli, P.Ö., Tülümen, E., Güler, N., Öksüzoglu, B., et al. (2013). The relationship between changes in functional cardiac parameters following anthracycline therapy and carbonyl reductase 3 and glutathione S transferase Pi polymorphisms. *Journal of Chemotherapy* 24(5)**,** 285-291. doi: 10.1179/1973947812y.0000000037.

Salanci, B.V., Tulumen, E., Aksoy, H., Okutucu, S., Kiratli, P.O., Oksuzoglu, B., et al. (2010). OP-046 The Relation between Functional Cardiac Parameters and Single Nucleotide Polmorphisms in Glutathione S Transferase P1 and Carbonyl Reductase3 Genes. *International Journal of Cardiology* 140. doi: 10.1016/s0167-5273(10)70048-6.

Salazar, O. (2024). *Approaches to Identify Early Biomarkers and Pathogenesis of Anthracycline Cardiotoxicity (NCT04036045).* [Online]. United States: ClinicalTrials.gov. Available: <https://clinicaltrials.gov/study/NCT04036045> [Accessed 26 February 2024].

Samosir, S.M., Utamayasa, I.K.A., Andarsini, M.R., Rahman, M.A., Ontoseno, T., Hidayat, T., et al. (2021). Risk Factors of Daunorubicine Induced Early Cardiotoxicity in Childhood Acute Lymphoblastic Leukemia: A Retrospective Study. *Asian Pacific Journal of Cancer Prevention* 22(5)**,** 1407-1412. doi: 10.31557/apjcp.2021.22.5.1407.

Sapkota, Y., Ehrhardt, M.J., Qin, N., Wang, Z., Liu, Q., Qiu, W., et al. (2022). A Novel Locus on 6p21.2 for Cancer Treatment–Induced Cardiac Dysfunction Among Childhood Cancer Survivors. *JNCI: Journal of the National Cancer Institute* 114(8)**,** 1109-1116. doi: 10.1093/jnci/djac115.

Sapkota, Y., Qin, N., Ehrhardt, M.J., Wang, Z., Chen, Y., Wilson, C.L., et al. (2021). Genetic Variants Associated with Therapy-Related Cardiomyopathy among Childhood Cancer Survivors of African Ancestry. *Cancer Research* 81(9)**,** 2556-2565. doi: 10.1158/0008-5472.Can-20-2675.

Scott, E., Drogemoller, B., Wright, G., Carleton, B., and Ross, C. (2020). Additional genetic risk factors for anthracycline-induced cardiotoxicity in pediatric cancer. *52nd Congress of the International Society of Paediatric Oncology, SIOP* 67(SUPPL 4)**,** no pagination.

Semsei, A.F. (2011). *Transzportfehérjék genetikai polimorfizmusainak szerepe akut limfoid leukémiában; farmakogenetikai vizsgálatok.* Ph.D., Semmelweis Egyetem (Hungary).

Semsei, A.F., Erdelyi, D.J., Ungvari, I., Csagoly, E., Hegyi, M.Z., Kiszel, P.S., et al. (2012). ABCC1 polymorphisms in anthracycline-induced cardiotoxicity in childhood acute lymphoblastic leukaemia. *Cell Biology International* 36(1)**,** 79-86. doi: <https://dx.doi.org/10.1042/CBI20110264>.

Sharafeldin, N., Zhou, L., Singh, P., Crossman, D.K., Wang, X., Hageman, L., et al. (2023). Gene-Level Analysis of Anthracycline-Induced Cardiomyopathy in Cancer Survivors. *JACC: CardioOncology* 5(6)**,** 807-818. doi: 10.1016/j.jaccao.2023.06.007.

Siemens, A., Rassekh, S.R., Ross, C.J.D., and Carleton, B.C. (2023). Development of a Dose-Adjusted Polygenic Risk Model for Anthracycline-Induced Cardiotoxicity. *Therapeutic Drug Monitoring* 45(3)**,** 337-344. doi: 10.1097/ftd.0000000000001077.

Singh, P., Crossman, D.K., Zhou, L., Wang, X., Sharafeldin, N., Hageman, L., et al. (2023a). Haptoglobin Gene Expression and Anthracycline-Related Cardiomyopathy in Childhood Cancer Survivors. *JACC: CardioOncology* 5(3)**,** 392-401. doi: 10.1016/j.jaccao.2022.09.009.

Singh, P., Shah, D.A., Jouni, M., Cejas, R.B., Crossman, D.K., Magdy, T., et al. (2023b). Altered Peripheral Blood Gene Expression in Childhood Cancer Survivors With Anthracycline‐Induced Cardiomyopathy – A COG‐ALTE03N1 Report. *Journal of the American Heart Association* 12(19). doi: 10.1161/jaha.123.029954.

Singh, P., Wang, X., Hageman, L., Chen, Y., Magdy, T., Landier, W., et al. (2020). Association of GSTM1 null variant with anthracycline‐related cardiomyopathy after childhood cancer—A Children's Oncology Group ALTE03N1 report. *Cancer* 126(17)**,** 4051-4058. doi: 10.1002/cncr.32948.

Singh, P., Zhou, L., Shah, D.A., Cejas, R.B., Crossman, D.K., Jouni, M., et al. (2023c). Identification of novel hypermethylated or hypomethylated CpG sites and genes associated with anthracycline-induced cardiomyopathy. *Scientific Reports* 13(1). doi: 10.1038/s41598-023-39357-2.

Skitch, A., Mital, S., Mertens, L., Liu, P., Kantor, P., Grosse-Wortmann, L., et al. (2017). Novel approaches to the prediction, diagnosis and treatment of cardiac late effects in survivors of childhood cancer: a multi-centre observational study. *BMC Cancer* 17(1). doi: 10.1186/s12885-017-3505-0.

Svyatova, G., Boranbayeva, R., Berezina, G., Manzhuova, L., and Murtazaliyeva, A. (2023). Genes of Predisposition to Childhood Beta-Cell Acute Lymphoblastic Leukemia in the Kazakh Population. *Asian Pacific Journal of Cancer Prevention* 24(8)**,** 2653-2666. doi: 10.31557/apjcp.2023.24.8.2653.

Tonorezos, E.S., Joseph, V., Barnea, D., Villano, D., Satagopan, J., Friedman, D.N., et al. (2017). Protection from late-occurring anthracycline-related cardiotoxicity among childhood cancer survivors with a RARG coding variant. *Journal of Clinical Oncology* 35(5_suppl)**,** 130-130. doi: 10.1200/JCO.2017.35.5_suppl.130.

Toro, N.M., Iglecias, L.M.M., Blesa, C.C., Rey, M.d.M.R.V.d., Maldonado, E.U., Acosta, M.J.O., et al. (2022). Abstract 9749: Anthracicline-Induced Cardiotoxicity in Children. Pharmacogenetics and Clinical Study at a Tertiary Center. *Circulation* 146(Suppl_1). doi: 10.1161/circ.146.suppl_1.9749.

Tron, C., Verdier, M.C., Gandemer, V., Pertuisel, S., Bonneau-Lagacherie, J., Chappe, C., et al. (2021). Could pharmacogenetics testing explain anthracycline-induced cardiotoxicity sensitivity in hematologic pediatric patients? *Annual Meeting of French Society of Pharmacology and Therapeutics* 35(SUPPL 1)**,** 168-169.

Vargas-Neri, J.L., Carleton, B., Ross, C.J., Medeiros, M., Castañeda-Hernández, G., and Clark, P. (2022). Pharmacogenomic study of anthracycline-induced cardiotoxicity in Mexican pediatric patients. *Pharmacogenomics* 23(5)**,** 291-301. doi: 10.2217/pgs-2021-0144.

Vinodhini, M.T., Sneha, S., Nagare, R.P., Bindhya, S., Shetty, V., Manikandan, D., et al. (2018). Evaluation of a polymorphism in MYBPC3 in patients with anthracycline induced cardiotoxicity. *Indian Heart Journal* 70(2)**,** 319-322. doi: 10.1016/j.ihj.2017.07.001.

Visscher, H., Rassekh, S.R., Sandor, G.S., Caron, H.N., van Dalen, E.C., Kremer, L.C., et al. (2015). Genetic variants in SLC22A17 and SLC22A7 are associated with anthracycline-induced cardiotoxicity in children. *Pharmacogenomics* 16(10)**,** 1065-1076. doi: 10.2217/pgs.15.61.

Visscher, H., Ross, C.J.D., Rassekh, S.R., Barhdadi, A., Dubé, M.-P., Al-Saloos, H., et al. (2012). Pharmacogenomic Prediction of Anthracycline-Induced Cardiotoxicity in Children. *Journal of Clinical Oncology* 30(13)**,** 1422-1428. doi: 10.1200/jco.2010.34.3467.

Visscher, H., Ross, C.J.D., Rassekh, S.R., Sandor, G.S.S., Caron, H.N., van Dalen, E.C., et al. (2013). Validation of variants in SLC28A3 and UGT1A6 as genetic markers predictive of anthracycline‐induced cardiotoxicity in children. *Pediatric Blood & Cancer* 60(8)**,** 1375-1381. doi: 10.1002/pbc.24505.

Vivenza, D., Feola, M., Garrone, O., Monteverde, M., Merlano, M., and Lo Nigro, C. (2018). Role of the Renin-Angiotensin-Aldosterone System and the Glutathione S-Transferase Mu, Pi and Theta Gene Polymorphisms in Cardiotoxicity after Anthracycline Chemotherapy for Breast Carcinoma. *The International Journal of Biological Markers* 28(4)**,** 336-347. doi: 10.5301/jbm.5000041.

Völler, S., Boos, J., Krischke, M., Würthwein, G., Kontny, N.E., Boddy, A.V., et al. (2015). Age-Dependent Pharmacokinetics of Doxorubicin in Children with Cancer. *Clinical Pharmacokinetics* 54(11)**,** 1139-1149. doi: 10.1007/s40262-015-0272-4.

Vulsteke, C., Pfeil, A.M., Maggen, C., Schwenkglenks, M., Pettengell, R., Szucs, T.D., et al. (2015). Clinical and genetic risk factors for epirubicin-induced cardiac toxicity in early breast cancer patients. *Breast Cancer Research and Treatment* 152(1)**,** 67-76. doi: 10.1007/s10549-015-3437-9.

Wang, X., Chen, Y., Hageman, L., Singh, P., Landier, W., Blanco, J.G., et al. (2019). Risk prediction of anthracycline-related cardiomyopathy (AC) in childhood cancer survivors (CCS): A COG-ALTE03N1 and CCSS report. *Journal of Clinical Oncology* 37(15_suppl)**,** 10015-10015. doi: 10.1200/JCO.2019.37.15_suppl.10015.

Wang, X., Liu, W., Sun, C.-L., Armenian, S.H., Hakonarson, H., Hageman, L., et al. (2014). Hyaluronan Synthase 3 Variant and Anthracycline-Related Cardiomyopathy: A Report From the Children's Oncology Group. *Journal of Clinical Oncology* 32(7)**,** 647-653. doi: 10.1200/jco.2013.50.3557.

Wang, X., Singh, P., Zhou, L., Sharafeldin, N., Landier, W., Hageman, L., et al. (2023). Genome-Wide Association Study Identifies ROBO2 as a Novel Susceptibility Gene for Anthracycline-Related Cardiomyopathy in Childhood Cancer Survivors. *Journal of Clinical Oncology* 41(9)**,** 1758-1769. doi: 10.1200/jco.22.01527.

Wang, X., Sun, C.-L., Quinones Lombrana, A., Singh, P., Landier, W., Hageman, L., et al. (2015). CELF4 variant and Anthracycline-related Cardiomyopathy (anth-card) – A COG Study (ALTE03N1). *Journal of Clinical Oncology* 33(15_suppl)**,** 10066-10066. doi: 10.1200/jco.2015.33.15_suppl.10066.

Wang, X., Sun, C.-L., Quiñones-Lombraña, A., Singh, P., Landier, W., Hageman, L., et al. (2016). CELF4 Variant and Anthracycline-Related Cardiomyopathy: A Children’s Oncology Group Genome-Wide Association Study. *Journal of Clinical Oncology* 34(8)**,** 863-870. doi: 10.1200/jco.2015.63.4550.

Wasielewski, M., van Spaendonck-Zwarts, K.Y., Westerink, N.-D.L., Jongbloed, J.D.H., Postma, A., Gietema, J.A., et al. (2014). Potential genetic predisposition for anthracycline-associated cardiomyopathy in families with dilated cardiomyopathy. *Open Heart* 1(1). doi: 10.1136/openhrt-2014-000116.

Weiss, J.R., Kopecky, K.J., Godwin, J., Anderson, J., Willman, C.L., Moysich, K.B., et al. (2006). Glutathione S-transferase (GSTM1, GSTT1 and GSTA1) polymorphisms and outcomes after treatment for acute myeloid leukemia: pharmacogenetics in Southwest Oncology Group (SWOG) clinical trials. *Leukemia* 20(12)**,** 2169-2171. doi: 10.1038/sj.leu.2404421.

Windsor, R.E., Strauss, S.J., Kallis, C., Wood, N.E., and Whelan, J.S. (2012). Germline genetic polymorphisms may influence chemotherapy response and disease outcome in osteosarcoma: A pilot study. *Cancer* 118(7)**,** 1856-1867. doi: <https://dx.doi.org/10.1002/cncr.26472>.

Wojnowski, L., Kulle, B., Schirmer, M., Schlüter, G., Schmidt, A., Rosenberger, A., et al. (2005). NAD(P)H Oxidase and Multidrug Resistance Protein Genetic Polymorphisms Are Associated With Doxorubicin-Induced Cardiotoxicity. *Circulation* 112(24)**,** 3754-3762. doi: 10.1161/circulationaha.105.576850.

Yan, K., Dionne, F., Rassekh, S., Ross, C., and Carleton, B. (2020). Economic evaluation of pharmacogenomic testing in pediatric oncology patients treated with anthracyclines. *52nd Congress of the International Society of Paediatric Oncology, SIOP* 67(SUPPL 4)**,** no pagination.

Yunis, L.K., Linares‐Ballesteros, A., Aponte, N., Barros, G., García, J., Niño, L., et al. (2022). Pharmacogenetics of ABCB1, CDA, DCK, GSTT1, GSTM1 and outcomes in a cohort of pediatric acute myeloid leukemia patients from Colombia. *Cancer Reports* 6(3). doi: 10.1002/cnr2.1744.

Zolk, O., von dem Knesebeck, A., Graf, N., Simon, T., Hero, B., Abdul-Khaliq, H., et al. (2022). Cardiovascular Health Status And Genetic Risk In Survivors of Childhood Neuroblastoma and Nephroblastoma Treated With Doxorubicin: Protocol of the Pharmacogenetic Part of the LESS-Anthra Cross-Sectional Cohort Study. *JMIR Research Protocols* 11(2). doi: 10.2196/27898.
